# Crosstalk between H2A variant-specific modifications impacts vital cell functions

**DOI:** 10.1371/journal.pgen.1009601

**Published:** 2021-06-04

**Authors:** Anna Schmücker, Bingkun Lei, Zdravko J. Lorković, Matías Capella, Sigurd Braun, Pierre Bourguet, Olivier Mathieu, Karl Mechtler, Frédéric Berger

**Affiliations:** 1 Gregor Mendel Institute, Austrian Academy of Sciences, Vienna BioCenter, Vienna, Austria; 2 Biomedical Center, Department of Physiological Chemistry, Ludwig-Maximilians-University of Munich, Planegg-Martinsried, Germany; 3 International Max Planck Research School for Molecular and Cellular Life Sciences, Planegg-Martinsried, Germany; 4 CNRS, Université Clermont Auvergne, Inserm, Génétique Reproduction et Développement, Clermont-Ferrand, France; Institute of Functional Epigenetics, GERMANY

## Abstract

Selection of C-terminal motifs participated in evolution of distinct histone H2A variants. Hybrid types of variants combining motifs from distinct H2A classes are extremely rare. This suggests that the proximity between the motif cases interferes with their function. We studied this question in flowering plants that evolved sporadically a hybrid H2A variant combining the SQ motif of H2A.X that participates in the DNA damage response with the KSPK motif of H2A.W that stabilizes heterochromatin. Our inventory of PTMs of H2A.W variants showed that *in vivo* the cell cycle-dependent kinase CDKA phosphorylates the KSPK motif of H2A.W but only in absence of an SQ motif. Phosphomimicry of KSPK prevented DNA damage response by the SQ motif of the hybrid H2A.W/X variant. In a synthetic yeast expressing the hybrid H2A.W/X variant, phosphorylation of KSPK prevented binding of the BRCT-domain protein Mdb1 to phosphorylated SQ and impaired response to DNA damage. Our findings illustrate that PTMs mediate interference between the function of H2A variant specific C-terminal motifs. Such interference could explain the mutual exclusion of motifs that led to evolution of H2A variants.

## Author summary

DNA is wrapped around nucleosomes constituted by histones. Variants of histones diversified during evolution. The evolutionary path that led to selection of C-terminal motifs specific of each class of H2A variant is largely unknown. Here we show that cell cycle dependent kinases phosphorylate the KSPK motif identifying the heterochromatin-associated variant H2A.W that evolved in vascular plants. In all eukaryotes, H2A.X carries the SQ motif that is phosphorylated in response to DNA damage. Assembling both motifs in the C-terminal tail is rarely seen in hybrid H2A.W/X variants that evolved in a few plant species. Genetic manipulations in Arabidopsis show that this is possible only if phosphorylation of the KSPK motif is prevented. A synthetic strategy using yeast shows that KSPK phosphorylation interferes with the recognition of the reader of phosphorylated SQ and hampers with DNA damage response. This study proposes that interference between the function carried by C-terminal motifs led to their mutual exclusion achieving evolution of distinct classes of H2A variants.

## Introduction

Histones represent the major protein component of chromatin. Histone variants evolved in all core histone families and acquired comparable properties in a convergent manner [1–4].These variants play major roles in cell fate decisions, development, and disease [5–7]. Most multicellular eukaryotes contain three types of H2A variants: H2A, H2A.Z and H2A.X, which are distinguished by specific substitutions in the core domains as well as specific signatures in the C-terminal tails [8–10]. The variant H2A.X is defined by the motif SQ[E/D]Φ present within the C-terminal tail (where Φ stands for a hydrophobic amino acid). The serine residue of this motif is phosphorylated during the early phase of the DNA damage response [7,11–16]. In plants and yeast, serine phosphorylation of SQ[E/D]Φ is also essential for DDR [17,18].

In vascular plants, the family of H2A.W variants is characterized by the C-terminal KSPK motif [19–21]. H2A.W exclusively occupy constitutive heterochromatin and consists of three isoforms in *Arabidopsis* (H2A.W.7, H2A.W.6 and H2A.W.12). H2A.W.6 and H2A.W.7 are ubiquitously expressed and present in slightly different locations with H2A.W.7 being more abundant than H2A.W.6 in patches of heterochromatin in chromosome arms [18]. H2A.W.12 is expressed only during the late phase of reproductive development and is less abundant than the two other variants [20]. H2A.W confers distinct properties to the nucleosome through differences in its primary amino acid sequence in the L1 loop, the docking domain, and the KSPK motif in the extended C-terminal tail [22,23]. Due to the expected location of the H2A.W C-terminus at the nucleosome dyad (entry/exit site of the DNA into the nucleosome), the KSPK motif is placed in a functionally significant area [24] where it interacts with the linker DNA [23].

Distinct C-terminal motifs present on H2A variants do not usually co-occur, although notable but rare exceptions to this rule exist in animals and plants. *Drosophila* H2A.V combines the SQ[E/D]Φ motif with properties of H2A.Z [25]. Several species of seed-bearing plants possess a subtype of H2A.W that also harbors a SQ motif [18]. In *Arabidopsis*, H2A.X is largely excluded from constitutive heterochromatin, which is occupied by the variant H2A.W.7 that carries both the SQ and KSPK motifs. H2A.X and H2A.W.7 are essential to mediate the response to DNA damage in *Arabidopsis*, but variants similar to H2A.W.7 are present only in a restricted number of flowering plant species [18], suggesting that co-occurrence of both motifs has been counter-selected during evolution. What led to the mutual exclusion of C-terminal motifs during H2A variant evolution has remained unclear, but one reason could be incompatibility between post-translational modifications (PTMs) on variant specific motifs. The co-occurrence of the SQ and KSPK motifs in the C-terminal tail of H2A.W.7 provides a model to test this hypothesis.

Here, we provide an inventory of PTMs of H2A.W variants in *Arabidopsis*, including some that are specific of subtypes of H2A.W variants. We show that cyclin dependent kinases (CDKs) phosphorylate the serine of the KSPK motif of both H2A.W.6 and H2A.W.7 *in vitro In planta*, H2A.W.7 is only phosphorylated on the SQ motif and not on the KSPK motif. Notably, H2A.W.7 carrying a phosphomimetic KDPK motif shows impaired DNA damage response comparable to the loss of function mutant deprived of H2A.W.7. Through a synthetic approach in fission yeast, we show that phosphorylation of the KSPK motif prevents the phosphorylated SQ motif to be bound that the BRCT domain protein Mdb1, which is required to trigger DNA damage response. Additional genetic experiments in *Arabidopsis* further support that the absence of KSPK phosphorylation in H2A.W.7 is essential for the SQ motif to mediate DNA damage response. Hence, PTMs of C-terminal motifs of the H2A.W.7 variant interfere with the function of H2A.W.7 in DNA damage response. We thus observe a functional interference between PTMs carried by H2A-variant-specific motifs. This provides a possible explanation for the mutual exclusion of C-terminal motifs participating in the selection of distinct classes of H2A variants.

## Results

### H2A.W.6 and H2A.W.7 display distinct patterns of modifications at their C-terminal tails

Different types and combinations of PTMs of core histones coordinate the recruitment of proteins that dictate chromatin configuration and consequently regulate genome integrity and genome expression [5,7,16,17,26–28]. Non-centromeric H3 variants share strong sequence homology and, with a few exceptions [29–31], are subjected to the same repertoire of modifications. In contrast, sequence homology between H2A variants, particularly at their N- and C-terminal tails, is much less pronounced [9,19]. This provides opportunities for deposition of distinct patterns of modifications for each type of H2A variant [7,32].

**Fig 1:**
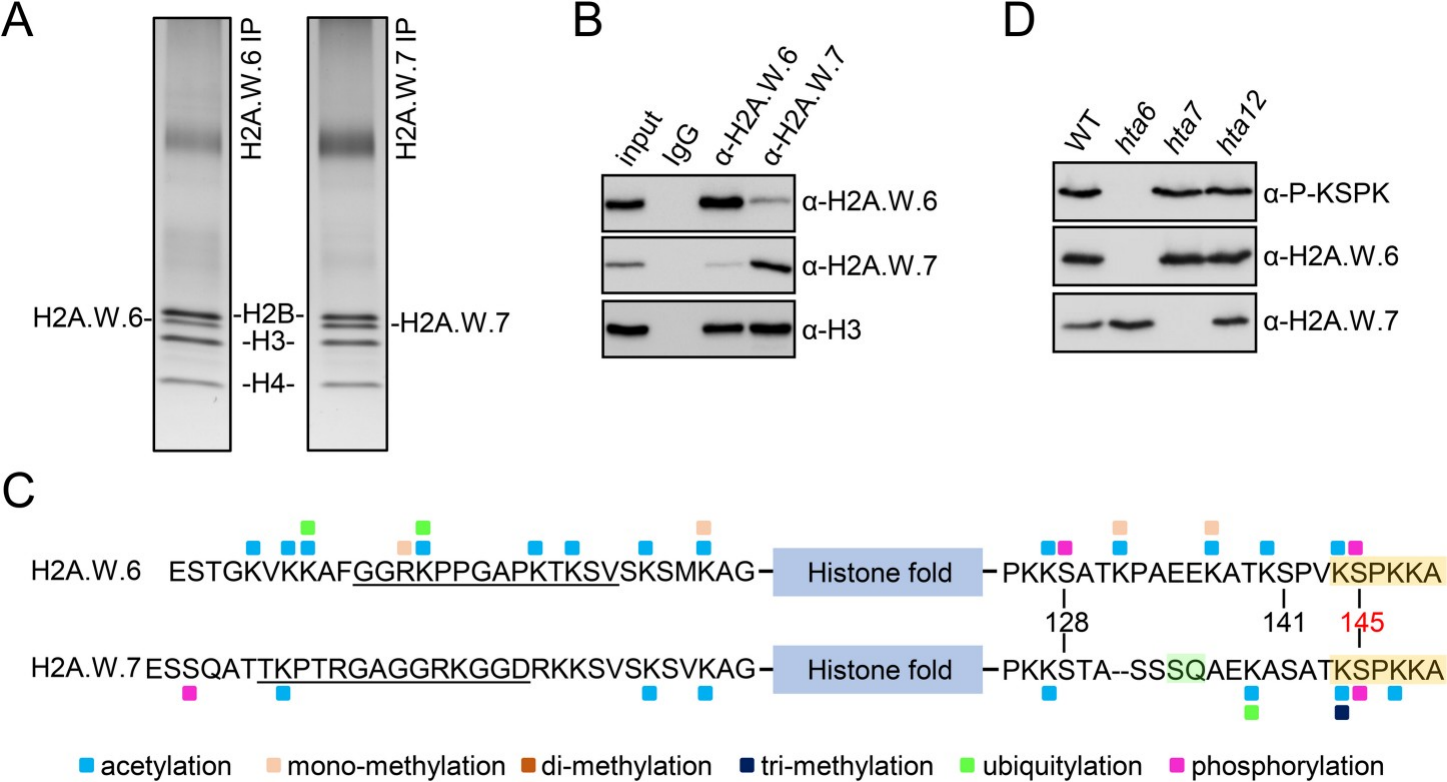
H2A.W.6 and H2A.W.7 carry distinct PTMs. (A) Silver stained gels of H2A.W.6 and H2A.W.7 mononucleosomes immunoprecipitated from MNase digested nuclear extracts from leaves. Bands corresponding to H2A.W.6 and H2A.W.7 were excised and analyzed by mass spectrometry (MS). (B) Western blot analysis of immunoprecipitates obtained with H2A.W.6 and H2A.W.7 specific antibodies from MNase digested WT nuclei. Note that H2A.W.6 and H2A.W.7 nucleosomes contain small amounts of H2A.W.7 and H2A.W.6, respectively. (C) Summary of all PTMs detected on H2A.W.6 and H2A.W.7. Amino acid sequence of N- and C-terminal tails of H2A.W.6 and H2A.W.7 are indicated with the conserved H2A.W KSPK motif and H2A.W.7 SQ motif highlighted in orange and green, respectively. The blue box indicates the histone fold domain. Post-translational modifications detected by MS are color-coded as indicated at the bottom. Peptides used to obtain variant specific antibodies are underlined. (D) Western blot analysis of nuclear extract from twelve days old WT, *hta6*, *hta7*, and *hta12* mutant seedlings with antibodies against P-KSPK, H2A.W.6, and H2A.W.7.

We immunopurified mononucleosomes containing H2A.W.6 or H2A.W.7 from wild type (WT) leaves of *Arabidopsis* (Fig 1A and 1B) and performed qualitative MS analysis to identify PTMs associated with each isoform. In both isoforms, N- and C-terminal tails were modified at most lysine residues by acetylation and/or methylation (Fig 1C). A more complex set of modifications was found on the C-terminal tail of H2A.W.6, with prevalence of lysine acetylation and serine phosphorylation (Fig 1C). Although H2A.W.6 and H2A.W.7 exist primarily as homotypic (two copies of either H2A.W.6 or H2A.W.7), heterotypic (one copy of each H2A.W.6 and H2A.W.7) nucleosomes can be identified [18,22] (Fig 1B). Importantly, H2A.W.6 and H2A.W.7 precipitated in respective and reciprocal immunoprecipitations contained similar sets of modifications (S1 Fig), suggesting that each variant isoform acquires distinct modification patterns independently of the nucleosome composition. Some of these modifications were specific to each isoform, in part due to the absence of conservation of the residues targeted by these modifications. Two lysine residues at the N-terminal tails of H2A.W.6 and H2A.W.7 present in a highly conserved sequence context (SVSKSMKAG vs. SVSKSVKAG) showed similar PTMs in both variants. In contrast, other lysine residues at the N-termini in a less conserved context displayed isoform-specific modifications (Fig 1C). On both variants, acetylation was detected on lysine residue 128 and 144, which are embedded in the same sequence context (Fig 1C). Leaves and flowers showed similar patterns of lysine modifications at the C-terminal tail, but they differed in their range and abundance. The observed differences may be due to different cell types and cell cycle dynamics in these two tissue types. The two serine residues (S129 and S145) at the C-terminal tail that are conserved in both H2A.W variants were phosphorylated on H2A.W.6 in leaves, as previously reported [33]. Based on spectral counting (S1B Fig), the more abundant phosphorylation was S145. In flowers, only S145 was detected on H2A.W.6 (S1 Fig). Phosphorylation of the S145 was detected in a single spectrum originating from H2A.W.7 (S1B Fig). Overall, the repertoire of PTMs detected on H2A.W.6 and H2A.W.7 differed markedly (Fig 1C). As S145 of the conserved KSPK motif is part of the functionally relevant C-terminal tail that protects the linker DNA [22], we focused our further analysis on S145 phosphorylation. We obtained an antibody that specifically binds phosphorylated S145 (hereafter P-KSPK) in both H2A.W.6 and H2A.W.7 *in vitro* (S2 Fig). Yet, consistent with the MS data, *in vivo* KSPK phosphorylation was only detected on H2A.W.6 (Fig 1C and 1D). Thus, one single MS spectrum detected on KSPK motif from H2A.W.7 represents a rare event that is not detectable by P-KSPK antibody. Our data suggest that phosphorylation of the KSPK motif is predominantly deposited on H2A.W.6 but not on H2A.W.7, which is phosphorylated on its SQ motif in response to DNA damage.

### The KSPK motif of H2A.W.7 is not phosphorylated upon DNA damage induction

**Fig 2:**
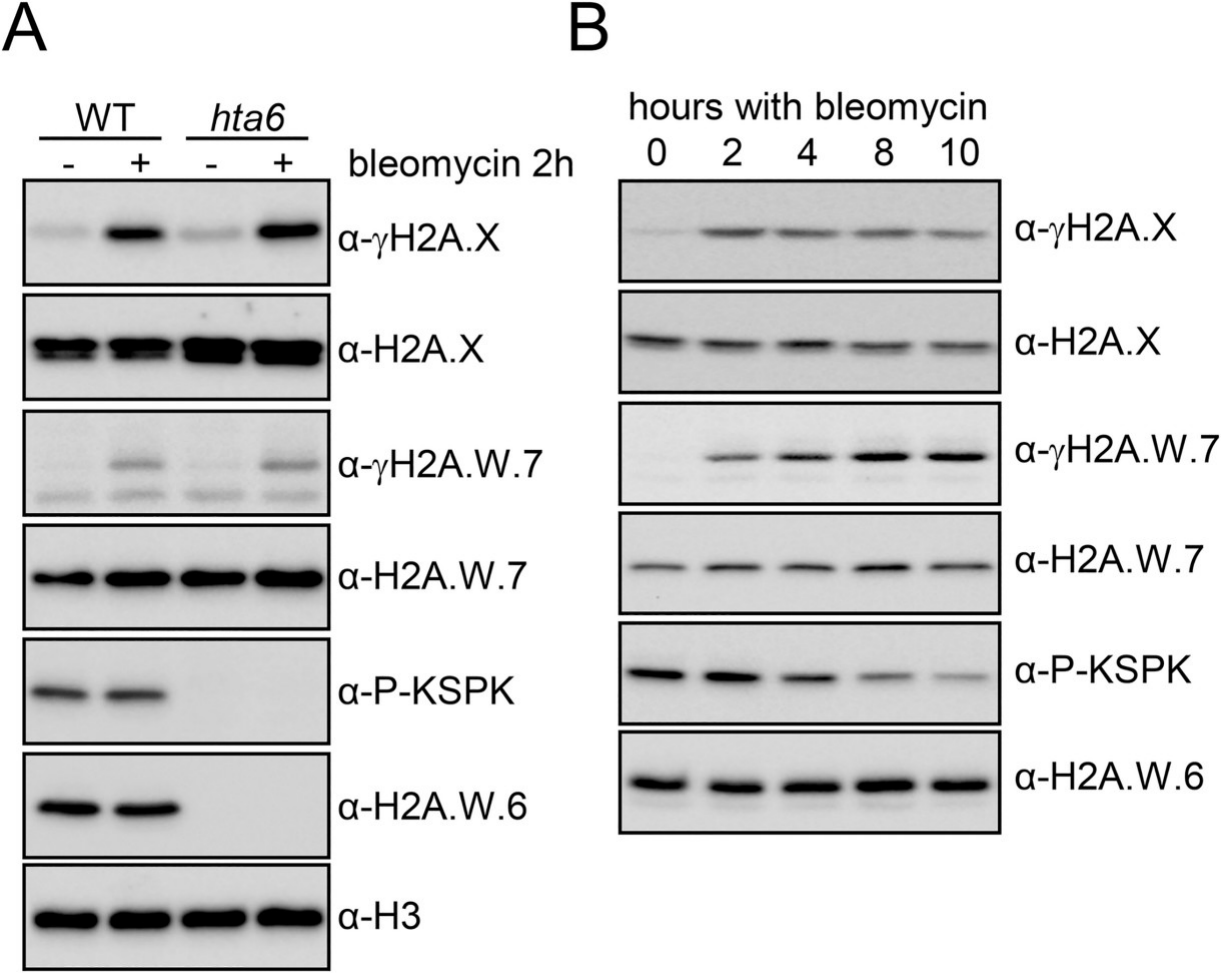
Phosphorylation of the H2A.W KSPK motif is not induced by DNA damage. (A) Phosphorylation of the KSPK motif on H2A.W.7 cannot be triggered by DNA damage. WT and *hta6* mutant seedlings were either mock or bleomycin (20 μg/ml) treated for two hours and nuclear extracts were analyzed by western blotting with indicated antibodies. (B) *Arabidopsis* WT seedlings were treated with 20 μg/ml of bleomycin for the indicated time periods and protein extracts were analyzed with the indicated antibodies.

The absence of phosphorylation of the KSPK motif in H2A.W.7 was surprising, and we hypothesized that it may take place in response to specific events. We investigated the modification of KSPK and SQ motifs in H2A.W.6, H2A.W.7, and H2A.X after induction of DNA damage by two hours treatment of *Arabidopsis* seedlings with bleomycin. In WT, bleomycin treatment induced phosphorylation of H2A.X and H2A.W.7 at the SQ motifs (γH2A.X and γH2A.W.7), as previously reported [18] (Fig 2A). To determine whether DNA damage can induce phosphorylation of KSPK on H2A.W.7, we also applied bleomycin treatment to *hta6* mutant seedlings, which are deprived of H2A.W.6 and only expressed H2A.W.7 (Fig 2A). Under these conditions, KSPK phosphorylation was not detected, suggesting that DDR does not induce phosphorylation of KSPK on H2A.W.7. In conclusion, DNA damage triggers phosphorylation of the SQ motif of H2A.X and H2A.W.7, but not of the KSPK motif of H2A.W.7.

### H2A.W.6 phosphorylation is cell cycle dependent and is mediated by cyclin dependent kinases

**Fig 3:**
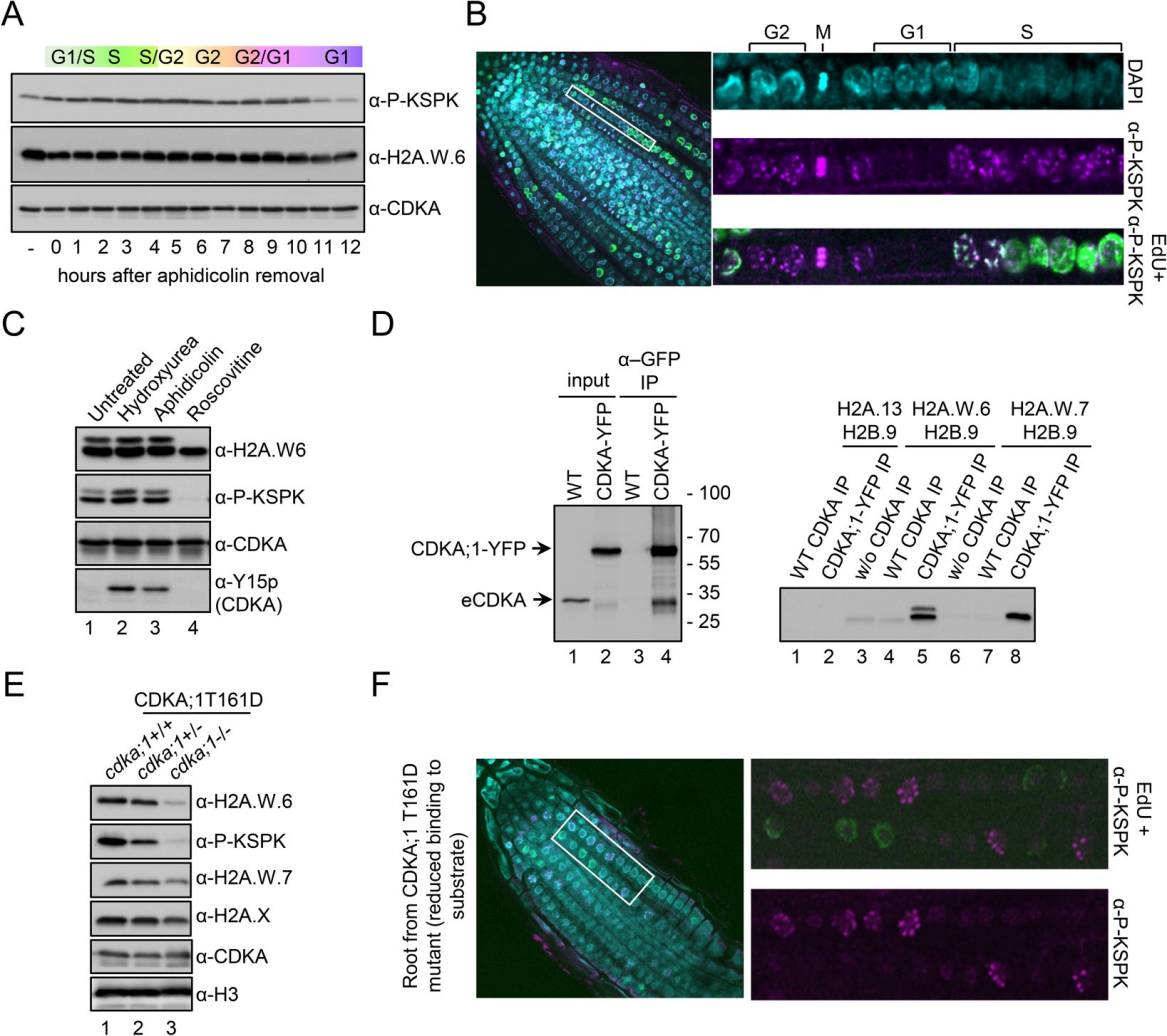
H2A.W.6 is phosphorylated in a cell cycle dependent manner by cyclin dependent protein kinases (CDK). (A) Phosphorylation of the KSPK motif is cell cycle dependent in tobacco BY-2 cell suspension culture. BY-2 cells were synchronized with 20 μg/ml aphidicolin for 24 hours. Protein extracts from samples taken in one-hour intervals after release of the block were analyzed by western blotting with the indicated antibodies. (B) Confocal images of WT root tips immunostained with H2A.W.6p antibody (magenta) after EdU (green) incorporation. Enlarged images of a row of cells in different cell cycle stages (indicated on the top) are shown on the right. (C) Protein extracts from *Arabidopsis* cell suspension treated with cell cycle inhibitors were analyzed by western blotting with the indicated antibodies. Inhibitory phosphorylation of CDK at tyrosine 15 (Y15) upon hydroxyurea and aphidicolin treatment (bottom panel, lanes 2 and 3) demonstrate the specificity and robustness of the assay. By contrast, roscovitine that inhibits the CDK activity directly by binding to the ATP binding pocket does not induce Y15 phosphorylation. (D) *Arabidopsis* CDKA;1-YFP was immunoprecipitated from whole cell extracts and detected with an anti-CDK antibody (left panel, lanes 2 and 4) and used in an *in vitro* kinase assay (right panel) with recombinant histone H2A-H2B dimers as indicated. Phosphorylation of the KSPK motif was detected by western blotting with P-KSPK antibody. Note that GFP beads do not precipitate wild-type CDKA;1 (left panel, lane 3) and consequently P-KSPK was not detected in kinase assays from these IPs (right panel, lanes 4 and 7). (E) Transgenic plants expressing weak hypomorphic mutant of CDKA;1, named D, in the *cdka;1* mutant background, were genotyped to identify heterozygous and homozygous plants (*cdka;1*+/- and *cdka;1*-/-). Nuclear protein extracts from 2-weeks old seedlings were analyzed by western blotting with the indicated antibodies. (F) CDKB;1, which is active at the G2/M transition, also phosphorylates the KSPK motif of H2A.W.6. Root tips of plants expressing the CDKA;1 T161D hypomorphic mutant that results in reduced substrate binding were immunostained with the P-KSPK antibody (magenta) after EdU incorporation (green). Enlarged images on the right demonstrate the presence of P-KSPK in G2 nuclei but not in S phase nuclei labeled with EdU. A single confocal section is shown.

We observed that prolonged exposure to bleomycin caused a marked decrease of KSPK phosphorylation of H2A.W.6 (Fig 2B). As DNA damage causes cell cycle arrest [34,35], we hypothesized that KSPK phosphorylation could be associated with cell cycle progression. Because our *Arabidopsis* cell suspension could not be synchronized, we used tobacco BY-2 cell suspension for cell cycle synchronization to address this question. In tobacco, six of the seven H2A.W isoforms contain the KSPK motif at the C-terminus, of which phosphorylation was detected by the P-KSPK antibody (S3A and S3B Fig). We analyzed H2A.W phosphorylation status in synchronized tobacco BY-2 cells over a twelve-hour time course after release from an aphidicolin-induced cell cycle block (S3C and S3D Fig). While H2A.W.6 levels were comparatively stable throughout the cell cycle, phosphorylation of KSPK remained stable during S and G2 but decreased during G1 phase (Figs 3A and S3E). We tested whether levels of phosphorylated H2A.W.6 also fluctuated in dividing cells of *Arabidopsis* root tips where pairs of small flat cells in G1 are easily distinguished from larger cells in G2 phase (Fig 3B). Cells in S phase were marked by a pulse of EdU incorporation. Consistent with results obtained with synchronized BY-2 cells, *Arabidopsis* root tip nuclei in S, M, and G2 phases showed high levels of H2A.W.6 phosphorylation, whereas this mark was not detected in G1 phase nuclei (Fig 3B). This fluctuation was not the result of changes in total levels of H2A.W.6, which remained relatively uniform throughout the cell cycle (S4A Fig). These results showed that phosphorylation of H2A.W.6 is dependent on cell cycle progression.

We next tested the impact of cell cycle inhibition on H2A.W.6 phosphorylation. S phase arrest by treatment with hydroxyurea or aphidicolin did not affect H2A.W.6 phosphorylation in *Arabidopsis* cell suspension cultures (Fig 3C, lanes 2 and 3, respectively). In contrast, when we inhibited cyclin-dependent kinases (CDKs) with roscovitine [36], phosphorylation of H2A.W.6 was almost undetectable (Fig 3C, lane 4). The specificity and concentration dependence of inhibition of H2A.W.6 phosphorylation by roscovitine (Figs 3C and S4B) prompted us to examine the *Arabidopsis* cyclin-dependent kinase CDKA;1, which is predominantly active at the G1 to S-phase transition [37,38]. We immunopurified CDKA;1-YFP from *Arabidopsis* seedlings and performed an *in vitro* kinase assay with recombinant H2A.W.6-H2B, H2A.W.7-H2B, and H2A-H2B dimers (Fig 3D). We observed strong phosphorylation of the H2A.W.6-H2B and H2A.W.7-H2B dimers but no signal in the H2A-H2B control (Fig 3D), demonstrating that CDKA;1 phosphorylates H2A.W.6 and H2A.W.7 *in vitro*. In homozygous mutant *cdka;1* plants with reduced CDKA;1 kinase activity [39,40], we detected very low levels of H2A.W.6 and as a consequence very low levels of its phosphorylated form (Fig 3E, lane 3). However, in heterozygous mutant *cdka;1/+* plants, the levels of H2A.W.6 were comparable to WT, yet levels of phosphorylated H2A.W.6 were strongly reduced (Fig 3E, lane 2). These results further supported that CDKA;1 is the main kinase responsible for H2A.W.6 modification. To detect if another cyclin dependent kinase activity is responsible for this PTM, we immunostained root tips from homozygous *cdka;1* plants with reduced CDKA;1 kinase activity. Only late G2/M-phase nuclei showed H2A.W.6 phosphorylation (Fig 3F). This suggested that another CDK phosphorylates the KSPK motif of H2A.W.6 in the absence of CDKA;1, which might be CDKB;1, as it is active at the G2/M transition [38,41].

### Cross talk between phosphorylation at the KSPK and SQ motifs in H2A.W.7

We demonstrated that CDKA;1 phosphorylates H2A.W.7 at the KSPK motif *in vitro* (Fig 3D); however, this modification was not detected *in planta* (Figs 1C, 1D and S2). We thus tested whether DNA damage induced phosphorylation of the SQ motif prevents KSPK phosphorylation in H2A.W.7. To test this idea phosphomimetic (SQ to DQ) mutant forms of H2A.W.7 were expressed in in *hta7* plants [18]. We did not detect KSPK phosphorylation on phosphomimetic (SQ to DQ) mutant forms of H2A.W.7 (S5A Fig). Additionally, KSPK phosphorylation was also not observed when SQ phosphorylation was prevented in *hta7* plants expressing non-phosphorylatable (SQ to AQ) (S5A Fig). Hence, neither the SQ motif nor its phosphorylation appear to interfere with KSPK phosphorylation in H2A.W.7.

**Fig 4:**
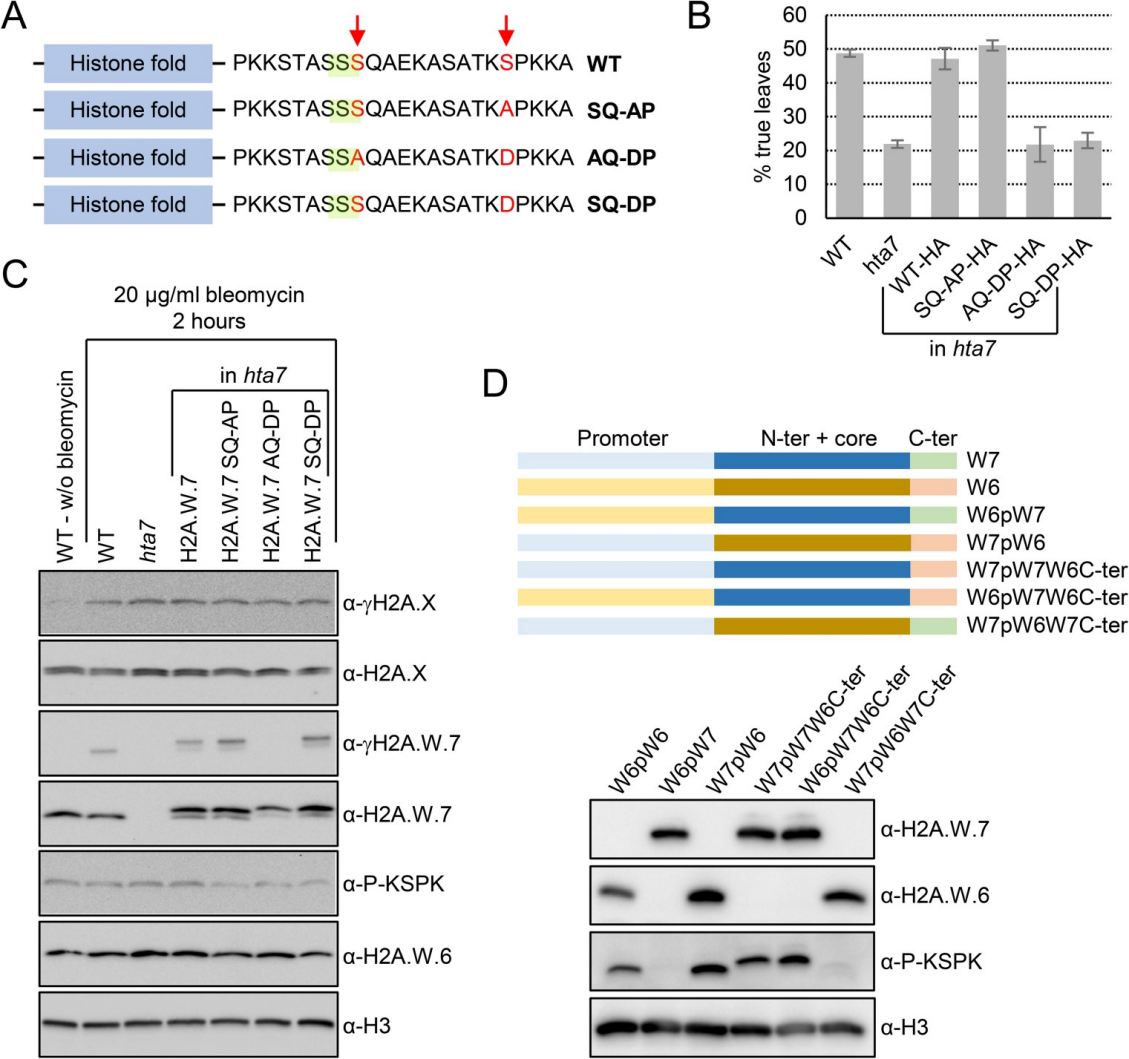
Phosphomimic of KSPK phosphorylation results in DNA damage sensitivity *in planta*. (A) Schematic diagrams of *Arabidopsis* H2A.W.7 variants containing mutated serin residues (in red) in the SQ (highlighted in green) and KSPK motifs. (B) Phosphomimic of the KSPK motif confers DNA damage sensitivity. Seeds from *h2a*.*w*.*7* mutants expressing HA-tagged WT H2A.W.7 or H2A.W.7 with mutations in the SQ and KSPK motifs were germinated in the presence of 50 mg/mL of zeocin and scored for true leaf development twelve days after germination. Data are represented as means ±SD of three independent experiments with n>400 seedlings. (C) Phosphomimic of the KSPK motif does not prevent SQ motif phosphorylation of H2A.W.7. Seedlings grown for 10 days on MS plates were treated for two hours with 20 μg/ml of bleomycin and nuclear extracts were analyzed by western blotting with the indicated antibodies. (D) Primary sequence of the H2A.W C-terminal tail determines phosphorylation outcome of the KSPK motif. Schemes of promoter, histone core domain and C-terminal tail swaps between H2A.W.6 and H2A.W.7 (top panel). Western blot analysis of expression and KSPK motif phosphorylation of nuclear extracts from plants expressing indicated H2A.W swap versions in mutant plants deprived from H2A.W (bottom panel).

To test if KSPK phosphorylation of H2A.W.7 interferes with DNA damage response *in planta*, we attempted to rescue the high sensitivity to DNA damage observed in *hta7* mutant by expressing WT H2A.W.7 or mutant forms that affect the KSPK motif (Fig 4A). We used a classical DNA damage response assay [42] to assess the functional importance of phosphorylation of these two motifs of H2A.W.7. In contrast to WT, the development of *hta7* mutant plants is retarded in the presence of DNA inducing agents, as measured by the production of the first pair of true leaves after seed germination (Fig 4B). The loss of function *hta7* mutant was rescued by WT H2A.W.7 and also by a non-phosphorylatable KAPK form of H2A.W.7 (Fig 4B). However, a mutant form of the protein that mimics the phosphorylation (KDPK) does not rescue the hypersentivity to DNA damage of the *hta7* mutant (Fig 4B). We concluded that the phosphorylation of the serine residue of the KSPK motif interferes with the DDR. Notably, mutations of the KSPK motif into either KAPK or KDPK did not affect SQ motif phosphorylation (Fig 4C). Thus, the presence of a negative charge at the KSPK motif interferes with an event downstream of SQ motif phosphorylation during DNA damage response. These results suggested that serine phosphorylation of KSPK of H2A.W.7 must be prevented to execute DNA damage response.

What are the factors preventing phosphorylation of KSPK in H2A.W.7? We hypothesized that the expression patterns of H2A.W.6 and H2A.W.7 differ, leading to exposure of these two variants to contrasting activities that deposit and maintain KSPK phosphorylation. Contrary to our hypothesis, we observed that phosphorylation of KSPK in H2A.W.7 was still absent even when expressed under the control of the H2A.W.6 promoter in the triple mutant *h2a*.*w-2* completely deprived of H2A.W [43] (Fig 4D). Furthermore, we observed phosphorylation of KSPK of the C-terminal tail of H2A.W.6 when fused to the core of H2A.W.7 irrespective of the promoter used (Fig 4D). Fusion of the H2A.W.7 C-terminal tail to H2A.W.6 core did not result in KSPK phosphorylation. These results suggested that differences in primary sequence of the C-terminal tail that distinguish H2A.W.7 from H2A.W.6 are determining factors for phosphorylation of the KSPK motif *in planta*. The fact that both variants are phosphorylated by CDKA;1 *in vitro* argues that *in planta*, unknown factors either determine a specific recognition of the C-terminal tail of H2A.W.6 as the only good substrate for phosphorylation or actively prevent phosphorylation of H2A.W.7.

### Cross talk between phosphorylation at the KSPK is mediated by proteins containing a BRCT domain that binds to phosphorylated SQ

**Fig 5:**
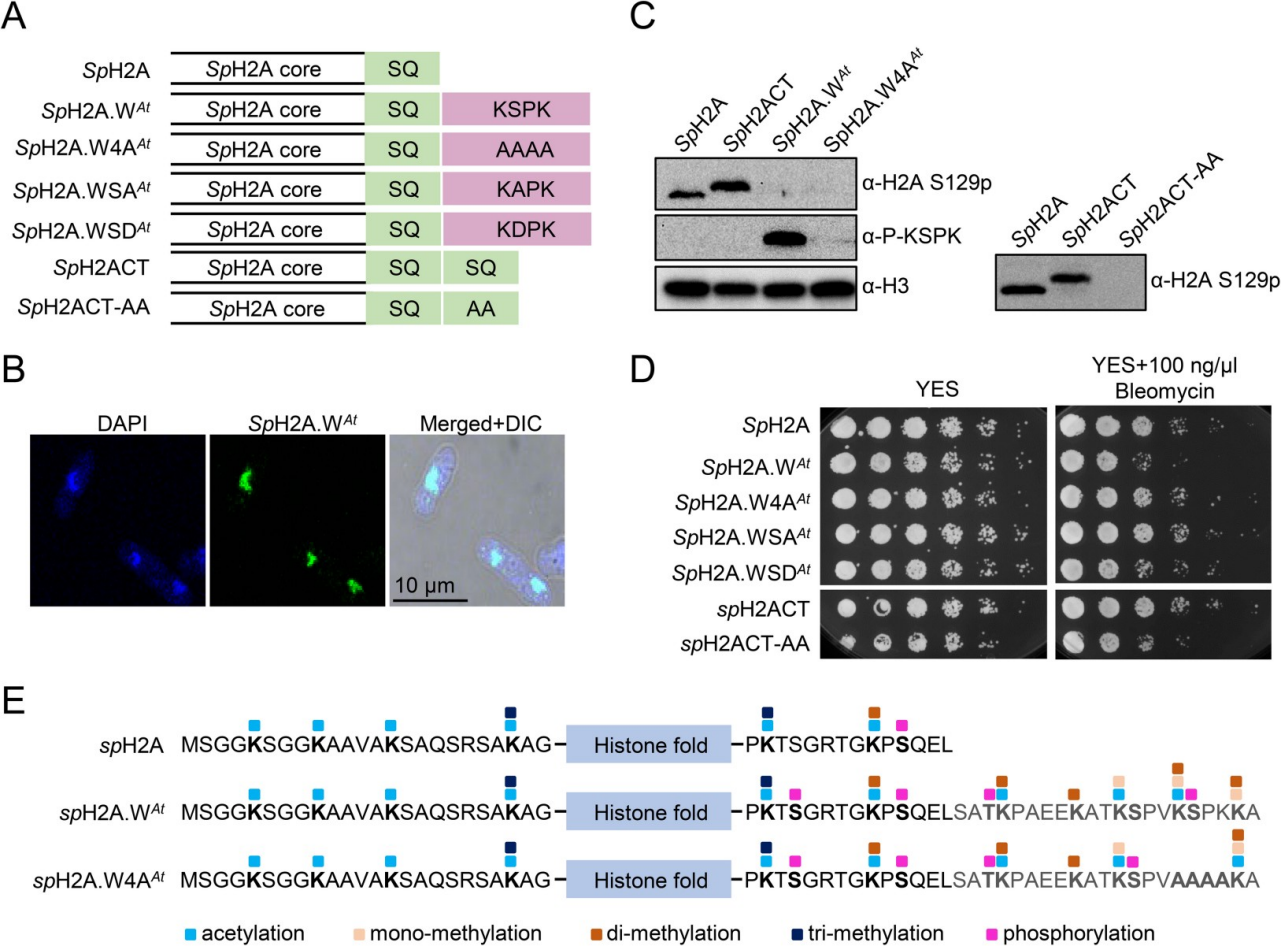
Phosphorylation of the KSPK motifs results in DNA damage sensitivity in yeast. (A) Schematic diagrams of fission yeast histone H2A (*Sp*H2A) and mosaic versions containing either a duplicated C-terminal tail (*Sp*H2ACT) or the C-terminal tail from *Arabidopsis* H2A.W.6 (*Sp*H2A.W^*At*^). Mutant versions in the SQ and KSPK motifs of the latter two constructs, *Sp*H2ACT-AA and *Sp*H2A.W4A^*At*^, are also depicted. (B) *Sp*H2A.W^*At*^ localizes to the nucleus in fission yeast. Confocal images of immunostained cells from exponential phase are shown. 4′,6-diamidino-2-phenylindole (DAPI) staining was used to visualize nuclei and differential interference contrast (DIC) for cell shape. (C) Analysis of *Sp*H2A S129 (SQ) and of *Sp*H2A.W^*At*^ (KSPK) phosphorylation in indicated yeast strains. Cells were collected during exponential growth and whole cell extracts were analyzed with the indicated antibodies, using anti-histone H3 antibody as loading control. The lack of SQ phosphorylation signal in strains expressing *Sp*H2A.W^*At*^, *Sp*H2A.W4A^*At*^, and *Sp*H2ACT-AA is due to the inability of the antibody to bind to the epitope. Phosphorylation of the SQ motif in these strains was confirmed by mass spectrometry (S5D Fig). (D) Phosphorylation of *Sp*H2A.W^*At*^ at the KSPK motif confers sensitivity to DNA damage. Serial dilutions of fission yeast cells expressing WT and indicated *Sp*H2A mosaic variants were spotted onto either YES or YES plates containing 100 ng/μl Bleomycin and incubated at 32°C for 3 days. (E) Summary of all PTMs detected on *Sp*H2A in WT and chimeric histone in indicated yeast strains. Amino acid sequence of N- and C-terminal tails of chimeric histone are indicated with the conserved *Sp*H2A SQ motif and H2A.W.6 KSPK motif respectively. The blue box indicates the histone fold domain. Post-translational modifications detected by MS are color-coded as indicated at the bottom.

To further investigate a potential crosstalk between phosphorylation of the H2A.W.7 SQ and KSPK motifs, we took a synthetic approach using the fission yeast *Schizosaccharomyces pombe*. Fission yeast possesses a relatively reduced repertoire of histone H2A variants, consisting of two H2A.X variants (*Sp*H2A.α and *Sp*H2A.β) and H2A.Z [44,45]. We modified both genes encoding *Sp*H2A by inserting the C-terminal tail of the *Arabidopsis* H2A.W.6 to the C-terminus of *Sp*H2A.α and *Sp*H2A.β that contains an SQ motif, and obtained the chimeric histone *Sp*H2A.W^*At*^ that possesses the motifs present in H2A.W.7 [23] (Figs 5A and S5B). In dividing cells, *Sp*H2A.W^*At*^ localized to the nucleus and was incorporated in chromatin (Figs 5B and S5C). We detected phosphorylation of *Sp*H2A.W^*At*^ at the KSPK motif but not in a control strain that expressed *Sp*H2A.W^*At*^ where KSPK was substituted by four alanine residues (*Sp*H2A.W4A^*At*^) (Fig 5A and 5C). Interestingly, phosphorylation at the KSPK and SQ motifs were both detected on *Sp*H2A.W^*At*^ by mass spectrometry (Figs 5E and S5D), demonstrating that *Sp*H2A.W^*At*^ phosphorylation did not prevent SQ phosphorylation. While the yeast strain expressing *Sp*H2A.W^*At*^ lacks H2A.X, we predicted that SQ phosphorylation of *Sp*H2A.W^*At*^ would be sufficient to respond to DNA damage. However, expression of *Sp*H2A.W^*At*^ caused hyper-sensitivity to DNA damage caused by treatment with bleomycin, camptothecin (CPT) or methyl methane sulfonate (MMS) (Figs 5D, S5E and S5F). This suggested that either extension of the C-terminal tail or modification of the KSPK motif interfered with mediation of DNA damage response by phosphorylation of the SQ motif. To test these two possibilities, we examined DNA damage sensitivity of strains expressing either *Sp*H2A.W^*At*^, the KSPK mutant *Sp*H2A.W4A^*At*^, a non-phosphorylatable (KAPK) mutant *Sp*H2A.WSA^*At*^, a mutant that mimics the phosphorylation (KDPK) *Sp*H2A.WSD^*At*^, and *Sp*H2A with a repeated wild type C-terminal tail (*Sp*H2ACT; contains two SQ motifs) or with alanine substitution of the second SQ motif (*Sp*H2ACT-AA) (Fig 5A). Phosphorylation of the SQ motifs was detected in all strains and none of these modifications resulted in sensitivity to DNA damage treatment, except for *Sp*H2A.W^*At*^ cells and phosphomimic mutant *Sp*H2A.WSD^*At*^ (Figs 5E and S5D–S5F). We thus concluded that the phosphorylation of KSPK motif is responsible for the increased sensitivity to DNA damage in the strain expressing *Sp*H2A.W^*At*^.

**Fig 6:**
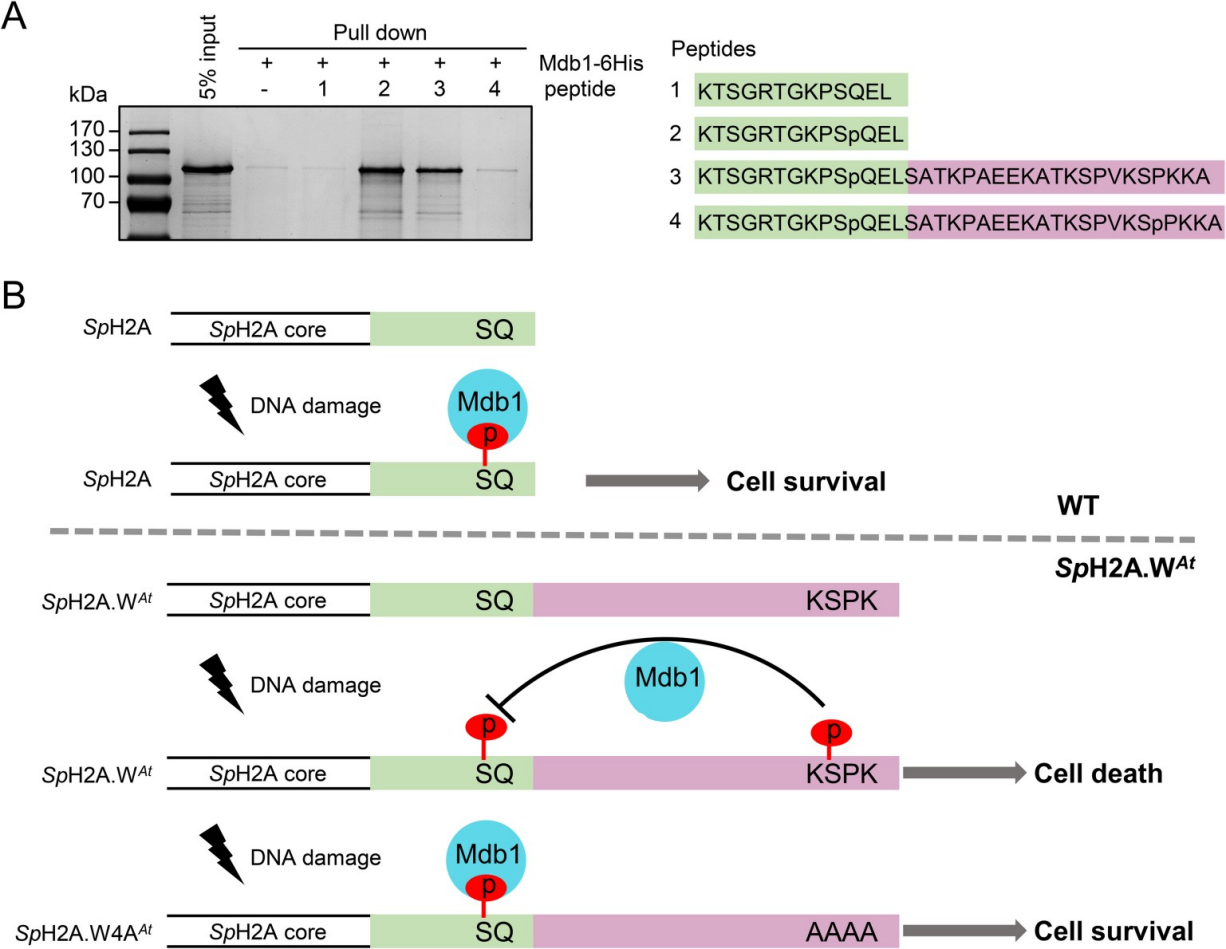
KSPK motif directly interferes with binding of Mdb1 to phosphorylated SQ in a phosphorylation dependent manner. (A) Mdb1 recombinant protein was expressed in *E*. *coli* BL21 and purified using Ni-NTA beads. Biotinylated peptides, as depicted on the right, corresponding to C terminus of *Sp*H2A, with unmodified (SQ) or S129 phosphorylation (SpQ), or SpQ with unmodified KSPK motif (SpQ+KSPK) or SpQ with S145 phosphorylation (SpQ+KSpPK), were incubated with streptavidin Dynabeads and Mdb1 recombinant protein. The eluates were analyzed by SDS-PAGE with Coomassie staining. (B) The model of crosstalk between SQ and KSPK phosphorylation. In WT cell, S129 phosphorylation site recruit Mdb1 as platform for downstream DDR in response to DNA damage. In *Sp*H2A.W^*At*^ cell, KSPK phosphorylation prevents the Mdb1 binding to SQ phosphorylation site, thus DNA cannot repair properly. In *Sp*H2A.W4A^*At*^ cell, the absence of KSPK phosphorylation allowed the Mdb1 binding to phosphorylated SQ to recruit DDR for DNA repair.

In animals, serine 139 (S139) phosphorylation at the SQ motif of H2A.X (γH2A.X) is sufficient to recruit the mediator of DNA damage checkpoint protein 1 (MDC1) [12]. In contrast, MDC1 binding is abolished if tyrosine 142 (Y142) of γH2A.X is phosphorylated. Thus, the succession of these two phosphorylation steps dictates the order of events at sites of DNA damage [15,16]. It was thus possible that phosphorylation of the KSPK motif specifically interfered with DDR events downstream of SQ phosphorylation. We performed a synthetic gene array (SGA) screen with a genome-wide mutant library of non-essential genes to identify candidate genes that display genetic interactions with *Sp*H2A.W^*At*^ in presence of the drug hydroxy urea (HU). From the cluster analysis, cluster 2 contained several groups of genes genetically interacting with *Sp*H2A.W^*At*^, including S/T protein kinases, S/T phosphatases and DNA repair proteins including BRCT domain proteins (S6 Fig). We focused on the BRCT domain protein Mdb1 that binds to phosphorylated SQ motif in fission yeast [46]. To address directly whether presence and/or phosphorylation of KSPK motif interferes with the interaction between Mdb1 and SQ phosphorylation, we performed *in vitro* pull-down assay with synthetic biotinylated peptides (Fig 6A) and recombinant Mdb1. Mdb1 was pulled down by the phosphorylated *Sp*H2A SQ peptide but not by the unmodified version (Fig 6A). Surprisingly, Mdb1 was able to bind the phosphorylated SQ motif in the presence of the KSPK motif but not if this latter motif was phosphorylated (Fig 6A). We concluded that phosphorylation of the KSPK motif specifically interfered with DNA damage response events downstream of SQ phosphorylation, namely binding of Mdb1 to the phosphorylated SQ motif (Fig 6B).

## Discussion

In addition to the well-characterized modifications associated with histone H3, we report a series of modifications at the N- and C-terminal tails, which are either common and specific to the H2A.W family or specific to each isoform. H2A.W.6 is phosphorylated at the serine residue in the KSPK motif in a cell cycle-dependent manner. The kinase CDKA;1, which is active during S phase, is the primary kinase that phosphorylates H2A.W.6 at KSPK, but this mark might also be reinforced by plant specific B-type CDKs active at the G2/M-phase transition [37,41]. It is interesting to note that the KSPK motif of linked histone H1 is also phosphorylated in a cell cycle dependent manner [47–49]. Enrichment in positively charged residues in H1 and H2A.W C-terminal tail likely promote their interaction with DNA. It is possible that the cell cycle dependent phosphorylation diminishes the positive charges of the histones and thereby weakens the contact with negatively charged DNA leading to increased chromatin accessibility.

When we compare modifications in H2A variants in *Arabidopsis* with other species, we find that some are plant-specific while others are found in other eukaryotes and show interesting parallels. Like S145 that is specific to H2A.W, S95 is only present in H2A and H2A.X in land plants. Phosphorylation of S95 of the replicative H2A variant has been observed in *Arabidopsis* and implicated in flowering time regulation [50]. Certain isoforms of H2A.W in maize are phosphorylated at S133 during mitosis and meiosis [51]. This residue corresponds to S129 of *Arabidopsis* H2A.W and is absent from H2A.Z, H2A.X, and three out of four H2As. In yeast, the corresponding S121 is phosphorylated by Bub1 to recruit shugoshin, an important step for centromere function in chromosome segregation [52–54]. This serine is replaced by threonine (T120) in human H2A and H2A.X, and its phosphorylation is associated with transcriptional activation and mitotic chromosome segregation [55–57]. Consistent with these data, we detected only a few events of S129 phosphorylation in H2A.W in samples originating from leaves or seedlings that contain low amounts of dividing cells. The neighboring lysine 119 of H2A and corresponding lysines residues of H2A.X, H2A.Z, and macroH2A are well-known ubiquitination sites, associated with transcriptional repression, gene silencing, and DNA damage response [7,58–66]. Also, phosphorylation of S137 in the KSPS site present in macroH2A1 is important for its function in heterochromatin [67]. Altogether our data illustrate that H2A variants extend the histone modification repertoire across eukaryotes beyond that of the extensively studied H3. Importantly, because H2A strongly impact nucleosome and chromatin properties, H2A variant specific modifications and their interplay extend the potential to modulate these properties, adding a new important layer of epigenetic complexity.

Surprisingly, although KSPK of H2A.W.7 can be phosphorylated *in vitro*, this modification on H2A.W.7 is barely detected *in planta*. Our data suggest that prevention of KSPK phosphorylation on H2A.W.7 originates from the sequence of the C-terminal tail of H2A.W.7 and not from its expression pattern or the core domain. We can speculate that *in planta*, other modifications of the C-terminal tail of H2A.W.7 prevent the action of the cyclin dependent kinases. A mutation scan of the residues that differentiate H2A.W.6 and H2A.W.7 could identify the origin of the discrimination by the kinase and or phosphatases that results in the contrasted phosphorylation of KSPK in these two variants. We addressed the biological significance of the lack of KSPK phosphorylation on H2A.W.7 with a synthetic approach by engineering the endogenous H2A from *S*. *pombe* to create a histone variant analogous to H2A.W.7. In yeast, we showed that phosphorylation of both SQ and KSPK motifs in the C-terminal tail of the synthetic variant *Sp*H2A.W^*At*^ resulted in DNA damage sensitivity. Phosphorylation of the KSPK (S145) motif prevents BRCT domain protein Mdb1 binding to phosphorylated *Sp*H2A in response to DNA damage. Experiments with transgenic *Arabidopsis* plants expressing combinations of phosphomimetic mutants in the SQ and KSPK motifs further support this conclusion. We propose that phosphorylation of the KSPK motif of H2A.W.7 prevents the recognition of the phosphorylated SQ motif of H2A.W.7 by an ortholog of Mdb1. This model might also be valid in *Arabidopsis* that expresses at least four BRCT domain-containing proteins that could function as MDC1 exist in *Arabidopsis*. However, the potential redundant function of these proteins prevents a genetic study of their role in the context of this study.

An extension of our previous phylogenetic analyses of H2A.W confirms that hybrid variants similar to H2A.W.7 are found only amongst flowering plants [18,23]. However, they do not share a common ancestor and the recruitment of the SQ motif to the C-terminus of H2A.W occurred in various unrelated species during the evolution of flowering plants (S7 Fig). It is present in many members of *Brassicaceae* but appears to have been lost in some species during evolution of this family. This suggests that hybrid H2A.W/X evolved several times independently in flowering but overall such events are rare (S7 Fig). The rarity of hybrid H2A.W/X variants is likely explained by the interference between PTMs of motifs of H2A.X and of H2A.W. More generally modifications on motifs specific of H2A variants might be more general and provides a clue to explain why these motifs are mutually exclusive. We propose that PTMs of these motifs mediate all or part of the function of the motifs themselves. The presence of two motifs side by side might interfere with the function of each other, leading to the emergence of distinct classes of H2A variants differentiated by specific functional motifs with distinct biological functions.

## Materials and methods

### Plant material

We used the following mutant lines *hta6* (SALK_024544.32; *h2a*.*w*.*6*), *hta7* (GK_149G05; *h2a*.*w*.*7*), and *hta12* (SAIL_667_G10; *h2a*.*w*.*12*) [20,43]. Transgenic lines expressing WT and hypomorphic SQ motif mutants of H2A.W.7 in the *hta7* mutant background as published [18]. The hypomorphic CDKA;1 D [39,40] and transgenic line expressing CDKA;1-YFP in *cdka;1* mutant background [68,69] were a kind gift from Dr. Arp Schnittger. Primers used for genotyping hypomorphic CDKA;1 D mutant (Fig 3) can be found in S1 Table. Unless otherwise stated, plants were grown in a fully automated climate chamber at 21°C, under long day conditions.

To construct mutant versions of H2A.W.7 containing either WT SQ motif or mutated SQ motif into AQ in combination with phosphomimic and non-phosphorylatable versions of the KSPK motif (KDPK or KAPK, respectively), a two-step PCR reaction was performed using previously described constructs expressing H2A.W.7 containing either the WT SQ or mutated AQ motif [18] as templates. For the first step amplification, the following primer pairs were used: W7-fw with either W7-Rev1KDP or W7-Rev1KAP (S1 Table). The second PCR step used the W7-Rev2KDP or W7-Rev2KAP (S1 Table) as reverse primers with the same forward primer as above. The mutant *HTA7* fragments were cut with *Bam*HI and *Xba*I and ligated into pCBK02. *hta7* mutant plants were transformed by the floral dip method and transgenic plants were selected on MS plates containing 10 μg/ml of phosphinothricin. The T3 and T4 homozygous seeds containing single transgene copy were used for experiments.

For swapping the promotors and tails of H2A.W.6 and H2A.W.7, cloning primers (S1 Table) were used to amplify the different fragments from genomic DNA of *Arabidopsis* seedlings. We created the GreenGate entry modules following previously published protocol [70]. The promotor fragments were introduced into the A-B empty vector, the full H2A.W.6 and H2A.W.7 as well as the N-terminal+core fragments were introduced into the C-D empty vector and the tail fragments were introduced into the D-E empty vectors. The Ub10 Terminator in the E-F entry vector and the selection based on seed coat YFP fluorescence were a gift from Yasin Dagdas laboratory. For the final GreenGate reaction pGGZ003_ccdB, the entry clones were combined with empty destination vector. The final constructs were transformed into the triple *hta6 hta7 hta12* mutant which is a complete knock-out line deprived of H2A.W [43].

### Isolation of nuclei, micrococcal nuclease (MNase) chromatin digestions, immunoprecipitation, and SDS-PAGE

Nuclei isolation, MNase digestion, and immunoprecipitation were performed as previously described [18] by using 4 grams of 2–3 week old leaves for each antibody. Isolated nuclei were washed once in 1 ml of N buffer (15 mM Tris-HCl pH 7.5, 60 mM KCl, 15 mM NaCl, 5 mM MgCl_2_, 1 mM CaCl_2_, 250 mM sucrose, 1 mM DTT, 10 mM ß-glycerophosphate) supplemented with protease and phosphatase inhibitors (Roche). After centrifuging for 5 min at 1,800 × *g* at 4°C, nuclei were re-suspended in N buffer to a volume of 1 ml. Twenty μl of MNase (0.1 u/μl) (SigmaAldrich) were added to each tube and incubated for 15 min at 37°C. During the incubation, nuclei were mixed 4 times by inverting the tubes. MNase digestion was stopped on ice by the addition of 110 μl of MNase stop solution (100 mM EDTA, 100 mM EGTA). Nuclei were lysed by the addition of 110 μl of 5 M NaCl (final concentration of 500 mM NaCl). The suspension was mixed by inverting the tubes and they were then kept on ice for 15 min. Extracts were cleared by centrifugation for 10 min at 20,000 × *g* at 4°C. Supernatants were collected and centrifuged again as above. For each immunoprecipitation extract, an equivalent of 4 g of leaf material was used, usually in a volume of 1 ml. To control MNase digestion efficiency, 200 μl of the extract were kept for DNA extraction. Antibodies, including non-specific IgG from rabbit, were bound to protein A magnetic beads (GE Healthcare) and then incubated with MNase extracts over night at 4°C. Beads were washed 2 times with N buffer without sucrose containing 300 mM NaCl, followed by 3 washes with N buffer containing 500 mM NaCl without sucrose, and 1 wash with N buffer without sucrose, containing 150 mM NaCl. Beads were incubated 2 times with 15 μl of hot loading buffer for 5 min and supernatants were removed and combined. Proteins were resolved on 4–20% gradient gels (Serva) and silver stained.

### Generation of antibodies, isolation of nuclei, SDS-PAGE, and western blotting

Antibodies against H2A.X, H2A.W.6, H2A.W.7, γH2A.X, and γH2A.W.7 have been described [18,20]. Antibodies against the H2A.W.6 phosphopeptide (CEEKATKSPVKSpPKKA) were raised in rabbits (Eurogentec) and purified by peptide affinity column. Purified IgG fractions were tested for specificity on peptide arrays containing serial dilutions of non-phosphorylated and phosphorylated peptides (phospho-serine specific antibodies).

Nuclei for western blot analyses were prepared from 300 mg of tissue (10–12 day old treated seedlings) as described [18]. Tissue was frozen in liquid nitrogen and disrupted in 15 ml Falcon tubes by rigorous vortexing with 5 small ceramic beads. Ten ml of nuclei isolation buffer (NIB; 10 mM MES-KOH pH 5.3, 10 mM NaCl, 10 mM KCl, 250 mM sucrose, 2.5 mM EDTA, 2.5 mM ß-mercaptoethanol, 0.1 mM spermine, 0.1 mM spermidine, 0.3% Triton X-100), supplemented with protease and phosphatase inhibitors (Roche) were added followed by short vortexing to obtain a fine suspension. The suspension was filtered through 2 layers of Miracloth (Merck Millipore) into 50 ml Falcon tubes, followed by washing the Miracloth with NIB. The sample was centrifuged at 3,000 rpm for 5 min at 4°C. The pellet was resuspended in NIB buffer and transferred into an Eppendorf tube. The sample was centrifuged for 2 min at 4°C at full speed and the resulting pellet was resuspended in 0.3 × PBS in 1 × Laemmli loading buffer. The sample was then boiled for 5 min and immediately centrifuged for 2 min at maximum speed to remove starch and other large particles. For western blot analyses, 10 μl for histone variants, 20 μl for histone modifications, and 10 μl for H3, used as a loading control, were loaded per lane. Western blots with phospho-specific antibodies were performed in solutions containing TBS instead of PBS and, after blocking, the membranes were incubated with primary antibody in TBS without milk.

Antibodies against H2A.X, H2A.W.6, H2A.W.7, γH2A.X, and γH2A.W.7 [18,20], as well as P-KSPK (this work), H3 (ab1791, Abcam), CDK (PSTAIR, Sigma P7962), and CDKY15p (Cell Signaling Technology) were used at 1:1,000 dilution and secondary antibodies, HRP conjugated goat anti-rabbit and goat anti-mouse, at 1:10,000. Signals were detected using the chemiluminescence kit (ThermoFisher), recorded using an ImageDoc instrument (BioRad), exported to Photoshop, and prepared for publication. Quantification of western blots was done with ImageLab 5.2 software (BioRad) using the volume tool. For detection of Y15 phosphorylation of CDK, the manufacturer’s protocol was followed.

### Nano LC-MS/MS analysis

Histone bands corresponding to H2A.W.6/H2A.W.7 from *Arabidopsis* and WT and modified versions of H2A from fission yeast strains were excised from silver stained gels, reduced, alkylated, in-gel trypsin, LysC, and subtilisin digested, and processed for MS. The nano HPLC system used was a Dionex UltiMate 3000 HPLC RSLC (Thermo Fisher Scientific, Amsterdam, Netherlands) coupled to a Q Exactive HF mass spectrometer (Thermo Fisher Scientific, Bremen, Germany), equipped with a Proxeon nanospray source (Thermo Fisher Scientific, Odense, Denmark). Peptides were loaded onto a trap column (Thermo Fisher Scientific, Amsterdam, Netherlands, PepMap C18, 5 mm × 300 μm ID, 5 μm particles, 100 Å pore size) at a flow rate of 25 μl/min using 0.1% TFA as the mobile phase. After 10 min, the trap column was switched in line with the analytical column (Thermo Fisher Scientific, Amsterdam, Netherlands, PepMap C18, 500 mm × 75 μm ID, 2 μm, 100 Å). Peptides were eluted using a flow rate of 230 nl/min, and a binary 1-hour gradient.

The Q Exactive HF mass spectrometer was operated in data-dependent mode, using a full scan (m/z range 380–1500, nominal resolution of 60,000, target value 1E6) followed by MS/MS scans of the 10 most abundant ions. MS/MS spectra were acquired using normalized collision energy of 27%, isolation width of 1.4 m/z, resolution of 30,000 and the target value was set to 1E5. Precursor ions selected for fragmentation (exclude charge state 1, 7, 8, >8) were put on a dynamic exclusion list for 20 s. Additionally, the minimum AGC target was set to 5E3 and intensity threshold was calculated to be 4.8E4. The peptide match feature was set to preferred and the exclude isotopes feature was enabled. For peptide identification, the RAW files were loaded into Proteome Discoverer (version 2.1.0.81, Thermo Scientific). All hereby created MS/MS spectra were searched using Mascot 2.2.7 against a database which contains all histone variants from *Arabidopsis thaliana*. The following search parameters were used: Beta-methylthiolation on cysteine was set as a fixed modification, oxidation on methionine, deamidation on asparagine and glutamine, acetylation on lysine, phosphorylation on serine, threonine and tyrosine, methylation and di-methylation on lysine and arginine and tri-methylation on lysine were set as variable modifications. Monoisotopic masses were searched within unrestricted protein masses for tryptic enzymatic specificity. The peptide mass tolerance was set to ±5 ppm and the fragment mass tolerance to ±0.03 Da. The result was filtered to 1% FDR at the peptide level using the Percolator algorithm integrated in Thermo Proteome Discoverer and additional a minimum Mascot score of 20. In addition, we have checked the quality of the MS/MS spectra manually. The localization of the phosphorylation sites within the peptides was performed with ptmRS using a probability threshold of minimum 75 [71].

### DNA damage sensitivity assays

For the true leaf assay, sterilized seeds were put on MS plates containing 50 μg/ml of zeocin (Invitrogen) and, after stratification at 4°C for 3 days, germinated under long day conditions. Development of true leaves was scored 12 days after germination. For analysis of H2A.W.7, H2A.X, and H2A.W.6 phosphorylation in response to DNA damage, 300 mg of twelve-day old seedlings germinated and grown on MS plates under long day conditions were treated in liquid MS medium with 20 μg/ml bleomycin (Calbiochem) for the indicated time periods or for 2 hours with 20 μg/ml bleomycin. After treatment, seedlings were removed from medium and shock frozen in liquid nitrogen for nuclei isolation and western blot analysis as described above.

### Cell cycle synchronization of BY-2 cell suspension culture with aphidicolin

The BY-2 cell suspension culture was sub-cultured every 2 weeks by adding 1 ml of the previous culture to 50 ml fresh BY-2 media. The culture was kept in the dark and under constant shaking at 130 rpm at room temperature. For cell cycle synchronization, a previously published protocol was followed [72]. In short, 1.5 ml of stationary BY-2 cells were added to 95 ml of fresh BY-2 media and grown for 5 days at 27°C. This culture was diluted 4 times with fresh BY-2 media and then treated with 20 μg/ml aphidicolin (1 mg/ml; SigmaAldrich) for 24 hours at 27°C to reach a cell cycle block. To release the cells from this block, the cells were passed over a 40 μm sieve, washed with fresh media, and resuspended in 100 ml of fresh media and followed in a time course of 12 hours. Every hour, a sample was taken for western blotting and flow cytometry analysis. For western blotting, 5 ml of cells were collected with a 50 μm sieve and immediately frozen in liquid nitrogen. To extract proteins, samples were crushed with a small pistil in an Eppendorf tube and mixed with 200–250 μl of 1 × loading buffer in 1 × PBS. Samples were boiled for 5 min at 99°C followed by centrifugation at full speed for 5 min. Fifteen μl were loaded onto 15% SDS-PAGE gels and analyzed by western blotting.

For the flow cytometry analysis, 2 ml of cells were collected with a 50 μm sieve. The cells were resuspended in a small Petri dish in 400 μl of extraction buffer (CyStain UV Precise P kit from Sysmex) and chopped with a razor blade. The sample was transferred onto a 50 μm CellTrics Disposable filter (Partec), placed on top of a flow cytometry tube, and 1,5 ml of DAPI stain solution (CyStain UV Precise P kit from Sysmex) was added to the sieve. The samples were then analyzed by the Partec flow cytometer with a gain set to 380.

### Staining of *Arabidopsis* roots with EdU

For the whole mount EdU staining and immunostaining with the phosphorylation specific antibody in roots, the protocol for the Click-iT EdU imaging kit from Invitrogen was combined with the protocol from [73]. *Arabidopsis* plants were grown on MS plates for 1 week and fifteen seedlings were incubated for 1 hour in liquid MS media containing 10 μM EdU at room temperature. The seedlings were washed twice with MS media to remove the EdU. The roots were cut off and transferred into an Eppendorf tube with fixative solution, incubated for 1 hour at room temperature, and washed twice for 10 min with 1×PBS and twice for 5 min with water. The roots were transferred to the microscope slide and allowed to dry. The material was then rehydrated by adding 1 × PBS for 5 min followed by incubation with 2% driselase (Sigma) for 60 min at 37°C in a moisture chamber to remove the cell wall. The roots were washed 6 times for 5 min with 1×PBS at room temperature. Mixture of 3% IGEPAL CA-630 plus 10% DMSO was added to the slide and incubated for 60 min at room temperature. The solution was removed, and the slides were washed 8 times with 1 × PBS for 5 min. The slides were then incubated with 3% BSA in 1 × PBS for 5 min before revealing EdU incorporation with the Click-iT reaction buffer. After this reaction, the slides were washed once for 5 min with 3% BSA in 1 × PBS followed by blocking for 60 min at room temperature with 3% BSA in 1 × PBS. Approximately 150 μl of the primary antibody diluted 1:100 in 3% BSA 1 × PBS was added to the slides and incubated for 4 hours at 37°C in a humid chamber and then overnight at 4°C. Before incubating with the secondary antibody, the slides were washed 5 times with 1 × PBS for 10 min at room temperature. Alexa flour 555 labelled goat anti-rabbit IgG (Life Technologies) diluted 1:200 in 1 × PBS containing 3% BSA was added to slides and incubated for 3 hours at 37°C in a humid chamber. Finally, slides were again washed 5 times for 10 min with 1 × PBS at room temperature and mounted in Vectashield (Vector Laboratories) with 1 μg/ml DAPI. Slides were examined on an LSC confocal microscope (Carl Zeiss) and confocal sections were acquired with a 40 × oil objective, exported to Adobe Photoshop, and prepared for publication.

### Treatment of *Arabidopsis* cell suspension with cell cycle inhibitors

The T87 cell suspension was grown on a shaker at 130 rpm under constant light. For treatment with different cell cycle inhibitors, 5 ml of the 7-day old culture were mixed with 5 ml of fresh BY-2 media supplemented with the indicated amounts of the inhibitors. The concentrations that were used for the treatment were: 50 μM roscovitine (50 mM solution from Merck); 10 mM hydroxyurea (SigmaAldrich); 4 μg/ml aphidicolin (SigmaAldrich). Cells were incubated with the inhibitors for 24 hours and subsequently collected by removing the media with a 50 μm mesh, shock frozen in liquid nitrogen, and stored at -80°C.

For treatment of cell suspension cultures with different concentrations of roscovitine, a 7-day old *Arabidopsis* T87 culture was diluted in BY-2 media to an OD_600_ of 0.167. 10 ml of the diluted culture were incubated for 24 hours with or without 10 μM, 20 μM, 30 μM, 40 μM, and 50 μM roscovitine with shaking under constant light. Seven ml of each sample were collected, and the cells were disrupted with ceramic beads and immediately mixed with 1 × Laemmli loading buffer in 0.3 × PBS without prior enrichment of nuclei.

### Immunoprecipitation of CDKA;1 from *cdka;1-/-* CDKA;1::YFP plants

For immunoprecipitation, 1.5 g of 15 day old CDKA;1::YFP *cdka;1-/-* [68] and WT seedlings grown on MS plates were crushed in liquid nitrogen and powder was resuspended in PEB400 buffer (50 mM HEPES-KOH pH 7.9, 400 mM KCl, 1 mM DTT, 2.5 mM MgCl_2_, 1 mM EDTA, 0.1% Triton X-100) [74] supplemented with protease inhibitor cocktail (Roche) (100 μl of buffer per 100 mg of seedlings) and the suspension was incubated on ice for 10 minutes. The samples were centrifuged for 10 min at full speed at 4°C. The supernatants were transferred to a new tube and centrifuged as above. The volume of the sample was measured and the same volume of PEB buffer without KCl was added to obtain the PEB buffer with 200 mM KCl. At this step, an input aliquot was taken and mixed with 5 × loading buffer. Agarose beads coupled with GFP nanobodies (40 μl; VBC Molecular Biology Services) were washed twice with PEB200 and the protein extracts were added to the beads and the mixture was incubated overnight at 4°C on a rotating wheel. The beads were washed 3 times with 1 ml of PEB200 followed by 2 washes with kinase buffer (20 mM Tris-HCl pH7.5, 50 mM KCl, 5 mM MgCl_2_, 1 mM DTT) if they were used for the *in vitro* kinase assay. To analyze immunoprecipitations by SDS-PAGE and western blotting, 30 μl of 1 × loading buffer in PEB200 were added to the beads. After boiling for 5 min, the supernatants were loaded onto a 12% SDS-PAGE gel.

### *In vitro* phosphorylation assay

For the kinase assay, 10 μl of kinase buffer were added to the agarose beads with the immunoprecipitated CDKA;1-YFP. One μg of the reconstituted histone dimers (H2A.W.6-H2B.9, H2A.W.7-H2B.9, H2A.13-H2B.9) and 200 μM ATP were added and the reaction was mixed before incubating for 35 min at 30°C. As a control, 20 μl kinase buffer were mixed with the dimers and ATP for 35 min at 30°C. Reactions were stopped by adding 5× loading buffer and analyzed by SDS-PAGE followed by western blotting with P-KSPK antibody.

### Cloning of H2A.W.7 into overexpression vector pET15b

cDNA encoding H2A.W.7 was PCR amplified from a seedling cDNA library using the following primers: W7pET15bfor (GCATCCATATGGAGTCATCACAA) and W7pET15brev (CTAATGGATCCTCAAGCCTTCTT). The PCR product was cleaned using the PCR purification kit from Qiagen, digested with *Nde*I/*Bam*HI, gel purified, and ligated into *Nde*I/*Bam*HI opened pET15b (Novagene). Plasmids for expression of His-tagged H2A.W.6, H2A.13, and H2B.9 have been published [20,22].

### Overexpression and purification of recombinant histones and assembly of H2A-H2B dimers

Proteins were overexpressed in *E*. *coli* BL21 (DE3) overnight at 37°C. Histone purification was performed as previously described [75,76]. Cells pellets were resuspended in sonication buffer 1 (50 mM Tris-HCl pH8.0, 500 mM NaCl, 1 mM PMSF, 5% glycerol) and sonicated with 50% power for 5 min. After centrifugation at 15,000 rpm at 4°C for 20 min, pellets were resuspended in sonication buffer 1 and sonicated at 35% power for 2 min followed by centrifugation as before. The resulting pellets were resuspended in sonication buffer 2 (50 mM Tris-HCl pH 8.0, 500 mM NaCl, 7 M guanidine hydrochloride, 5% glycerol) and sonicated as described before. After the third sonication, the suspension was rotated at 4°C overnight. After centrifugation, the supernatants containing denatured proteins were mixed with Ni-NTA resin (Qiagen) and incubated for 60 min at 4°C. To remove the supernatant, the suspension was centrifuged. The resin was resuspended in wash buffer (50 mM Tris-HCl pH8.0, 500 mM NaCl, 6 M urea, 5% glycerol, 5 mM imidazole) and transferred into an Econo-column (BioRad). After washing with 50 column volumes of wash buffer, proteins were eluted with elution buffer (50 mM Tris-HCl pH8.0, 500 mM NaCl, 6 M urea, 5% glycerol, 500 mM imidazole) and collected in 2 ml fractions. The fractions were analyzed on a 15% SDS-PAGE gel and those containing histones were pooled together and dialyzed against 4L of 10 mM Tris-HCl pH 7.5, 2 mM 2-mercaptoethanol at 4°C for 2 days. After checking the thrombin cleavage efficiency with different U/mg of thrombin, the estimated amount of thrombin was added to each sample and incubated for 3 hours at room temperature followed by analysis on a 15% SDS-PAGE gel. Proteins were further purified by cation ion exchange chromatography. The sample was applied to an SP sepharose column (GE Healthcare) connected to an Äkta system. The column was equilibrated and washed with equilibration buffer (20 mM CH_3_COONa pH5.2, 200 mM NaCl, 6 M urea, 5 mM 2-mercaptoethanol, 1 mM EDTA). For elution, a linear gradient of 200–800 mM NaCl in elution buffer (20 mM CH_3_COONa pH 5.2, 6 M urea, 5 mM ß-mercaptoethanol, 1 mM EDTA) was used. The fractions were again analyzed on a 15% SDS-PAGE gel. Histone containing fractions were pooled together and dialyzed against 4 L of 2 mM 2-mercaptoethanol 4 times for 4 hours. Finally, the purified histones were freeze-dried.

For histone H2A-H2B dimer reconstitution, freeze-dried histones were resolved in unfolding buffer (20 mM Tris-HCl pH 7.5, 7 M guanidine hydrochloride, 20 mM 2-mercaptoethanol) at a concentration of 1 mg/ml at a 1:1 molar ratio and incubated for 2 hours at 4°C on a wheel. Samples were step-wise dialyzed against refolding buffer (20 mM, Tris-HCl pH 7.5, 1 mM EDTA, 0.1 M PMSF, 5% glycerol, 5 mM 2-mercaptoethanol) staring with 2 M NaCl at 4°C overnight, followed by 1 M NaCl refolding buffer at 4°C for 4 hours, 0.5 M NaCl refolding buffer at 4°C for 4 hours, and finally against 0.1 M NaCl refolding buffer at 4°C overnight. Proteins were concentrated by centrifugation with a 10 kDa cut-off membrane (Merck Millipore) to a volume of 300 μl. The sample was applied to a Superdex200 gel filtration column in 0.1 M NaCl refolding buffer. Peak fractions were analyzed on a 15% SDS-PAGE gel and fractions containing the heterodimers were pooled together. The heterodimers were then concentrated by ultrafiltration (10 kDa cut-off) and, at the same time, the buffer was exchanged to kinase buffer. The final protein concentration was determined by measuring the absorbance at 280 nm and the quality was analyzed on a 15% SDS-PAGE gel.

### Schizosaccharomyces pombe strains and media

Unless otherwise stated, cells were grown in rich medium (YES). Gene replacement and tagging were performed by homologous recombination using a plasmid-based method [77]. The primers used are listed in S1 Table. In brief, pCloneNat1 and pCloneHyg1 were used to fuse the H2A.W.6 (*At*) C-terminal 21 amino acids to the endogenous C-terminus of *Sp*H2A.α and *Sp*H2A.β by using the natMX4 or hphMX4 cassette, respectively [23]. All constructed strains in this study were verified by PCR analysis and sequencing.

### *S*. *pombe* chromatin fractionation assay

Chromatin fractionation of WT and *Sp*H2A.W^*At*^ strains was performed as previously described [78] and fractions were analyzed by western blotting using anti-αtubulin (T6199, Sigma), anti-H3 (ab1791, Abcam), and anti-H2A.W.6 (antibodies recognizing C-terminal KSPK motif).

### Preparation of *S*. *pombe* whole cell extracts and acid extraction of histones for MS analysis

Cells were disrupted by 0.5 mm glass beads in lysis buffer (50 mM Tris-HCl pH 7.5, 150 mM NaCl, 5 mM EDTA, 10% glycerol and 1 mM PMSF) and centrifuged at 14,000 × *g* for 15 min at 4°C. Supernatant was collected as a whole cell extract. Histones were analyzed by western blotting with antibodies against fission yeast-specific phosphoS129 of H2A (ab17353, Abcam), P-KSPK, and H3 (ab1791, Abcam). For mass spectrometry, histones from WT, *Sp*H2ACT, *Sp*H2ACT-AA, *Sp*H2A.W^*At*^, and *Sp*H2A.W4A^*At*^ cells were prepared by the acid extraction and acetone precipitation method [79].

### Indirect immunofluorescence on *S*. *pombe* cells

For detection of *Sp*H2A.W^*At*^ by immunofluorescence, cells expressing FLAG-tagged *Sp*H2A.W^*At*^ were fixed with 4% paraformaldehyde, digested to spheroplasts with zymolyase (Zymo Research), permeabilized with 1% Triton X-100, and incubated with an anti-Flag antibody (F1804, Sigma) as the primary antibody at a 1:100 dilution and anti-mouse IgG Alexa Fluor 488 as the secondary antibody (A11029, Life Technologies) at a 1:100 dilution. Cells were placed onto poly L-lysine-coated coverslips and DAPI stained. Microscopic analysis was performed using LSM700 laser scanning confocal microscope (Zeiss).

### Yeast spot assay

Cells were grown in YES medium at 32°C until the exponential phase and for all indicated strains the OD_600_ was adjusted to 1.0. Five-fold serial dilutions were made with fresh YES and 2 μl were spotted on YES plates or YES plates containing 100 ng/μl Bleomycin, 0.004% methyl methane sulfonate (MMS), 20 μM Camptothecin (CPT) and incubated at 32°C for 3 days.

### Peptide pull down assay

6His-tagged Mdb1 recombinant protein was expressed in BL21 and purified using Ni-NTA beads (Qiagen) following manufacturer’s instructions. As control, we synthesized two peptides corresponding to residues 120–132 of yeast H2A C-terminal tail, with serine 129 being unphosphorylated (termed as peptide 1) and phosphorylated (termed as peptide 2). The other two peptides corresponding to residues 129–149 of H2A.W6 from *Arabidopsis*, with serine 145 being unphosphorylated (termed as peptide 3) and phosphorylated (termed as peptide 4) together with SpQ peptide. All these peptides coupled with biotin at N-terminal. Twenty micrograms of each peptide were incubated with 20 μl of pre-washed Dynabeads M-280 Streptavidin (Invitrogen) at RT for 1 hour, then incubated with purified Mdb1 in peptide binding buffer (50 mM Tris-HCl, pH 7.5, 100 mM NaCl, 0.05% NP-40) at 4°C for overnight. Beads were washed with peptide binding buffer and eluted with SDS loading buffer.

### Yeast genetic interaction screening

Large-scale crosses by SGA (synthetic genetic array) were carried out using the Bioneer haploid deletion mutant library (version 3.0) and the query strains WT and *sp*H2A.W^*At*^. Manipulations were performed using a Singer RoToR colony pinning robot, essentially as described previously [80]. First, the library and query strains were arrayed in 384 colony format on YES agar containing 250 μg/ml G418 (Geneticin; Life Technologies, 10131027) or 100 μg/ml ClonNat (Nourseothricin; Jena Bioscience, AB-102XL) and 50 μg/ml Hygromycin B (Invitrogen/Life Technologies, 10687010), respectively. Mating between library and query strains, and selection of progeny was largely performed as previously reported [81]. Briefly, query and library stain cells were mixed in a drop of sterile H_2_O on SPAS plates and allowed to sporulate at 24°C for 3 days. The resulting cell/spore mixture was incubated at 42°C for 7 days to enrich for spores and plated onto selective media to select for haploid progeny bearing the gene deletion and/or *sp*H2A.W^*At*^. Finally, the selected haploid progeny was grown YES or YES supplemented with or 9 mM hydroxyurea (Sigma, H8627) at 32°C. For each individual screen with the WT and *sp*H2A.W^*At*^ query strains, technical duplicates were processed following the germination step, and each screen was repeated independently at least 3 times, resulting in a total number of n = 6 screen for data analysis.

### Data analysis of the genetic screen and visualization

SGA analysis was performed as previously described [82], with some modifications. Genetic interactions were assessed by colony growth on YES plates containing no additives (non-selective media; N/S) and YES plates containing formamide or hydroxyurea (treatment) and quantified by determining colony sizes (area) of digitalized pictures. As colonies at the edges of the plates show increased growth due to better availability of nutrients, the size of colonies of the first and second outer most rims was corrected by multiplying with a correction factor. For calculating this factor, we determined the ratio between the median of all colonies in the middle of the plate (i.e., excluding the first and second rims) and the median of the first and second outer most rims. Next, the sensitivity towards stress conditions was determined by calculating the ratio between growth on ‘treatment’ and ‘N/S’ for each individual mutant; the obtained value for each mutant was then normalized to the median of the treatment: N/S values derived from all mutants of the same 384-plate. Finally, the entire dataset of each screen was then log_2_-transformed and median-normalized to all mutants from the entire screen (array). To select for robust genetic interactions, data from all screens, query strains and stress conditions were filtered to select for those mutants containing at least 80% of present values and show at least 5 absolute values higher or equal to 0.3 (log_2_), resulting in the selection of 529 mutants. Next, hierarchical clustering of the genetic interaction dataset was performed using Euclidean distance as the similarity metric and complete linkage as the clustering method using Gene Cluster 3.0. Hierarchical cluster maps were visualized with Java TreeView (version 1.1.6) with negative and positive values represented in blue and yellow, respectively (grey: missing or N/A data).

## Supporting information

S1 Fig Mass spectrometry analysis of H2A.W.6 and H2A.W.7 modifications. (A) Summary of all modifications detected in the N- and C- tails of H2A.W variants with samples from leaves and flowers used to create Fig 1C. (B) Numbers of spectra covering S145 and S129 and those containing phosphorylated S145 and S129 in all MS data sets for H2A.W.6 and H2A.W.7 presented in Fig 1C. Majority of lysine residues were also modified by formylation. However, we did not include this into our analysis because this modification could also be an artifact of silver staining procedure that includes formaldehyde.
(TIF)

S2 Fig Antibody against the phosphorylated serine 145 of H2A.W.6 is specific but recognizes the phosphorylated KSPK motif of H2A.W.7. (A) Affinity purified antibody obtained after immunization of rabbits with the CEEKATKSPVKSpPKKA-OH peptide were tested on dot blots with the serially diluted peptides indicated at the top. Note that unphosphorylated peptide or peptide phosphorylated at serine 141 do not cross-react with the affinity purified antibody. (B) The same purified antibody was tested against C-terminal peptides in the unphosphorylated and phosphorylated forms from H2A.W.6, H2A.W.7, and H2A.W.12 as above. Note that the H2A.W.7 but not the H2A.W.12 peptide cross reacts with the P-KSPK antibody, indicating that the epitope recognized includes the C-terminal alanine that is absent in H2A.W.12.
(TIF)

S3 Fig Phosphorylation of the KSPK motif changes during the cell cycle. (A) Protein sequence alignment of tobacco H2A.W isoforms with *Arabidopsis* H2A.W.6. Only the C-terminal tail is shown, with the conserved KSPK motif indicated in a red box. (B) Protein extracts from BY-2 cell suspension culture were analysed by western blotting with the anti-P-KSPK antibody. Colorimetric and chemiluminescence images were overlaid using the ChemiDoc software to align the position of the signal with the protein ladder. (C) Flow cytometry profiles of tobacco BY-2 cell after aphidicolin block and release. Cells were analyzed in a time course as in Fig 3A. BY-2 suspension culture was blocked with 20 μg/ml aphidicolin for 24 hours. Upon removal of the aphidicolin block, cells proceeded through the cell cycle in a synchronized manner that was followed by flow cytometry during a twelve-hour time course. (D) Control BY-2 cell suspension culture treated with 1% DMSO for 24 hours and then followed over a twelve-hour time course by flow cytometry. Only profiles for time points T0, T9, and T12 are shown. (E) Protein extracts from DMSO treated BY-2 cells were analyzed by western blotting with the indicated antibodies. Note that there is no fluctuation of phosphorylated H2A.W.6 (top panel) in unsynchronized cells compared to those synchronized by aphidicolin (see Fig 3A).
(TIF)

S4 Fig Phosphorylation of the H2A.W.6 KSPK motif and expression of H2A.W.6 are linked to CDK activity in *Arabidopsis*. (A) Root tips of WT plants were immunostained with the H2A.W.6 antibody. Note that all nuclei display H2A.W.6 signal. Single confocal sections of two root tips are shown. (B) One-week old *Arabidopsis* cell suspension culture was treated for 24 hours with the indicated concentrations of roscovitine, a potent inhibitor of cell cycle-dependent kinases. Protein extracts were analyzed by western blotting with H2A.W.6 and P-KSPK specific antibodies.
(TIF)

S5 Fig Analysis of KSPK phosphorylation *in planta* and in *S*. *pombe*. (A) Analysis of γH2A.X, γH2A.W.7, and P-KSPK in transgenic seedlings expressing H2A.W.7 hypomorphic mutants in the SQ motif after treatment with 20 μg/ml of bleomycin for 2 hours. Note that bleomycin treatment does not induce phosphorylation of the KSPK motif on either WT H2A.W.7 or the AQ and DQ mutants of H2A.W.7. This band would be shifted above endogenous H2A.W.6 due to the presence of the HA tag. (B) Schematic diagrams of two *S*. *pombe* H2A variants and *Arabidopsis* H2A.W.6 with the indicated C-terminal tail sequences used for construction of the mosaic H2A variants depicted in Fig 4A. (C) Analysis of *Sp*H2A.W^*At*^ association with the chromatin. Whole cell, chromatin free, and chromatin bound fractions from *Sp*H2A and *Sp*H2A.W^*At*^ strains were analyzed by western blotting with the indicated antibodies. Tubulin and histone H3 were used as cytoplasmic and nuclear controls, respectively. (D) MS analysis of *Sp*H2A SQ motif phosphorylation in fission yeast strains expressing the indicated mosaic or mutated H2A variants. Highly similar levels, quantified as peak areas, of peptides covering phosphorylated SQ motif were measured in all strains. (E-F) Phosphorylation of *Sp*H2A.W^*At*^ at the KSPK motif confers sensitivity to DNA damage. Serial dilutions (5×) of fission yeast cells expressing WT and indicated *Sp*H2A mosaic variants were spotted onto either YES or YES plates containing 0.004% methyl methane sulfonate (MMS) and 20 μM camptothecin (CPT) and incubated at 32°C for 3 days.
(TIF)

S6 Fig Cluster analysis of the SGA screen for deletion mutant that suppress growth of *Sp*H2A.W^*At*^ on 8 mM HU plates. For each experiment, four replicates were performed. Clustering analysis showing different group of genes with sensitivity to hydroxyurea (HU), either in the single or the double mutant with *Sp*H2A.W^*At*^. Blue indicates synthetic interaction, yellow indicates suppressive interaction, black indicates no interaction, and gray indicates the absence of data.
(TIF)

S7 Fig H2A.W.7 variants occurred several times during evolution of angiosperms. (A) Phylogenetic three of H2A.W variants. All H2A.W.7 variants (those containing SQ motif in the C-terminal tail) are indicated. H2A.W sequences (396) from 105 different species (S1 Text) were obtained by BLAST search of genomes on the Phytozome (https://phytozome.jgi.doe.gov/pz/portal.html). Retrieved sequences were aligned with ClustalW, manually inspected, and sequences with insertions or deletions in the core histone domain and with long extensions at the N- and C-termini were removed. The three was constructed by the Neighbour-Joining method with Jukes-Cantor protein distance measure and 1,000 bootstraps with CLC Genomics Workbench 11.0. All sequences used for the alignment can be found in the S1 Text. (B) Sequence alignment of C-terminal tails from all H2A.W variants containing an SQ motif (red). Conserved KSPKK motifs are indicated in blue.
(TIF)

S1 Table List of primers used in this study. (XLSX)

S1 Text Sequences of H2A.W variants used to create phylogenetic three in S7 Fig. (DOCX)
