# Supplementary figures and images for: Crosstalk between H2A variant-specific modifications impacts vital cell functions
Source: PLoS Genet. 2021 Jun 4;17(6):e1009601. doi: 10.1371/journal.pgen.1009601 (PMC8208582; doi:10.1371/journal.pgen.1009601)

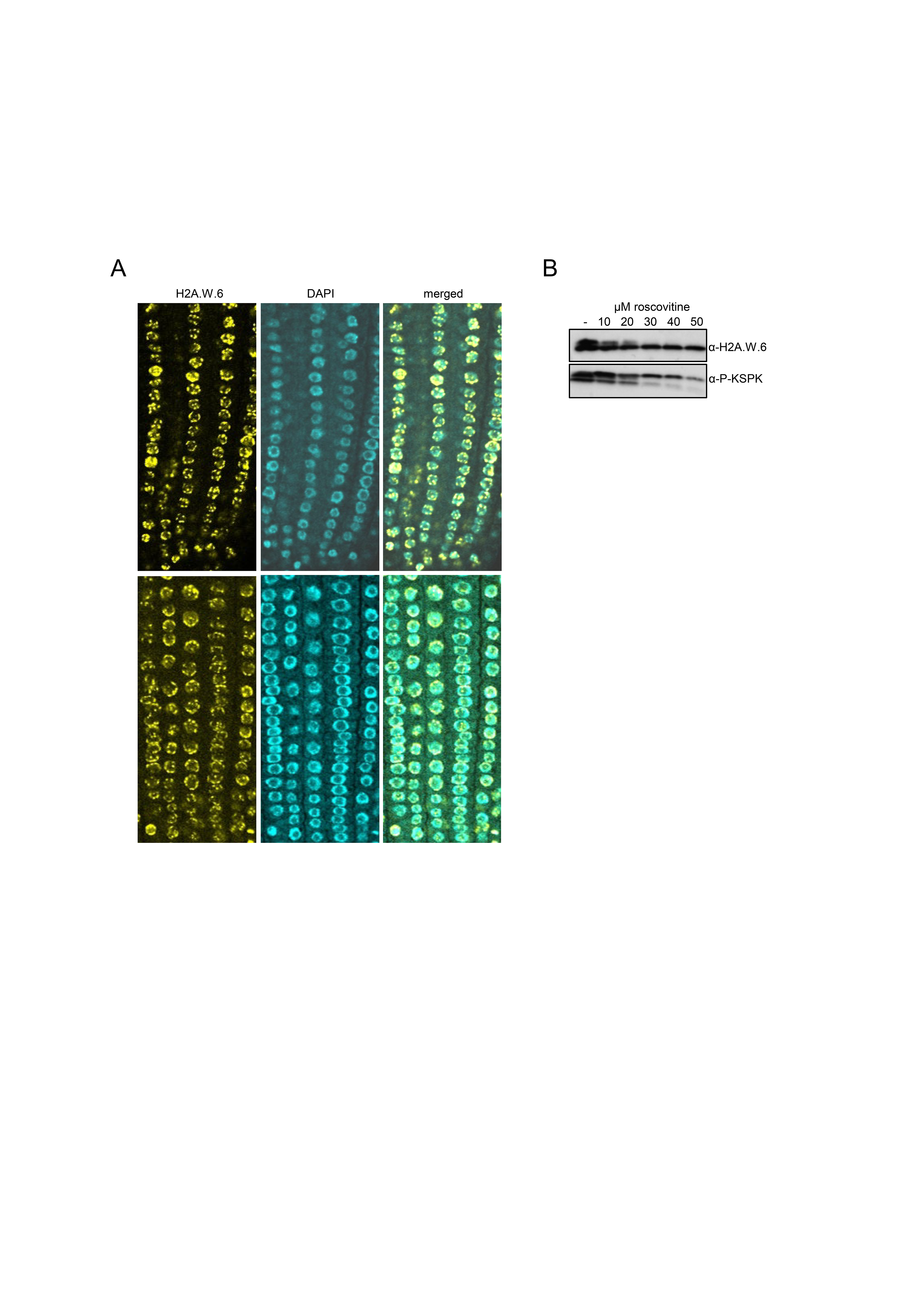

Supplement: S4 Fig — (A) Root tips of WT plants were immunostained with the H2A.W.6 antibody. Note that all nuclei display H2A.W.6 signal. Single confocal sections of two root tips are shown. (B) One-week old Arabidopsis cell suspension culture was treated for 24 hours with the indicated concentrations of roscovitine, a potent inhibitor of cell cycle-dependent kinases. Protein extracts were analyzed by western blotting with H2A.W.6 and P-KSPK specific antibodies. (TIF) [file pgen.1009601.s004.tif]

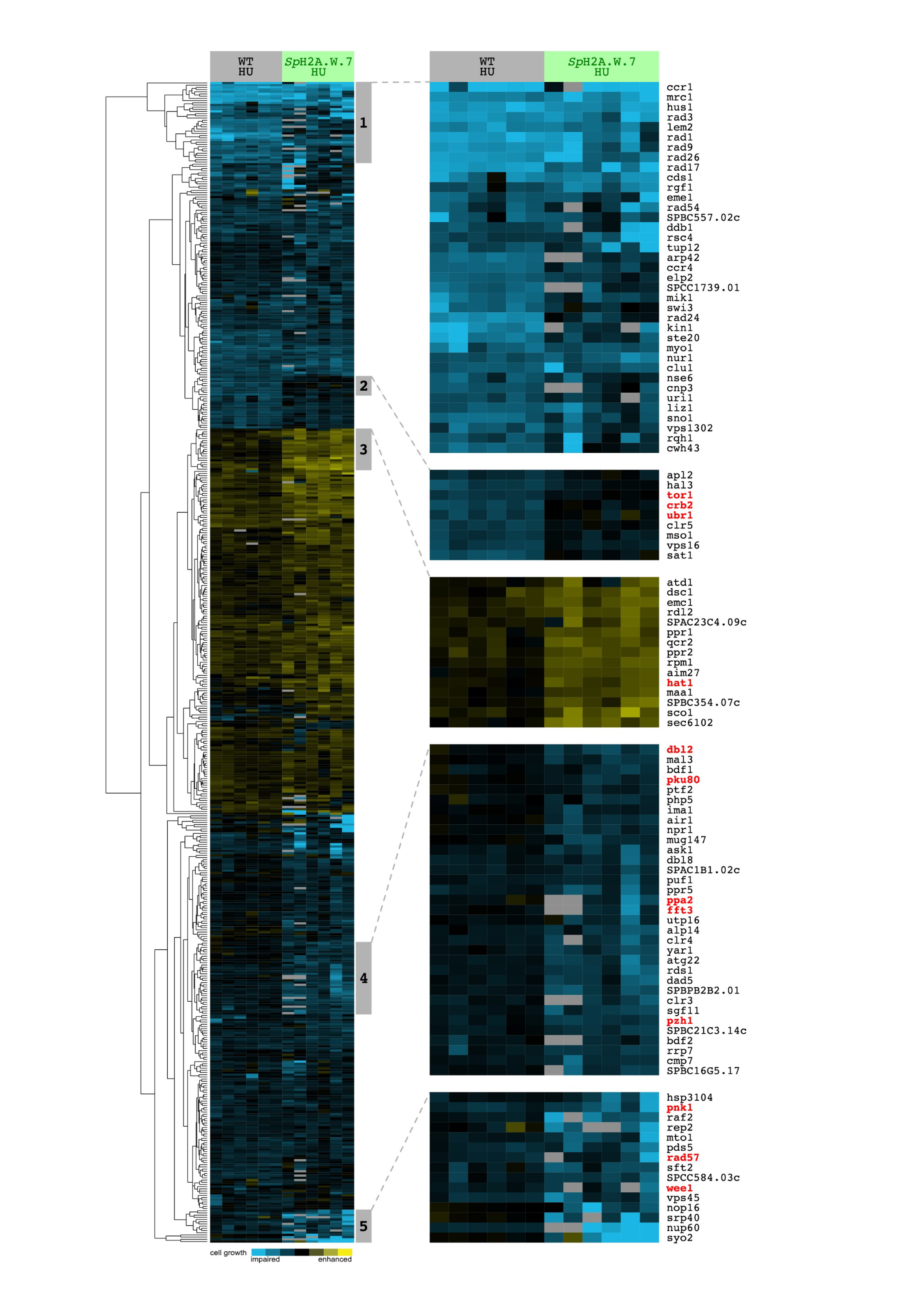

Supplement: S6 Fig — For each experiment, four replicates were performed. Clustering analysis showing different group of genes with sensitivity to hydroxyurea (HU), either in the single or the double mutant with SpH2A.WAt. Blue indicates synthetic interaction, yellow indicates suppressive interaction, black indicates no interaction, and gray indicates the absence of data. (TIF) [file pgen.1009601.s006.tif]
