# Supplementary material for: Crosstalk between H2A variant-specific modifications impacts vital cell functions
Source: PLoS Genet. 2021 Jun 4;17(6):e1009601. doi: 10.1371/journal.pgen.1009601 (PMC8208582; doi:10.1371/journal.pgen.1009601)
Supplement: S1 Text — (DOCX) [file pgen.1009601.s009.docx]

**S1 Text. Sequences of H2A.W variants used to create phylogenetic three in S7 Fig.**

**Brassicaceae**

>Ahalleri_v1.1|Araha.2561s0001|Araha.2561s0001.1

METTAKVKKAFGGRKPGGARTKSVSKSIKAGLQFPVGRITRFLKKGRYAQRLGGGAPVYM

AAVLEYLAAEVLELAGNAARDNKKTRIIPRHLLLAIRNDEELGKLLSGVTIAHGGVLPNI

NSVLLPKKTASKSTEEKATKSPAKSPKKA*

>Ahalleri_v1.1|Araha.15345s0004|Araha.15345s0004.1

MESSQATTKPARGAGGRKGGDRKKSVTKSVKAGLQFPVGRIARYLKKGRYAIRYGSGAPV

YLAAVLEYLAAEVLELAGNAARDNKKNRINPRHLCLAIRNDEELGKLLHGVTIASGGVLP

NINPVLLPKKSTAAEKASPATKSPKKA*

>BrapaFPsc_v1.3|Brara.B00055|Brara.B00055.1

MDSGTKVKKGAGGRRGGGPKKKPVSRSVKAGLQFPVGRIGRYLKKGRYSKRVGTGAPVYL

AAVLEYLAAEVLELAGNAARDNKKNRIIPRHVLLAVRNDDELGKLLKGVTIAHGGVLPNI

NPVLLPKKSEKAASTTKVTKSPSKATKSPKKA*

>BrapaFPsc_W7

MESSAAATKPARGGRRGGDRKKSVSKSVKAGLQFPVGRIARYLKKGRYAVRYGSGAPVYL

AAVLEYLAAEVLELAGNAARDNKKNRINPRHLCLAIRNDEELGKLLHGVTIASGGVLPNI

NPVLLPKRTAGSSQGEKVKAEKVKASSPAKKLSPKKG*

>BrapaFPsc_v1.3|Brara.J01356|Brara.J01356.1

METTGKVKKAFGGRKAGGPKTKSVSKSIKAGLQFPVGRITRFLKKGRYAQRLGGGAPVYM

AAVLEYLAAEVLELAGNAARDNKKSRIIPRHLLLAIRNDEELGKLLSGVTIAHGGVLPNI

NSVLLPKKSAKSSEDAAPKSPGKSPKKA*

>BrapaFPsc_v1.3|Brara.C01065|Brara.C01065.1

METTGKVKKAFGGRKAGGPKTKSVSKSIKAGLQFPVGRITRFLKKGRYAQRLGGGAPVYM

AAVLEYLAAEVLELAGNAARDNKKSRIIPRHLLLAIRNDEELGKLLSGVTIAHGGVLPNI

NSVLLPKKSAKSTEEVASKSPAKSPKKA*

>BrapaFPsc_W7

MESSSPAAAAKPARGAGGRKGGDRKKSVSKSAKAGLQFPVGRISRYLKKGRYAIRYGAGA

PVYLAAVLEYLAAEVLELAGNAARDNKKNRINPRHLCLAIRNDEELGKLLHGVTIASGGV

LPNINPVLLPKRSASQTEKPEKAAKAAKSPKKA*

>Alyrata_v2.1|AL6G39360|AL6G39360.t1

MESSQATTKPARGAGGRKGGDRKKSVTKSVKAGLQFPVGRIARYLKKGRYAIRYGSGAPV

YLAAVLEYLAAEVLELAGNAARDNKKNRINPRHLCLAIRNDEELGKLLHGVTIASGGVLP

NINPVLLPKKSTAAEKASPATKSPKKA*

>Alyrata_v2.1|AL8G36430|AL8G36430.t1

METTAKVKKAFGGRKPGGAKTKSVSKSIKAGLQFPVGRITRFLKKGRYAQRLGGGAPVYM

AAVLEYLAAEVLELAGNAARDNKKSRIIPRHLLLAIRNDEELGKLLSGVTIAHGGVLPNI

NSVLLPKKTASKSTEEKASKSPVKSPKKA*

>Boleraceacapitata_v1.0|Bol025908|Bol025908

METTGKVKKAFGGRKAGGPKTKSVSKSIKAGLQFPVGRITRFLRKGRYAQRLGGGAPVYM

AAVLEYLAAEVLELAGNAARDNKKSRIIPRHLLLAIRNDEELGKLLSGVTIAHGGVLPNI

NSVLLPKKSAKSTEEVASKSPAKSPKKA*

>Boleraceacapitata_v1.0|Bol032415|Bol032415

MESPAAAAAAKPARGGAGGRKGGDRKKSVTKSVKAGLQFPVGRISRYLKKGRYAVRYGAG

APVYLAAVLEYLAAEVLELAGNAARDNKKNRINPRHLCLAIRNDEELGKLLHGVTISSGG

VLPNINPVLLPKRAAGSEKAAAKSPKKA*

>Boleraceacapitata_v1.0|Bol012773|Bol012773

MESSPPATKAARGGRRGGDRKKSVSKSVKAGLQFPVGRIARYLKKGRYAIRYGSGAPVYL

AAVLEYLAAEVLELAGNAARDNKKNRINPRHLCLAIRNDEELGKLLHGVTIASGGVLPNI

NPVLLPKRTTGSSHGEKEKASSPAKKSPKKG*

>Camplexicaulis_W7

MESPAAAKPARGAGGRRGGDRKKSVSKSVKAGLQFPVGRISRYLKKGRYAIRYGSGAPVY

LAAVLEYLAAEVLELAGNAARDNKKNRINPRHLCLAIRNDEELGKLLHGVTIASGGVLPN

INPILLPKRAAGSSQAEKASPATKSPKKA*

>Camplexicaulis_v1.1|Caamp.1037s0778|Caamp.1037s0778.1

METTGKVKKGFGGRKGGGPKNKSVSKSIKAGLQFPVGRITRFLKKGRYAQRLGGGAPVYM

AAVLEYLAAEVLELAGNAARDNKKSRIIPRHLLLAIRNDEELGKLLSGVTIAHGGVLPNI

NSILLPKKSAKSTEESASKSPAKSPKKA*

>Camplexicaulis_v1.1|Caamp.1040s0418|Caamp.1040s0418.1

METTGKVKKGFGGRKGGGPKNKSVSRSIKAGLQFPVGRITRYLKKGRYAQRLGGGAPVYM

AAVLEYLAAEVLELAGNAARDNKKSRIIPRHLLLAIRNDEELGKLLSGVTIAHGGVLPNI

NSILLPKKSSKSTEETASKSPAKSPKKA*

>Alinifolium_W7

MESQAATKPARGGRRGGDRKKSVTKSVKAGLQFPVGRIARYLKKGRYALRYGSGASVYLA

AVLEYLAAEVLELAGNAARDNKKNRINPRHLCLAIRNDEELGKLLQGVTIASGGVLPNIN

PILLPKKSTASSSQTEKASPATKSPKKA*

>Alinifolium_v1.1|Alyli.0036s0278|Alyli.0036s0278.1

MESQAATKPARGGRRGGDRKKSVTKSVKAGLQFPVGRIARYLKRGRYALRYGSGASVYLA

AVLEYLAAEVLELAGNAARDNKKNRINPRHLCLAIRNDEELGKLLQGVTIASGGVLPNIN

PILLPKKSTASSSLTEKASPATKSPKKA*

>Rislandica_v1.1|Roisl.0046s0391|Roisl.0046s0391.1

MESAGAAATTKPTKGSGGRKGGDRKKSVSKSVKAGLQFPVGRISRYLKKGRYAVRYGAGA

PVYLAAVLEYLAAEVLELAGNAARDNKKNRINPRHLCLAIRNDDELGRLLNGVTIASGGV

LPNIHPVLLPKKPAASEKASSSASKSPKKA*

>Rislandica_v1.1|Roisl.0080s0280|Roisl.0080s0280.1

METAGKGKKGFGGRKAGGPKGKSVSKSIKAGLQFPVGRITRFLKKGRYAQRLGGGAPVYM

AAVLEYLAAEVLELAGNAARDNKKSRIIPRHLLLAIRNDEELGHLLSGVTIAHGGVLPNI

HSVLLPKKTAKSTEEKASKSPVKSPKKA*

>Rislandica_v1.1|Roisl.0034s0160|Roisl.0034s0160.1;Roisl.0034s0160.2

MDSGTKVKKGVAGRRGGGPKKKPVSRSVKSGLQFPVGRIGRYLKKGRYSKRVGTGAPVYL

AAVLEYLAAEVLELAGNAARDNKKNRIIPRHVLLAVRNDEELGKLLKGVTIAHGGVLPNI

NPILLPKKSEKAASTPKSPSKATKSPKKA*

>Itinctoria_v1.1|Isati.3580s0010|Isati.3580s0010.1

MEATGKVKKGFGGRKGGGPKSKSVSKSIKAGLQFPVGRITRYLKKGRYAQRLGGGAPVYM

AAVLEYLAAEVLELAGNAARDNKKSRIIPRHLLLAIRNDEELGKLLSGVTIAHGGVLPNI

NSVLLPKKSAKSTDESASKSPVKSPKKA*

>Itinctoria_v1.1|Isati.9602s0007|Isati.9602s0007.1

METTGKVKKGFGGRKGGGPKNKSVSKSIKAGLQFPVGRITRFLKKGRYAQRLGGGAPVYM

AAVLEYLAAEVLELAGNAARDNKKSRIIPRHLLLAIRNDEELGKLLSGVTIAHGGVLPNI

NSVLLPKKSAKSTEESASKSPVKSPKKA*

>Itinctoria_W7

MESPAAAKPARGAGGRRGGERKKSVSKSVKAGLQFPVGRISRYLKKGRYAIRYGSGAPVY

LAAVLEYLAAEVLELAGNAARDNKKNRINPRHLCLAIRNDEELGKLLHGVTIASGGVLPN

INPILLPKRAAGSSQAEKASPASKSPKKA*

>Lannua_W7

MESPAAKPTRGVGGRKGGERKKSVSKSLKAGLQFPVGRISRYMKKGRYALRYGSGAPVYL

AAVLEYLAAEVLELAGNAARDNKKNRINPRHLCLAIRNDEELGKLLHGVTIASGGVLPNI

NPVLLPKKSPASASQSEKASPATKSPKKA*

>Lannua_v1.1|Luann.0043s0196|Luann.0043s0196.1

MESQAAKPARGAGGRRGGDRKKSVSKSVKAGLQFPVGRIARFLKKGRYAIRYGSGAPVYL

AAVLEYLAAEVLELAGNAARDNKKNRINPRHLCLAIRNDEELGKLLHGVTIANGGVLPNI

NPVLLPKKSATSSSQAEKASPATKSPKKA*

>Mmaritima_W7

MESSPATTKPTRGAGGRKGGERKKSVTKSVKAGLQFPVGRIARYLKKGRYAIRYGSGAPV

YLAAVLEYLAAEVLELAGNAARDNKKNRINPRHLCLAIRNDEELGKLLSGVTIASGGVLP

NINPILLPKKSPAQAEKASSAAKSPKKA*

>Mmaritima_v1.1|Mamar.0064s0044|Mamar.0064s0044.1

METTTKVKKAFGGRKPGGTKTKSVSKSIKAGLQFPVGRITRYLKKGRYAQRLGGGAPVYM

AAVLEYLAAEVLELAGNAARDNKKTRIIPRHLLLAIRNDEELGKLLSGVTIAHGGVLPNI

NSILLPKKTATKSTEEKASKSPAKSPKKA*

>Salba_v1.1|Sialb.0198s0107|Sialb.0198s0107.1

METTGKVKKAFGGRKAGGPKTKSVSKSIKAGLQFPVGRITRFLKKGRYAQRLGGGAPVYM

AAVLEYLAAEVLELAGNAARDNKKSRIIPRHLLLAIRNDEELGKLLSGVTIAHGGVLPNI

NSVLLPKKSAKSSEEAAPKSPGKSPKKA*

>Salba_W7

MESPAAAAKPARGAGGRKGGDRKKSVSKSAKAGLQFPVGRISRYLKKGRYAIRYGAGAPV

YLAAVLEYLAAEVLELAGNAARDNKKNRINPRHLCLAIRNDEELGKLLHGVTIASGGVLP

NINPVLLPKRSASSSQTEKPEKAKATKSPKKA*

>Salba_W7

MEAPAAAATKPGRGGRRGGDRKKSVSKSVKAGLQFPVGRISRFLKKGRYAIRYGAGAPVY

LAAVLEYLAAEVLELAGNAARDNKKNRINPRHLCLAIRNDEELGKLLHGVTIASGGVLPN

INPVLLPKRAAAGSSQGEKEKASPAKKSPKKG*

>Cmaritima_W7

MESPAAAAAKPARGAGGRKGGDRKKSVSKSAKAGLQFPVGRISRYLKKGRYAIRYGAGAP

VYLAAVLEYLAAEVLELAGNAARDNKKNRINPRHLCLAIRNDEELGKLLAGVTIASGGVL

PNINPILLPKRSASQTEKTEKAKATKSPKKA*

>Cmaritima_v1.1|Camar.0059s0022|Camar.0059s0022.1

MESQAAPATKPARGGGRRGGDRKKSVSKSVKAGLQFPVGRIARYLKKGRYAIRYGSGAPV

YLAAVLEYLAAEVLELAGNAARDNKKNRINPRHLCLAIRNDEELGKLLRGVTIASGGVLP

NINPVLLPKRTAGSEKAKKSPKKA*

>Cmaritima_v1.1|Camar.1485s0005|Camar.1485s0005.1

MESPAAAKPARGAGGRKGGDRKKSVTKSVKAGLQFPVGRISRYLKKGRYAVRYGAGAPVY

LAAVLEYLAAEVLELAGNAARDNKKNRINPRHLCLAIRNDEELGKLLSGVTISSGGVLPN

INPVLLPKRAAGSEKAEKPEKATKSPKKA*

>Dsophioides_v1.1|Desop.0024s0039|Desop.0024s0039.1

MEATGKVKKAFGGRKAGGPKTKSVSKSIKAGLQFPVGRITRFLKKGRYAQRLGGGAPVYM

AAVLEYLAAEVLELAGNAARDNKKTRIIPRHLLLAIRNDEELGKLLSGVTIAHGGVLPNI

NSVLLPKKSAKSTEEKASKSPAKSPKKA*

>Dsophioides_v1.1|Desop.0094s0388|Desop.0094s0388.1

MDSGTKLKKGAAGRRGGGPKKKPVSRSVKSGLQFPVGRIGRYLKKGRYSKRVGTGAPVYL

AAVLEYLAAEVLELAGNAARDNKKNRIIPRHVLLAVRNDEELGKLLKGVTIAHGGVLPNI

NPILLPKKSEKAASNTRAPPKSPSKATKSPKKA*

>Dsophioides_v1.1|Desop.0216s0415|Desop.0216s0415.1

MESQAATKPARGGRRGGDRKKSVTKSVKAGLQFPVGRIARYLKRGRYALRYGSGASVYLA

AVLEYLAAEVLELAGNAARDNKKNRINPRHLCLAIRNDEELGKLLQGVTIASGGVLPNIN

PILLPKKSTASSSLTEKASPATKSPKKA*

>Esyriacum_v1.1|Eusyr.0014s0297|Eusyr.0014s0297.1

METTGKVKKGFGGRKAGGPKAKSVSKSIKAGLQFPVGRITRFLKKGRYAQRLGSGAPVYT

AAVLEYLAAEVLELAGNAARDNKKNRITPRHLLLAIRNDEELGKLLAGVTIAHGGVLPNI

NAVLLPKKSAKATEEKSPKSPGKSPKKA*

>Esyriacum_W7

MESSPAVAKPARGAGGRKGGERKKSVSKSAKAGLQFPVGRIARYLKKGRYAIRYGSGAPV

YLAAVLEYLAAEVLELAGNAARDNKKNRINPRHLCLAIRNDEELGKLLHGVTIASGGVLP

NINPILLPKKSAASSQSDKKASPATKSPKKG*

>Esyriacum_v1.1|Eusyr.0020s0132|Eusyr.0020s0132.1

MDSATKVKKGAAGRRGGGPKKKPVSRSVKSGLQFPVGRIGRYLKKGRYSKRVGTGAPVYL

AAVLEYLAAEVLELAGNAARDNKKNRITPRHVLLAVRNDEELGKLLKGVTIAHGGVLPNI

NPILLPKKSEKAASTPKKETKSPSKAATKSPKKVVTAT*

>Cviolacea_W7

METGGKVKKGAAGRKGGGPRSKPVARSVKAGLQFPVGRIGRYLKKGRYAQRVGSGAPVYL

AAVLEYLAAEVLELAGNAARDNKKNRIIPRHVLLAIRNDEELGKLLAGVTIAHGGVLPNI

HQVLLPKKSEKASQEPKTASKSPSKATKSPKKA*

>Cviolacea_v1.1|Clevi.0012s0253|Clevi.0012s0253.1

MESPAKPARGAGGRRGGDRKKSVSQSSKAGLQFPVGRIARYLKKGRYALRYGSGAPVYLA

AVLEYLAAEVLELAGNAARDNKKNRINPRHLCLAIRNDEELGKLLQGVTIASGGVLPNIN

PVLLPKKTGGGASSSETEKKSPATKSPRKS*

>Spinnata_W7

MESPAAAKPARGAGGRRGGDRKKSVSKSVKAGLQFPVGRISRYLKKGRYAIRYGSGAPVY

LAAVLEYLAAEVLELAGNAARDNKKNRINPRHLCLAIRNDEELGKLLHGVTIASGGVLPN

INPILLPKRAAGSSQAEKASPATKSPKKA*

>Spinnata_v1.1|Stapi.5085s0001|Stapi.5085s0001.1

METTGKVKKGFGGRKGGGPKNKSVSKSIKAGLQFPVGRITRFLKKGRYAQRLGGGAPVYM

AAVLEYLAAEVLELAGNAARDNKKSRIIPRHLLLAIRNDEELGKLLSGVTIAHGGVLPNI

NSVLLPKKSAKSTEEVASKSPAKSPKKA*

>Spinnata_v1.1|Stapi.2503s0007|Stapi.2503s0007.1

METTGKVKKGFGGRKGGGPKNKSVSKSIKAGLQFPVGRITRFLKKGRYAQRLGGGAPVYM

AAVLEYLAAEVLELAGNAARDNKKSRIIPRHLLLAIRNDEELGKLLSGVTIAHGGVLPNI

NSVLLPKKSSKSTEETVSKSPAKSPKKA*

>Evesicaria_v1.1|Eruve.6806s0004|Eruve.6806s0004.1

METTGKVKKAFGGRKPGGPKTKSVSKSTKAGLQFPVGRITRFLKKGRYAQRLGGGAPVYM

AAVLEYLAAEVLELAGNAARDNKKSRIIPRHLLLAIRNDEELGKLLSGVTIAHGGVLPNI

NSVLLPKKSAKSSEEAAPKSPGKSPKKA*

>Evesicaria_W7

MESPAAAAAATKPARGAGGRKGGDRKKSVSKSVKAGLQFPVGRISRYLKKGRYAIRYGAG

APVYLAAVLEYLAAEVLELAGNAARDNKKNRINPRHLCLAIRNDEELGKLLHGVTIASGG

VLPNINPILLPKRSASQTEKPEKAAKATKSPKKA*

>Evesicaria_v1.1|Eruve.1733s0016|Eruve.1733s0016.1

MESSPAAAKPARGAGGRKGGDRKKSVTKSVKAGLQFPVGRISRYLKKGRYAVRYGAGAPV

YLAAVLEYLAAEVLELAGNAARDNKKNRINPRHLCLAIRNDEELGKLLHGVTISSGGVLP

NINPVLLPKRAAGSEKGEKTEKAAKSPKKA*

>Chispanica_v1.1|Crahi.0222s0050|Crahi.0222s0050.1

MESPAAAKPARGAGGRKGGDRKKSVTKSVKAGLQFPVGRISRYLKKGRYAVRYGAGAPVY

LAAVLEYLAAEVLELAGNAARDNKKNRINPRHLCLAIRNDEELGKLLHGVTISSGGVLPN

INPVLLPKRAAASEKAEKPEKATKSPKKA*

>Chispanica_W7

MESPAAAKPARGAGGRKGGDRKKSVSKSAKAGLQFPVGRISRYLKKGRYAIRYGAGAPVY

LAAVLEYLAAEVLELAGNAARDNKKNRINPRHLCLAIRNDEELGKLLHGVTIASGGVLPN

INPVLLPKRSAASSQTEKPEKATKSPKKA*

>Chispanica_v1.1|Crahi.0055s0024|Crahi.0055s0024.1

METTGKVKKAFGGRKAGGPKTKSVSKSIKAGLQFPVGRITRFLKKGRYAQRLGGGAPVYM

AAVLEYLAAEVLELAGNAARDNKKSRIIPRHLLLAIRNDEELGKLLSGVTIAHGGVLPNI

NSVLLPKKSAKSSEEVASKSPAKSPKKA*

>Chispanica_W7

MESPAVTKPARGGRRGGDRKKSVSKSVKAGLQFPVGRIARYLKKGRYAIRYGAGAPVYLA

AVLEYLAAEVLELAGNASRDNKKNRINPRHLCLAIRNDEELGKLLHGVTIASGGVLPNIN

PVLLPKRAAGSSQGEKASPAKKSPKKG*

>Dstrictus_v1.1|Distr.0009s0128|Distr.0009s0128.1

MDSATKVKKGAAGRKGGGPKKKPVSRSVKSGLQFPVGRIGRYLKKGRYSKRVGTGAPVYL

AAVLEYLAAEVLELAGNAARDNKKNRITPRHVLLAVRNDEELGKLLKGVTIAHGGVLPNI

NPILLPKKYAEKAASTPKKEPKTATKSPKKAPAAA*

>Dstrictus_v1.1|Distr.0265s0378|Distr.0265s0378.1

MESTGKVKKGFGGRKAGGPKAKSVSKSIKAGLQFPVGRITRFLRKGRYAQRLGSGAPVYT

AAVLEYLAAEVLELAGNAARDNKKNRITPRHLLLAIRNDEELGKLLSGVTIAHGGVLPNI

NAVLLPKKSAKATEEKSPKSPAKKSPEKA*

>Dstrictus_W7

MDSSPAVAKPARGAGGRKGGDRKKSVSKSAKAGLQFPVGRIARFLKKGRYALRYGSGAPV

YLAAVLEYLAAEVLELAGNAARDNKKARINPRHLCLAIRNDEELGKLLHGVTIASGGVLP

NINPILLPKKSATSSQTEKSPPAAKSPKKA*

>Sparvula_W7

MESPAAAAKPARGAGGRRGGDRKKSVTKSAKAGLQFPVGRIARYLKKGRYAIRYGSGAPV

YLAAVLEYLAAEVLELAGNAARDNKKNRINPRHLCLAIRNDEELGKLLHGVTIASGGVLP

NINPILLPKRAPGQEKFSQATKSPKKA*

>Mperfoliatum_v2.1|Myper.0004s2359|Myper.0004s2359.1

METTGKVKKGFGGRKGGGPKSKSVSKSIKAGLQFPVGRITRFLKKGRYAQRLGGGAPVYM

AAVLEYLAAEVLELAGNAARDNKKSRIIPRHLLLAIRNDEELGKLLSGVTIAHGGVLPNI

NSVLLPKKSAKSTEEVASKSPVKSPKKA*

>Salba_v3.1|Sialb.0157s0397|Sialb.0157s0397.1

METTGKVKKAFGGRKAGGPKTKSVSKSIKAGLQFPVGRITRFLKKGRYAQRLGGGAPVYM

AAVLEYLAAEVLELAGNAARDNKKSRIIPRHLLLAIRNDEELGKLLSGVTIAHGGVLPNI

NSVLLPKKSAKSTEEVASKSPAKSPKKA*

>Salba_v3.1|Sialb.0336s0034|Sialb.0336s0034.1

MDAGAKVKKGAGGRRGGGPKKKPVSRSVKAGLQFPVGRIGRYLKKGRYSKRVGTGAPVYL

AAVLEYLAAEVLELAGNAARDNKKNRIIPRHVLLAVRNDEELGKLLRGVTIAHGGVLPNI

NPVLLPKKSEKAASTTKGTKSPSKATKSPKKA*

>Esalsugineum_W7

MESPAATTKPGRGGRRGGDRKKSVSKSVKAGLQFPVGRIARFLKKGRYAIRYGSGAPVYL

AAVLEYLAAEVLELAGNAARDNKKNRINPRHLCLAIRNDEELGKLLHGVTIASGGVLPNI

NPILLPKKAAASSQAEKPSPKSPKKA*

>Esalsugineum_v1.0|Thhalv10014933m.g|Thhalv10014933m

METTGKVKKGFGGRKAGGPKTKSVSKSIKAGLQFPVGRITRFLKKGRYAQRLGGGAPVYM

AAVLEYLAAEVLELAGNAARDNKKTRIIPRHLLLAIRNDEELGKLLSGVTIAHGGVLPNI

NAVLLPKKSSAKSAEDTASKSPAKSPKKA*

>Esalsugineum_v1.0|Thhalv10014932m.g|Thhalv10014932m

MESTGKVKKGFGGRKAGGPKTKSVSKSIKAGLQFPVGRITRFLKKGRYAQRLGGGAPVYM

AAVLEYLAAEVLELAGNAARDNKKTRIIPRHLLLAIRNDEELGKLLSGVTIAHGGVLPNI

NAVLLPKKSSAKSAEDTASKSPAKSPKKA*

>Esalsugineum_v1.0|Thhalv10014920m.g|Thhalv10014920m

MDSGTKVKKGASGRRSGGPKKKPVSRSVKAGLQFPVGRIGRYLKKGRYSKRVGTGAPVYL

AAVLEYLVAEVLELAGNAARDNKKNRIIPRHVLLAVRNDEELGKLLKGVTIAHGGVLPNI

NPILLPKKSEKAASPAKVPKSPSKAAKSPKKA*

>Cgrandiflora_W7

MESAAAATTASTKPARGAGGRKGGDRKKSVTKSVKAGLQFPVGRIARYLKKGRYAIRYGS

GAPVYLAAVLEYLAAEVLELAGNAARDNKKNRINPRHLCLAIRNDEELGKLLSGVTIASG

GVLPHINPVLLPKKSPSQAEKASPASKAPKKA*

>Cgrandiflora_v1.1|Cagra.1317s0026|Cagra.1317s0026.1

MESTGKVKKAFGGRKPGGPKTKSVSKSIKAGLQFPVGRITRFLKKGRYAQRLGGGAPVYM

AAVLEYLAAEVLELAGNAARDNKKTRIIPRHLLLAIRNDEELGRLLSGVTIAHGGVLPNI

HSVLLPKKTASTKSTEEKVSKSPVKSPKKA*

>Cgrandiflora_v1.1|Cagra.1317s0027|Cagra.1317s0027.1

MEATGKVKKAFGGRKAGGPKAKSVSKSIKAGLQFPVGRITRFLKKGRYAQRLGGGAPVYM

AAVLEYLAAEVLELAGNAARDNKKTRIIPRHLLLAIRNDEELGRLLSGVTIAHGGVLPNI

HSVLLPKKTASTKSTEEKVSKSPVKSPKKA*

>Bstricta_v1.2|Bostr.26833s0247|Bostr.26833s0247.1

METTGKVKKAFGGRKPGGPKTKSVSKSIKAGLQFPVGRITRFLKKGRYAQRLGGGAPVYM

AAVLEYLAAEVLELAGNAARDNKKTRIIPRHLLLAIRNDEELGKLLSGVTIAHGGVLPNI

HSVLLPKKTATKSTEEKATKSPVKSPKKA*

>Bstricta_W7

MDSGTKVKKGAAGRRGGGGPKKKPVSRSVKSGLQFPVGRIGRYLKKGRYSKRVGTGAPVY

LAAVLEYLAAEVLELAGNAARDNKKNRIIPRHVLLAVRNDEELGTLLKGVTIAHGGVLPN

INPILLPKKSEKAASTTKRPKSQSKATKSPKKA*

>Bstricta_v1.2|Bostr.29827s0086|Bostr.29827s0086.1

MESAAATKPARGAGGRKGGDRKKSVTKSVKAGLQFPVGRIARYLKKGRYAIRYGSGAPVY

LAAVLEYLAAEVLELAGNAARDNKKNRINPRHLCLAIRNDEELGKLLHGVTIASGGVLPN

INPILLPKKSTVEKASPATKSPKKA*

>Crubella_v1.1|Carub.0006s0136|Carub.0006s0136.1

MDSGTKVKKGAAGRRGGGGGPKKKPVSRSVKSGLQFPVGRIGRYLKKGRYSKRVGTGAPV

YLAAVLEYLAAEVLELAGNAARDNKKNRIIPRHVLLAVRNDEELGTLLKGVTIAHGGVLP

NINPILLPKKSERAASTTKTPKSPSKATKSPKKV*

>Crubella_W7

MESAAAATASTKPARGAGGRKGGDRKKSVTKSVKAGLQFPVGRIARYLKKGRYAIRYGSG

APVYLAAVLEYLAAEVLELAGNAARDNKKNRINPRHLCLAIRNDEELGKLLSGVTIASGG

VLPHINPVLLPKKSPSQAEKASPASKAPKKA*

>Crubella_v1.1|Carub.0008s2058|Carub.0008s2058.1

MEATGKVKKAFGGRKAGGPKAKSVSKSIKAGLQFPVGRITRFLKKGRYAQRLGGGAPVYM

AAVLEYLAAEVLELAGNAARDNKKTRIIPRHLLLAIRNDEELGRLLSGVTIAHGGVLPNI

NSVLLPKKTASTKSSEEKVSKSPVKSPKKA*

>Iamara_v1.1|Ibeam.0138s0019|Ibeam.0138s0019.1

MEKTGKVKKGFGGRKAGGKSVSKSIKAGLQFPVGRITRFLKKGRYAQRLGGGAPVYMAAV

LEYLAAEVLELAGNAARDNKKSRIIPRHLLLAIRNDEELGKLLSGVTIAHGGVLPNINSV

LLPKKSAAKPTEEKAPKSPAKSPKKA*

>Iamara_v1.1|Ibeam.0201s0009|Ibeam.0201s0009.1

METTGKVKKGFGGRKAGGPKAKSVSKSVKAGLQFPVGRITRFLKKGRYAQRLGGGAPVYM

AAVLEYLAAEVLELAGNAARDNKKTRIIPRHLLLAIRNDEELGKLLSGVTIAHGGVLPNI

NSVLLPKKTAAKSTEESAKKSPAKSPKKA*

>Iamara_v1.1|Ibeam.0034s0055|Ibeam.0034s0055.1

MDSGTKVKKGVAGRKGGGPKKKPVSRSVRSGLQFPVGRIGRYLKNGRYSKRVGTGAPVYL

AAVLEYLAAEVLELAGNAARDNKKNRIIPRHVLLAVRNDDELGRLLKGVTIAHGGVVPNI

NPILLPKKYERASSATKAPKSPSKATKSPKKT*

>Iamara_W7

MESPAVKSGRGTGGRRGGDRKKSVSKSVKAGLQFPVGRIARYLKKGRYALRYGSGSSVYL

AAVLEYLAAEVLELAGNAARDNKKNRINPRHLCLAIRNDEELGKLLHGVTIASGGVLPNI

NPILLPKKSAASSSQAEKVSKSPKKA*

>Mperfoliatum_v2.1|Myper.0004s0403|Myper.0004s0403.1

MHSGTRAKKGAGGRRGGGPKKKPVSRSVKAGLQFPVGRIGRYLKKGRYSKRVGTGAPVYL

AAVLEYLAAEVLELAGNAARDNKKSRIIPRHVLLAVRNDEELGKLLKGVTIAHGGVLPNI

NPILLPKRSEKAASTPKVPMSPSKPKTTKSPKKE*

>Mperfoliatum_v2.1|Myper.0004s2359|Myper.0004s2359.1

METTGKVKKGFGGRKGGGPKSKSVSKSIKAGLQFPVGRITRFLKKGRYAQRLGGGAPVYM

AAVLEYLAAEVLELAGNAARDNKKSRIIPRHLLLAIRNDEELGKLLSGVTIAHGGVLPNI

NSVLLPKKSAKSTEEVASKSPVKSPKKA*

>Mperfoliatum_W7

MESPAAAKPARGAGGRRGGDRKKSVSKSVKAGLQFPVGRISRYLKKGRYAIRYGSGAPVY

LAAVLEYLAAEVLELAGNAARDNKKNRINPRHLCLAIRNDEELGKLLHGVTIASGGVLPN

INPVLLPKRAAGSSQAEKASPAPKSPKKA*

>Lsativum_W7

MESQAATTKPGRGGRRGGDRKKSVTKSVKAGLQFPVGRIARYLKKGRYALRYGSGAPVYL

AAVLEYLAAEVLELAGNAARDNKKNRINPRHLCLAIRNDEELGKLLHGVTIASGGVLPNI

NPVLLPKKSATSSQAEKASPATKSPKKA*

>Lsativum_v1.1|Lesat.0134s0023|Lesat.0134s0023.1

METTTKVKKGFGGRKAGGPKAKSVSKSIKAGLQFPVGRITRFLKKGRYAQRLGGGAPVYM

AAVLEYLAAEVLELAGNAARDNKKTRIIPRHLLLAIRNDEELGKLLSGVTIAHGGVLPNI

NSVLLPKKSAKSTEEKATKSPAKSPKKA*

>Lsativum_v1.1|Lesat.0156s0126|Lesat.0156s0126.1

MNSEIKTKKGPFGRRSAGPKKKPVSRSVKSGLQFPVGRIGRYLKKGRYSKRVGTGAPVYL

AAVLEYLAAEVLELAGNAARDNKKNRIIPRHVLLAVRNDEELGKLLKGVTIAHGGVLPNI

NPVLLPKKSEKTTSPAKTPKSPSKPTKSPKKA*

>Lsativum_v1.1|Lesat.0059s0691|Lesat.0059s0691.1

MEPTTKVKKGFGGRKAGGPKAKSVSKSIKAGLQFPVGRITRFLKKGRYAQRLGGGAPVYM

AAVLEYLAAEVLELAGNAARDNKKTRIIPRHLLLAIRNDEELGKLLSGVTIAHGGVLPNI

NSVLLPKKSAKSTEEKPTKSPVKSPKKA*

>Tarvense_v1.1|Thlar.0089s0168|Thlar.0089s0168.1

MDSGSKVKKGAAGRKGGGPKKKPVSRSVKSGLQFPVGRIGRYLKKGRYSKRVGTGAPVYL

AAVLEYLAAEVLELAGNAARDNKKNRIIPRHVLLAVRNDEELGTLLKGVTIAHGGVLPNI

NPILLPNKSAKASSSSAKLPKSPSKATKSPKKAS*

>Tarvense_v1.1|Thlar.0018s0257|Thlar.0018s0257.1

MESTGKVKKGFGGRKGGGPKNKSVSKSIKAGLQFPVGRITRFLKKGRYAQRLGGGAPVYM

AAVLEYLAAEVLELAGNAARDNKKSRIIPRHLLLAIRNDEELGKLLSGVTIAHGGVLPNI

NSVLLPKKSAKSADEVASKSPAKSPKKA*

>Tarvense_v1.1|Thlar.0018s0251|Thlar.0018s0251.1

METTGKVKKGFGGRKAGGPKTKSVSKSIKAGLQFPVGRITRFLKKGRYAQRLGGGAPVYM

AAVLEYLAAEVLELAGNAARDNKKTRIIPRHLLLAIRNDEELGKLLSGVTIAHGGVLPNI

NSVLLPKKSAKSAEEKPAKSPVKSPKKA*

>Tarvense_W7

MESPAAAKPARGAGGRRGGDRKKSVTKSVKAGLQFPVGRIARFLKKGRYAIRYGSGAPVY

LAAVLEYLAAEVLELAGNAARDNKKTRINPRHLCLAIRNDEELGKLLHGVTIASGGVLPN

INPILLPKRVASSSQTEKASPASKSPKKA*

**Monocots**

>Spolyrhiza_v2|Spipo6G0070400|Spipo6G0070400

MEGAGKVKKGAGGRKGGGPKKKPVSRSVKAGLQFPVGRIGRYLKKGRYSKRVGTGAPVYL

AAVLEYLAAEVLELAGNAAKDNKKTRIIPRHVLLAVRNDEELGKLLSGVTIPHGGVLPNI

NPVLLPKKTERAGKEPKSPSKATKSPKKA*

>Spolyrhiza_v2|Spipo23G0023700|Spipo23G0023700

MEGGGKVKKGAGGRKGGGPKKKPVSRSVKAGLQFPVGRIGRYLRKGRYAQRVGTGAPVYL

AAVLEYLAAEVLELAGNAARDNKKNRIIPRHVLLAVRNDEELGKLLNGVTIAHGGVLPNI

NPVLLPKKTDKAAREAASPSKATKSPKKA*

>Macuminata_v1|GSMUA_Achr10G02580_001|GSMUA_Achr10T02580_001

MDAGGGGGKVKRGAAGRKGGGPRKKPVSRSVKAGLQFPVGRIGRYLKKGRYAQRVGTGAP

VYLAAVLELAGNAARDNKKNRIIPRHVLLAIRNDEELGKLLAGVTIAHGGVLPNINPVLL

PKKSNNAAKEPKSPSKATKSPKKA*

>Acomosus_v3|Aco001479|Aco001479.1

MEGGGGGGGKVKKGGRRGGGGGPRKKAVSRSVKAGLQFPVGRIGRYLKKGRYAQRVGTGA

PVYLAAVLEYLAAEVLELAGNAARDNKKNRIIPRHVLLAIRNDEELGKLLAGVTIAHGGV

LPNINPVLLPKKTDKAAKEPKSPSKPASTKSPKKP*

>Acomosus_v3|Aco017638|Aco017638.1

MADDDDDVAAKGKKRSGGGKAKKRSGGGKAKKGSAGAGGDGGGGGARKKSVSRSVKAGLQ

FPVGRIGRYLKNGRYAQRVGSGAPVYLAAVLEYLAAELLELAGNAAKDNKRNRITPRHVL

LAIRNDEEFAKLLSGVTIAHGGVLPNLNPALLPKKKNAAAKESNAPSEVPVEKSPSKV*

>Acomosus_v3|Aco010964|Aco010964.1

MEAGGGKVKKGAGGRRGGGGPKKKPVSRSIKAGLQFPVGRIGRFLKKGRYAQRVGTGAPV

YLAAVLEYLAAEVLELAGNAARDNKKNRIIPRHVLLAIRNDEELGKLLAGVTIAHGGVLP

NINPVLLPKKSSEKATKEPKSPSKSAATKSPKKA*

>Acomosus_v3|Aco014212|Aco014212.1

MDGGGKVKKGAAGRKGGGPKKKPVSRSVKAGLQFPVGRIGRYLKKGRYSQRVGTGAPVYL

AAVLEYLAAEVLELAGNAARDNKKNRIIPRHVLLAVRNDEELGKLLAGVTIAHGGVLPNI

NPVLLPKKSASKEPKSPTPKPTKSPKKA*

>Aofficinalis_V1.1|evm.TU.AsparagusV1_08.3497|evm.model.AsparagusV1_08.3497

MQEMVFRRRSSSAEGNFDLRQPAISGFECDGRCYRGLELRRVALFPGSSASSKRVLELAG

NAARDNKKNRIIPRHVLLAVRNDEELGKLLAGVTIAHGGVLPNINPVLLPKKSAAAKEDG

GKSPKKAAKSPKKAA*

>Dalata_v2.1|Dioal.18G105000|Dioal.18G105000.1

MDGGSKVKKGAGGRKGGGPKKKPVSRSVKAGLQFPVGRIGRFLKKGRYSQRVGSGAPVYL

AAVLEYLAAEVLELAGNAARDNKKNRIIPRHLLLAVRNDEELGKLLAGVTIAHGGVLPNI

NPVLLPKRSTAAKEPKSPSKATKSPKKA*

>Dalata_v2.1|Dioal.12G083900|Dioal.12G083900.1

METGGKIKKGAGGRKGGGPKKKPVSRSVKAGLQFPVGRIGRYLKKGRYAQRVGTGAPVYL

AAVLEYLAAEVLELAGNAARDNKKNRIIPRHVLLAIRNDEELGKLLAGVTIAHGGVLPNI

NPVLLPKKTDRPGKEAKSPSKAAKSPKKA*

>Dalata_v2.1|Dioal.09G074200|Dioal.09G074200.1;Dioal.09G074200.2;Dioal.09G074200.3

MESGAKLKKGAGGRKGGGPRKKPTSRSVKAGLQFPVGRIGRYLKKGRYSQRVGTGAPVYL

AAVLEYLAAEVLELAGNAARDNKKNRIIPRHVLLAVRNDEELGKLLAGVTIAHGGVLPNI

NPVLLPKKSAAAAKEPKSPSKVTKSPKKA*

>Jascendens_v1.1|Joasc.01G197600|Joasc.01G197600.1

MDGAGTGAGAGGKVKKAAAGRKLGGPKKKPVSRSVKAGLQFPVGRIGRFLKKGRYAQRVG

TGAPVYLAAVLEYLAAEVLELAGNAARDNKKNRIIPRHVLLAIRNDEELGKLLAGVTIAH

GGVLPNINPVLLPKKSEKAAAAKEPKSPKKATKSPKKA*

>Jascendens_v1.1|Joasc.12G107100|Joasc.12G107100.1

MEGATKVKKGAGGRRGGGGPKKKPVSRSVKAGLQFPVGRIGRYLKQGRYAQRVGTGAPVY

LAAVLEYLAAEVLELAGNAARDNKKNRIIPRHVLLAIRNDEELGKLLAGVTIAHGGVLPN

INPVLLPKKTAAAAAAKEAKSPKKATAKSPKKAAAAAS*

>Jascendens_v1.1|Joasc.03G009100|Joasc.03G009100.1

MEGAGGKVKKAAAGRKLGGPKKKPVSRSVKAGLQFPVGRIGRYLKKGRYAQRVGTGAPVY

LAAVLEYLAAEVLELAGNAARDNKKNRIIPRHVLLAIRNDEELGKLLAGVTIAHGGVLPN

INPVLLPKKTAEKSGTAKEGKKASKPPKSPKKAAA*

>Jascendens_v1.1|Joasc.02G046400|Joasc.02G046400.1

MDGVGTGAGVGRKVKKVAAERKLDRPKKKPVSHSVKAGLQFAIGCIGRFLKKGYYTQRVG

PGAPIYLATVHEYLAAEVLELARNAAGDDKKNQIIPRHVLLAIRNDEELGKLLAGITIVY

SSVLPNINPMLLPKKSEKVAAAAAAKEPKSPTRPPSPPKKA*

>Zmarina_v3.1|Zosma06g14780|Zosma06g14780.1

METGKVKKGAAGRRIGGPKKKSVSRSVRAGLQFPVGRVGRFLKKGRYAQRIGGAAPVYLA

AVLEYLAAEVLELAGNAAKDNKKGRIIPRHVLLAIRNDAELGKLLSGVTIANGGVLPNIN

PILLPKRTAKSGEEVSTKSPVKKIAKSPKKV*

>Zmarina_v3.1|Zosma03g15340|Zosma03g15340.1

MESSKIKGAGGRKGSVRTKPVSKSLKAGLQFPVGRVGRFLRRGRYAKRFGVGAPIYLAAV

LEYLAAEVLELAGNAARDNKKTRITPRHVLLAVRNDVELGKLLHGVTFSQGGVLPNIHSV

LLPKSGKAEDSVKSPKKA*

>Sbicolor_v3.1.1|Sobic.009G164900|Sobic.009G164900.1

MDAGAKVPKKAAAGRRGGGGPKKKPVSRSVKAGLQFPVGRIGRYLKQGRYAQRIGTGAPV

YLAAVLEYLAAEVLELAGNAARDNKKNRIIPRHVLLAIRNDEELGKLLAGVTIAHGGVLP

NINPVLLPKKTAAAAAKEGKEKKSPKKAATKSPKKAAAA*

>Sbicolor_v3.1.1|Sobic.009G015900|Sobic.009G015900.1

MDGGGAKAKKAAAGRKLGGGGPKKKPVSRSVKAGLQFPVGRIGRYLKKGRYAQRVGTGAP

VYLAAVLEYLAAEVLELAGNAARDNKKNRIIPRHVLLAIRNDEELGKLLAGVTIAHGGVL

PNINSVLLPKKAAEKAEKAASATKSPKKAAAAKSPKK*

>Sbicolor_v3.1.1|Sobic.001G009700|Sobic.001G009700.1

MDVSGAGAGGKAKKGAAGRKAGGPRKKSVSRSVKAGLQFPVGRIGRYLKKGRYAQRVGTG

APVYLAAVLEYLAAEVLELAGNAARDNKKTRIIPRHVLLAIRNDEELGKLLAGVTIAHGG

VLPNIHSVLLPKKVAEKAAKEPKSPKKAAKSPKKA*

>Sbicolor_v3.1.1|Sobic.001G416800|Sobic.001G416800.1

MDSTAAGAGGKAKKGAAGRKAGGPRKKSVSRSVKAGLQFPVGRIGRYLKKGRYAQRVGTG

APVYLAAVLEYLAAEVLELAGNAARDNKKTRIIPRHVLLAIRNDEELGKLLSGVTIAHGG

VLPNINPVLLPKKTAEKASTGGSKEAKSPKKAAKSPKKA*

>Sbicolor_v3.1.1|Sobic.001G416900|Sobic.001G416900.1

MDSTGAGAGGKVKKGAGGRKAGGGPRKKSVSRSVKAGLQFPVGRIGRYLKKGRYAQRVGT

GAPVYLAAVLEYLAAEVLELAGNAARDNKKTRIIPRHVLLAIRNDEELGKLLSGVTIAHG

GVLPNINPVLLPKKTAEKASSGGSKEPKSPKKAAKSPKKA*

>Sbicolor_v3.1.1|Sobic.002G276000|Sobic.002G276000.1

MDASGAGGKAKKGAAGRKAGGPRKKSVSRSVKAGLQFPVGRIGRYLKKGRYAQRVGTGAP

VYLAAVLEYLAAEVLELAGNAARDNKKTRIIPRHVLLAIRNDEELGKLLAGVTIAHGGVL

PNINPVLLPKKVAEKAASGGSKEAKSPKKAAKSPKKA*

>Zmays_RefGen_V4|Zm00001d021300|Zm00001d021300_T001

MDASGAGSKAKKGAAGRKAGGPRKKSVSRSVKAGLQFPVGRIGRYLKKGRYAQRVGTGAP

VYLAAVLEYLAAEVLELAGNAAKDNKKTRIVPRHVLLAIRNDVELGKLLAGVTIAHGGVL

PNINPVLLPKKVAEKASSGGSKESKSPKKAAKSPKKAAKSPKKA*

>Zmays_RefGen_V4|Zm00001d019045|Zm00001d019045_T001

MDASAAGAGGKAKKGAAGRKAGGPRKKSVTRSVKAGLQFPVGRIGRYLKKGRYAQRVGTG

APVYLAAVLEYLAAEVLELAGNAAKDNKKTRIIPRHVLLAIRNDEELGKLLSGVTIAHGG

VLPNINPVLLPKKTAEKAAAKEAKSPKKAAKSPKKA*

>Zmays_RefGen_V4|Zm00001d038381|Zm00001d038381_T001

MDAGAKVVKKAAAGRRGGGGPKKKPVSRSVKAGLQFPVGRIGRYLKQGRYSQRVGTGAPV

YLAAVLEYLAAELLELAGNAARDNKKNRIIPRHVLLAIRNDEELGKLLAGVTIAHGGVLP

NINPVLLPKKTAVAAAKEGKEKKSPKKAAAAKSPKKVAAS*

>Zmays_RefGen_V4|Zm00001d035619|Zm00001d035619_T001

MDGGGAKAKKAAAGRKLGGGGPKKKPVSRSVKAGLQFPVGRIGRYLKKGRYAQRVGTGAP

VFLAAVLEYLAAEVLELAGNAARDNKKTRIIPRHLLLAIRNDEELGKLLGGVTIAHGGVL

PNINPVLLPKKAAERAEKAAAAGPKSPKKVAAKSPKK*

>Zmays_RefGen_V4|Zm00001d044246|Zm00001d044246_T002

MDATGTGAGGKAKKGAAGRKAGGPRKKSVTRSVKAGLQFPVGRIGRYLKKGRYAQRVGSG

APVYLAAVLEYLAAEVLELAGNAAKDNKKTRIVPRHVLLAIRNDEELGKLLTGVTIAHGG

VLPNINPVLLPKKTAEKASSGGSKEAKSPKKAAKSPKKA*

>Zmays_RefGen_V4|Zm00001d047787|Zm00001d047787_T001

MDSTGTGAGGKGKKGAAGRKVGGPRKKSVSRSVKAGLQFPVGRIGRYLKKGRYAQRVGTG

APVYLAAVLEYLAAEVLELAGNAARDNKKTRIIPRHVLLAIRNDEELGKLLGGVTIAHGG

VLPNINPVLLPKKTAEKASSVGSKEAKSPKKAAKSPKKA*

>Zmays_RefGen_V4|Zm00001d012837|Zm00001d012837_T001

MDVSGAGGKAKKGAAGRKAGGPTKKSVSRSSRAGLQFPVSRVGRYLKKGRYAQRVGTGAP

VYLAAVLEYLAAEVLELAGNAARDNKKTRIIPRHVLLAIRNDEELGKLLAGVTIAHGGVL

PNIHTVLLPKKVAEKAAKEPKKAAKSPKKA*

>Sviridis_v2.1|Sevir.9G453900|Sevir.9G453900.1

MDATGTGAGGKVKKGAAGRKAGGPRKKSVSRSVKAGLQFPVGRIGRYLKKGRYAQRVGTG

APVYLAAVLEYLAAEVLELAGNAARDNKKTRIIPRHVLLAIRNDEELGRLLAGVTIAHGG

VLPNINPVLLPKKTAEKASSGGSKEAKSPKKAAKSPKKA*

>Sviridis_v2.1|Sevir.2G297800|Sevir.2G297800.1

MDVSGAGATGKVKKGAAGRKAGGPRKKSVSRSVKAGLQFPVGRIGRYLKKGRYAQRVGTG

APVYLAAVLEYLAAEVLELAGNAARDNKKTRIIPRHVLLAIRNDEELGKLLAGVTIAHGG

VLPNINPVLLPKKVAEKAASGGAKEAKSPKKGAKSPKKA*

>Sviridis_v2.1|Sevir.2G297700|Sevir.2G297700.1

MDASGATGKVKKGAAGRKAGGPRKKSVSRSVKAGLQFPVGRIGRYLKKGRYAQRVGTGAP

VYLAAVLEYLAAEVLELAGNAARDNKKTRIIPRHVLLAIRNDEELGKLLAGVTIAHGGVL

PNINPVLLPKKAAEKAASGGAKEAKSPKKAAKSPKKA*

>Sviridis_v2.1|Sevir.3G063800|Sevir.3G063800.1

MDGGAAKVKKAAVGRKLGGPKKKPVSRSVKAGLQFPVGRIGRYLKKGRYAQRVGSGAPVY

LAAVLEYLAAEVLELAGNAARDNKKNRIIPRHVLLAIRNDEELGRLLAGVTIAHGGVLPN

INPVLLPKKAAERAEKAEKAAKSPKKAATKSPKK*

>Sviridis_v2.1|Sevir.3G220700|Sevir.3G220700.1

MDVGAKVPKKGAAGRRGGGGPKKKPVSRSVKAGLQFPVGRIGRYLKQGRYSQRIGTGAPV

YLAAVLEYLAAEVLELAGNAARDNKKNRIIPRHVLLAIRNDEELGKLLAGVTIAHGGVLP

NINPVLLPKKTASAAAKEGKEGKEKKSPKKAATKSPKKAAAA*

>Ecoracana_v1.1|ELECO.r07.5BG0417980|ELECO.r07.5BG0417980.1

MDVGAKVPKKAGAAGRRGGGPKKKPVSRSVKAGLQFPVGRIGRYLKQGRYAKRIGTGAPV

YLAAVLEYLAAEVLELAGNAARDNKKNRIIPRHVLLAIRNDEELGKLLAGVTIAHGGVLP

NINPVLLPKKTASAAAKEGKETKEKKSPKKAATKSPKKA*

>Ecoracana_v1.1|ELECO.r07.3AG0243310|ELECO.r07.3AG0243310.1

MDSTGTGAGGKVKKGAAGRKTGGPRKKSVSRSVKAGLQFPVGRIGRYLKQGRYAQRVGTG

APVYLAAVLEYLAAEVLELAGNAARDNKKTRIIPRHVLLAIRNDEELGKLLCGVTIAHGG

VLPNINPVLLPKKTAEKASSGGSKETKSPKKAAAKSPKKVVDGK*

>Ecoracana_v1.1|ELECO.r07.6AG0528320|ELECO.r07.6AG0528320.1

MDVSGIGAVGKVKKAAAGRKTGGPRKKSVTRSVKAGLQFPVGRIGRYLKAGRYAQRVGSG

APVYLAAVLEYLAAEVLELAGNAARDNKKTRIIPRHVLLAIRNDEELGKLLSGVTIAHGG

VLPSIHSVLLPKKAAEKAASGGAKEPKSPKKATKSPKKA*

>Ecoracana_v1.1|ELECO.r07.5AG0407730|ELECO.r07.5AG0407730.1

MEAVGKVKKAAAGRKLGGGPKKKPVSRSVKAGLQFPVGRIGRYLKKGRYAQRVGTGAPVY

LAAVLEYLAAEVLELAGNAARDNKKNRIIPRHVLLAVRNDEELGRLLSGVTIAHGGVLPN

IHSVLLPKKAAERAEKAEKAGTKSPKKAATKSPKK*

>Phallii_v3.2|Pahal.3G093800|Pahal.3G093800.1

MDGGATKVKKAAAGRKLGGPKKKPVSRSVKAGLQFPVGRIGRYLKKGRYAQRVGTGAPVY

LAAVLEYLAAEVLELAGNAARDNKKNRIIPRHVLLAIRNDEELGKLLAGVTIAHGGVLPN

IHSVLLPKKAAEKAEKAAKSPKKAAAKSPKK*

>Phallii_v3.2|Pahal.3G094100|Pahal.3G094100.1

MDGGAAKEKKAAAGRKLGGPKKKPVSRSVKAGLQFPVGRIGRYLKKGRYAQRVGTGAPVY

LAAVLEYLAAEVLELAGNAARDNKKNRIIPRHVLLAIRNDEELGKLLAGVTIAHGGVLPN

IHSVLLPKKTAEKAEKAAKSPKKAAAKSPKK*

>Phallii_v3.2|Pahal.3G249800|Pahal.3G249800.1

MDVGTKVPRKAAAGRRGGGGPKKKPVSRSVKAGLQFPVGRIGRYLKQGRYSKRIGTGAPV

YLAAVLEYLAAEVLELAGNAARDNKRNRIIPRHVLLAIRNDEELGKLLAGVTIAHGGVLP

NINPVLLPKKTSSAATKESKEGKETKSPKKAATKSPKKAAAA*

>Phallii_v3.2|Pahal.2G344500|Pahal.2G344500.1

MDASGATGKVKKGAAGRKAGGPRKKSVSRSARAGLQFPVGRIGRYLKKGRYAQRVGTGAP

VYLAAVLEYLAAEVLELAGNAARDNKKTRIIPRHVLLAIRNDEELGKLLAGVTIAHGGVL

PNINPVLLPKKVAEKAASGAAKEAKSPKKGTKSPKKA*

>Phallii_v3.2|Pahal.2G344600|Pahal.2G344600.1

MDVSGAGGSGKVKKGAAGRKAGGPRKKSVSRSMKAGLQFPVGRIGRYLKKGRYAQRVGTG

APVYLAAVLEYLAAEVLELAGNAARDNKKTRIIPRHVLLAIRNDEELGKLLAGVTIAHGG

VLPNINPVLLPKKVAEKAAGAAAKEAKSPKKAAKSPKKA*

>Ufusca_v1.1|Urofu.9G472500|Urofu.9G472500.1;Urofu.9G472500.2

MDATGTGAGGKVKKGAAGRKAGGPRKKSVSRSVKAGLQFPVGRIGRYLKKGRYAQRVGTG

APVYLAAVLEYLAAEVLELAGNAARDNKKSRIIPRHVLLAIRNDEELGKLLSGVTIAHGG

VLPNINPVLLPKKTAEKASSGGSKEAKSPKKAAKSPKKA*

>Ufusca_v1.1|Urofu.2G294700|Urofu.2G294700.1

MDVSGAGATGKVKKGAAGRKAGGPRKKSVSRSVKAGLQFPVGRIGRYLKKGRYAQRVGTG

APVYLAAVLEYLAAEVLELAGNAARDNKKTRIIPRHVLLAIRNDEELGRLLAGVTIAHGG

VLPNINPVLLPKKVAEKAASGVSKEAKSPKKAAKSPKKA*

>Ufusca_v1.1|Urofu.2G294600|Urofu.2G294600.1

MDASGATGKVKKGAAGRKAGGPRKKSVSRSVKAGLQFPVGRIGRYLKKGRYAQRVGTGAP

VYLAAVLEYLAAEVLELAGNAARDNKKTRIIPRHVLLAIRNDEELGKLLAGVTIAHGGVL

PNINPVLLPKKAAEKAASGGAKEAKSPKKGAKSPKKA*

>Ufusca_v1.1|Urofu.3G250100|Urofu.3G250100.1

MDVGAKVPKKGAAGRRGGGGPKKKPVSRSVKAGLQFPVGRIGRYLKQGRYSQRIGTGAPV

YLAAVLEYLAAEVLELAGNAARDNKKNRIIPRHVLLAIRNDEELGKLLAGVTIAHGGVLP

NINPVLLPKKTASAAAKEGKEGKEKKSPKKAATKSPKKAAA*

>Ufusca_v1.1|Urofu.3G092900|Urofu.3G092900.1

MDGGGAKVKKAAAGRKFGGPKKKPVSRSVKAGLQFPVGRIGRYLKKGRYAQRV

GTGAPVYLAAVLEYLAAEVLELAGNAARDNKKNRIIPRHVLLAIRNDEELGKLLSGVTIA

HGGVLPNINPVLLPKKAAEKAEKAPKSPKKTATKSPKK*

>Osativa_v7.0|LOC_Os01g31800|LOC_Os01g31800.1

MDAAGAGAGGKLKKGAAGRKAGGPRKKAVSRSVKAGLQFPVGRIGRYLKKGRYAQRIGTG

APVYLAAVLEYLAAEVLELAGNAARDNKKNRIIPRHVLLAIRNDEELGKLLAGVTIAHGG

VLPNINPVLLPKKTAEKAAAAGKEAKSPKKAAGKSPKKA*

>Osativa_v7.0|LOC_Os05g02300|LOC_Os05g02300.1

MDVGVGGKAAKKAVGRKLGGPKKKPVSRSVKAGLQFPVGRIGRYLKKGRYAQRVGTGAPV

YLAAVLEYLAAEVLELAGNAARDNKKNRIIPRHVLLAIRNDEELGKLLAGVTIAHGGVLP

NINPVLLPKKTAEKADKPAKASKDKAAKSPKKQARS*

>Osativa_v7.0|LOC_Os05g38640|LOC_Os05g38640.1

MEVGAKVPKKAGAGGRRGGGGPKKKPVSRSVKAGLQFPVGRIGRYLKQGRYSQRIGTGAP

VYLAAVLEYLAAEVLELAGNAARDNKKNRIIPRHVLLAIRNDEELGKLLAGVTIAHGGVL

PNINPVLLPKKTGSAAAKEAKEGKTPKSPKKATTKSPKKAAAA*

>Bdistachyon_v3.1|Bradi2g23090|Bradi2g23090.1

MEVGSKAKKGGAAGRPRGGPKKKPVSRSVKAGLQFPVGRIGRYLKLGRYAKRVGTGAPVY

LAAVLEYLAAEVLELAGNAARDNKRNRIIPRHVLLAIRNDEELGKLLAGVTIAYGGVLPN

INPVLLPKKTAAAAAKEAKPGKEATAKSPRKATAKSPKKADAS*

>Bdistachyon_v3.1|Bradi2g37327|Bradi2g37327.1

MDGAGGKAKKGAVGKKLGGPRKKSVTRSVKAGLQFPVGRIGRFLKKGRYAQRVGSGAPVY

LAAVLEYLAAEVLELAGNAARDNKKTRIIPRHVLLAIRNDEELGRLLGGVTIAHGGVLPN

INPLLLPKKAAEKAGTEKVAAKSPKKPAGKSPKKAAAKA*

>Bdistachyon_v3.1|Bradi4g28560|Bradi4g28560.1

MDTGAGKAKKGAGGRKAGGGGGPRKKSVTRSVKAGLQFPVGRIGRYLKKGRYAQRVGSGA

PVYLAAVLEYLAAELLELAGNAARDNKKTRIIPRHLLLAIRNDDELGKLLAGVTIAHGGV

MPKINQVLLPKKTAAKEPKEPKSPKKAAKSPKKA*

>Bdistachyon_v3.1|Bradi1g66370|Bradi1g66370.1

MDVSGTGAGKAKKGAAGRKAGGPRKKSVTRSVRAGLQFPVGRIGRYLKKGRYAQRVGTGA

PVYMAAVLEYLAAEVLELAGNAARDNKKSRIIPRHVLLAVRNDDELGKLLAGVTIAHGGV

LPKINPILLPKKTAEKAPKEPKSPKKAATPKKADAKSPKKA*

>Bhybridum_v1.1|Brahy.D01G0910700|Brahy.D01G0910700.1

MEVSSTPAGKGKKGAAGRKVGGPRKKAVTRSVKAGLQFPVGRVGRLLKKGRYAQRVGSGA

PVYLAAVLEYLAAEVLELAGNAARDNKKSRIIPRHVLLAIRNDEELGKLLAGVTIAHGGV

LPNIHTVLLPKKTAEKAPKEAKEPKSPKKAATPKKAAKSPKKA*

>Bhybridum_v1.1|Brahy.D01G0910800|Brahy.D01G0910800.1

MDVSGTGAGKAKKGAAGRKAGGPRKKSVTRSVRAGLQFPVGRIGRYLKKGRYAQRVGTGA

PVYMAAVLEYLAAEVLELAGNAARDNKKSRIIPRHVLLAVRNDDELGKLLAGVTIAHGGV

LPKINPILLPKKTAEKAPKEPKSPKKAATPKKADAKSPKKA*

>Bhybridum_v1.1|Brahy.D04G0415600|Brahy.D04G0415600.1

MDTGAGKAKKGAGGRKAGGGGGPRKKSVTRSVKAGLQFPVGRIGRYLKKGRYAQRVGSGA

PVYLAAVLEYLAAELLELAGNAARDNKKTRIIPRHLLLAIRNDDELGKLLAGVTIAHGGV

MPKINQVLLPKKTAAKEPKEPKSPKKAAKSPKKA*

>Bhybridum_v1.1|Brahy.D02G0319000|Brahy.D02G0319000.1

MEVGSKAKKGGAAGRPRGGPKKKPVSRSVKAGLQFPVGRIGRYLKLGRYAKRVGTGAPVY

LAAVLEYLAAEVLELAGNAARDNKRNRIIPRHVLLAIRNDEELGKLLAGVTIAYGGVLPN

INPVLLPKKTAAAAAKEAKPGKEATAKSPRKATAKSPKKADAS*

>Bhybridum_v1.1|Brahy.D02G0494500|Brahy.D02G0494500.1

MDGAGGKAKKGAVGKKLGGPRKKSVTRSVKAGLQFPVGRIGRFLKKGRYAQRVGSGAPVY

LAAVLEYLAAEVLELAGNAARDNKKTRIIPRHVLLAIRNDEELGRLLGGVTIAHGGVLPN

INPLLLPKKAAEKAGTEKAAAKSPKKPAGKSPKKAAAKA*

>Taestivum_v2.2|Traes_6AS_F489CFAC9|Traes_6AS_F489CFAC9.2

MDASATVATGKGKKGAAGRKAGGPRKKSVSRSVKAGLQFPVSRIGRFLKKGRYAQRVGSG

APVYLAAVLEYLAAELLELAGNAAKDNKKSRIIPRHLLLAIRNDEELGKLLAGITIAHGG

VIPNINPVLLPKKTAEKSPKEPKSPKKTAKSPKKA*

>Taestivum_v2.2|Traes_4BL_96E367077|Traes_4BL_96E367077.9

MDVSGTGAGAKGKKGAAGRKAGGPRKKSVTRSVKAGLQFPVGRIGRYLKKGRYAQRVGTG

APVYLAAVLEYLAAELLELAGNAAKDNKKSRIIPRHLLLAVRNDEELGKLLAGVTIAHGG

VIPKINPVLLPKKTAEKEGKEPKSPKKATKSPKKATKA*

>Taestivum_v2.2|Traes_4BL_96E367077|Traes_4BL_96E367077.3

MEVSGAAAKGKKGAAGRKAGGPRKKSVTRSVKAGLQFPVGRIGRYLKKGRYAQRVGTGAP

VYLAAVLEYLAAELLELAGNAAKDNKKSRIIPRHLLLAVRNDEELGKLLAGVTIAHGGVI

PKINPVLLPKKTAEKEGKEPKSPKKATKSPKKATKA*

>Taestivum_v2.2|Traes_5BL_877AD3678|Traes_5BL_877AD3678.1

MDASAAGAISKAKKYVVGRKLGGGPRKKAVARSVKAGLQFPVGRIGRFLKKGRYAQRVGM

GAPVYLASVLEYLAAELLELAGNAAKDNKKSRIIPRHLLLAIRNDQELGKLLAGVTIAHG

GVLPNINPVLLPKKTAEKEPKSPKKATSSPKKA*

>Taestivum_v2.2|Traes_1AS_E774FAB9E|Traes_1AS_E774FAB9E.1

MDGSKAKKVAAKKFGGPRKKSVTKSIKAGLQFPVGRIGRYLKKGRYAQRVGSGAPVYLAA

VLEYLAAEVLELAGNAAKDNKKTRIVPRHLLLAIRNDQELGRLLSGVTIAHGGVIPNINP

VLLPKKAAEKAEKAGTAAKSPKKATKSPKKATKA*

>Taestivum_v2.2|Traes_1AS_6AA4114A2|Traes_1AS_6AA4114A2.1

MDASKLKKVAGKKFGGPRKKSVTRSVKAGLQFPVGRIGRYLKKGRYAQRVGSGAPVYLAA

VLEYLAAEVLELAGNAAKDNKKTRIVPRHLLLAIRNDQELGRLLSGVTIAHGGVIPNINP

VLLPKKAAEKAEKAGTAAKSPKKATKSPKKATKA*

>Taestivum_v2.2|Traes_6AS_2A59D8EDC|Traes_6AS_2A59D8EDC.1

MAGRKGGDRKKAVTRSVKAGLQFPVGRIGRYLKKGRYAQRVGSGAPVYLAAVLEYLAAEV

LELAGNAAKDNKKTRIIPRHLLLAVRNDQELRRLLAGVTIAHGGVIPNINSVLLPKKSPA

AAEKEAKSPKKKTAAKSPKKKTAATKE*

>Taestivum_v2.2|Traes_6AS_8B3190A99|Traes_6AS_8B3190A99.2

MDASATVATGKGKKGAAGRKAGGPRKKSVSRSVKAGLQFPVSRIGRFLKKGRYAQRVGSG

APVYLAAVLEYLAAELLELAGNAAKDNKKSRIIPRHLLLAVRNDQELGRLLAGVTIAHGG

VIPNINPVLLPKKTAEKSPKEPKSPKKTAKSPKKASKQSV

>Taestivum_v2.2|Traes_4AS_B97A404E2|Traes_4AS_B97A404E2.2

MEASGAAAKGKKGAAGRKAGGPRKKSVTRSVKAGLQFPVGRIGRYLKKGRYAQRVGTGAP

VYLAAVLEYLAAELLELAGNAAKDNKKSRIIPRHLLLAVRNDEELGKLLAGVTIAHGGVI

PKINPVLLPKRTAEKEGKEPKSPKKATKSPKKATKQCPPGARSPV

>Taestivum_v2.2|Traes_4AS_B97A404E2|Traes_4AS_B97A404E2.3

MEASGAAAKGKKGAAGRKAGGPRKKSVTRSVKAGLQFPVGRIGRYLKKGRYAQRVGTGAP

VYLAAVLEYLAAELLELAGNAAKDNKKSRIIPRHLLLAVRNDEELGKLLAGVTIAHGGVI

PKINPVLLPKRTAEKEGKEPKSPKKATKSPKKTTKA*

>Hvulgare_r1|HORVU6Hr1G029220|HORVU6Hr1G029220.3

MAGRKGGERKKAVTRSVKAGLQFPVGRIGRYLKKGRYAQRVGSGAPVYLAAVLEYLAAEV

YLLELAGNAAKDNKKTRIIPRHLLLAVRNDQELGRLLAGVTIAHGGVIPNINSVLLPKKS

PAAAEKEATKSPKKKAATKSPKKKTAATKE*

>Hvulgare_r1|HORVU6Hr1G006140|HORVU6Hr1G006140.1

MAGRKGGDRKKAVTRSVKAGLQFPVGRIGRYLKKGRYAQRVGSGAPVYLAAVLEYLAAEL

LELAGNAAKDNKKSRIIPRHLLLAVRNDQELGRLLAGVTIAHGGVIPNINSVLLPKKSPA

AAEKEAKSPKKKTATKSPKKKAPAAKE*

>Hvulgare_r1|HORVU6Hr1G005960|HORVU6Hr1G005960.10

MAGRKGGDRKKAVTRSVKAGLQFPVGRIGRYLKKGRYAQRVGSGAPVY

LAAVLEYLAAELLELAGNAAKDNKKTRIIPRHLLLAVRNDQELGRLLAGVTIAHGGVIPN

INSVLLPKKSPAAAEKEAKSPKKKAATKSPKKKEW*

>Hvulgare_r1|HORVU4Hr1G058940|HORVU4Hr1G058940.6

MEVSGAAAKGKKGAAGRKAGGPRKKSVTRSVKAGLQFPVGRIGRYLKKGRYAQRVGTGAP

VYLAAVLEYLAAELLELAGNAAKDNKKSRIIPRHLLLAVRNDEELGKLLAGVTIAHGGVI

PKINPVLLPKRTAEKEGKGPKSPKKATKSPKKATKSNVRNLSDPEA

>Hvulgare_r1|HORVU4Hr1G070250|HORVU4Hr1G070250.3;HORVU4Hr1G070250.5

MAGRKGGDRKKSVTRSVKAGLQFPVGRIGRYLKKGRYAQRVGSGAPVYLAAVLEYLAAEL

LELAGNAAKDNKKTRIIPRHLLLAVRNDQELGKLLAGVTIAHGGVIPNINSVLLPKKSPA

AAEKEAKSPKKKADTKSPKKKATKE*

>Tintermedium_v2.1|Thint.01G0011100|Thint.01G0011100.1

MDASKLKKVAGKKFGGPRKKSVTRSIKAGLQFPVGRIGRYLKKGRYAQRVGSGAPVYLAA

VLEYLAAEVLELAGNAAKDNKKTRIVPRHLLLAIRNDQELGRLLSGVTIAHGGVIPNINP

VLLPKKTAEKAEKAGAKSPKKATKSPKKATKA*

>Tintermedium_v2.1|Thint.15G0588500|Thint.15G0588500.1

MEASAAGAVPKVKRYVVGRKLGGGPRKQAVARSVKAGLQFPVGRIGRFLKKGRYAQRVGM

GAPVYLASVLEYLAAELLELAGNAAKDNKKSRIIPRHLLLAIRNDQELGRLLAGVTIAHG

GVLPNINPVLLPKKTAEKEPKSPKKAAMSPKKA*

>Tintermedium_v2.1|Thint.10G0265300|Thint.10G0265300.1

MDVSGAAAKGKKGADGRKAGGLRKKSVTRSVKAGLQFPVGRIGRYLKKGRYAQRVGTGAP

VYLAAVLEYLAAELLELAGNAAKDNKKSRIIPRHLLLAVRNDEELGKLLAGVTIAHGGVI

PKINPVLLPKKTAEKEGKEPKSPKKATKSPKKATKA*

>Tintermedium_v2.1|Thint.13G0076400|Thint.13G0076400.1

MDASATGAVSKVKKYVVGRKLGGGPRKKAVARSVKAGLQFPVGRIGRFLKKGRYAQRVGM

GAPVYLASVLEYLAAELLELAGNAAKDNKKSRIIPRHLLLTIRNDQELGKLLAGVTIAHG

GVLPNINPVLLPKKTAEKEPKSPKKADKSPKKA*

>Tintermedium_v2.1|Thint.17G0088400|Thint.17G0088400.1

MDASATVAAGKAKKGAAGRKAGGPRKKSVSRSVKAGLQFPVGRIGRFLKKGRYAQRVGSG

APVYLAAVLEYLAAELLELAGNAAKDNKKSRIIPRHLLLAVRNDQELGRLLAGVTIAHGG

VIPNINPVLLPKKTAEKSPKEPKSPRKTAKSPKKA*

>Tintermedium_v2.1|Thint.18G0052600|Thint.18G0052600.1

MAGRKGGDRKKAVTRSVKAGLQFPVGRIGRYLKKGRYAQRVGSGAPVYLAAVLEYLAAEV

LELAGNAAKDNKKTRIIPRHLLLAVRNDQELGRLLAGVTIAHGGVIPNINSVLLPKKSPA

AAEKEAKSPKKKAATKSPKKVAAKE*

>Tintermedium_v2.1|Thint.18G0090500|Thint.18G0090500.1

MAGRKGGERKKAVTRSVKAGLQFPVGRIGRYLKKGRYAQRVGSGAPVYLAAVLEYLAAEV

LELAGNAAKDNKKTRIIPRHLLLAVRNDQELGRLLAGVTIAHGGVIPNINSVLLPKKSPA

AADKESTKSPKKKTATKSPKKKAAATKE*

>Tintermedium_v2.1|Thint.18G0157100|Thint.18G0157100.1

MDASATVATGKGKKGAAGRKAGGPRKKSVSRSVKAGLQFPVSRIGRFLKKGRYAQRVGSG

APVYLAAVLEYLAAELLELAGNAAKDNKKSRIIPRHLLLAIRNDEELGKLLAGITIAHGG

VIPNINPVLLPKKTAEKSPKEPKSPKKTAKSPKKA*

>Tintermedium_v2.1|Thint.11G0169600|Thint.11G0169600.1

MDVSGTGAGAKGKKGAAGRKAGGPRKKSVTRSVKAGLQFPVGRIGRYLKKGRYAKRVGTG

APVYLAAVLEYLAAELLELAGNAAKDNKKSRIIPRHLLLAVRNDEELGKLLSGVTIAHGG

VIPKINPVLLPKRTAEKEGKEPKSPKKATKSPKKATKA*

>Tintermedium_v2.1|Thint.03G0357900|Thint.03G0357900.1

METGAKAPKKAGRRAGGGGGPKKKSVSRSVKAGLQFPVGRIGRYLKLGRYAKRVGSGAPV

YLAAVLEYLAAELLELAGNAARDNKKNRIIPRHVLLAIRNDEELGKLLAGVTIAYGGVLP

NINPVLLPKKAAAAATKEPKAGTTKSPRKSPAKKAADA*

**Dicots**

>Rcommunis_v0.1|29154.t000004|29154.m000210

METGGKVKKGAGGRKGGGPKKKPVTRSVRAGLQFPVGRIGRYLKKGRYAQRVGTGAPVYM

AAVLEYLAAEVLELAGNAARDNKKNRIIPRHVLLAVRNDEELGKLLAGVTIAHGGVLPNI

NPVLLPKKTEKATKEPTKSPSKATKSPKKA

>Rcommunis_v0.1|29154.t000006|29154.m000212

MESGGKVKKGAGGRKGGGPKKKPVTRSVRAGLQFPVGRIGRYLKKGRYAQRVGTGAPVYM

AAVLEYLAAEVLELAGNAARDNKKNRIIPRHVLLAVRNDEELGKLLAGVTIAHGGVLPNI

NPVLLPKKTEKATKEPTKSPSKATKSPKKA

>Rcommunis_v0.1|30128.t000274|30128.m008813

MDAPKEKKGAGGRRGGERKKSVSKSVKAGLQFPVGRIARFLKKGRYAQRFGSGAPVYLAA

VLEYLAAEVLELAGNAARDNKKNRINPRHVLLAVRNDEELGKLLHGVTIASGGVLPNINP

VLLPKKSAAGPADKPSEKSPKSPKKA

>Lusitatissimum_v1.0|Lus10023754.g|Lus10023754

MDTSGKLKKGAGGRKLGGPKKKPVSRSVKAGLQFPVGRIGRYLKKGRYSKRIGTGAPVYL

AAVLEYLAAEVLELAGNAARDNKKNRIIPRHVLLAVRNDEELGKLLAGVTIAHGGVLPNI

NPVLLPKKSASAEKATAAKATKSPTKAAKSPAKAAKSPAKAAKSPRKAVVH*

>Lusitatissimum_v1.0|Lus10040853.g|Lus10040853

MESAATKVKKGAGGRKGGGPKKKPVSRSVKAGLQFPVGRIGRYLKKGRYAQRVGTGAPVY

LAAVLEYLAAEVLELAGNAARDNKKNRIIPRHVLLAIRNDEELGKLLAGVTIAHGGVLPN

INPVLLPKKTDRATKEPKESTKSPAKAGKSPKKA*

>Lusitatissimum_v1.0|Lus10029899.g|Lus10029899

MDSVTETTTVGVTATPKGAGGRGRGARKKAVSKSTKAGLQFPVGRISRFLKRGRYSQRYG

AGAPIYLAAVLEYLAAEVLELAGNAARDNKKNRINPRHVLLAVRNDEELGKLLAGVTIAS

GGVLPNITPVLLPKKKISPEGSSSPVAEPKSPKKKKGTT*

>Lusitatissimum_v1.0|Lus10004502.g|Lus10004502

MDSATETTTAAVTATPKGAGGRGRGDRKKAVSKSTKAGLQFPVGRIFRFLKRGRYSQRYG

AGAPIYLAAVLEYLAAEVLELAGNAARDNKKNRINPRHVLLAVRNDEELGKLLAGVTIAS

GGVLPNINPVLLPKKKITPEGSSSPAAEPKSPKKKKTT*

>Lusitatissimum_v1.0|Lus10004945.g|Lus10004945

MDAGSTKVKKGAAVRKIGGPKKKPVSRSVKAGLQFPVGRIGRYLKKGRYAKRVGTGAPVY

LAAVLEYLAAEVLELAGNAARDNKKNRIIPRHVLLAIRNDEELGKLLAGVTIAHGGVLPN

INPVLLPKKSAAKEPAEKKSPAKATKSPKKA*

>Lusitatissimum_v1.0|Lus10005444.g|Lus10005444

MDAGTTKVKKGAAGRKIGGPKKKPVSRSLKAGLQFPVGRIGRYLKKGRYAQRVGTGAPVY

LAAVLEYLAAEVLELAGNAARDNKKNRIIPRHVLLAIRNDEELGKLLAGVTIAHGGVLPN

INPVLLPKKSEKAAAKEPAEKKASAKATKSPKKA*

>Spurpurea_Fish_Creek_v3.1|SpFC.05G022200|SpFC.05G022200.1

METTTKATKGAGGRRGGDRKKSVSKSTKAGLQFPVGRISRFLKKGRYAQRLGSGAPIYMA

AVLEYLAAEVLELAGNAARDNKKTRINPRHVLLAVRNDEELGKLLQGVTIASGGVLPNIN

PVLLPKKTSASEKSSGSEPKSPKKA*

>Spurpurea_Fish_Creek_v3.1|SpFC.13G018500|SpFC.13G018500.1

MEATKTTKGAGGRRGGERKKSVSKSTKAGLQFPVGRIARFLKKGRYAQRVGSGAPIYMAA

VLEYLAAEVLELAGNAARDNKKNRINPRHVLLAVRNDEELGKLLHGVTIANGGVLPNINP

VLLPKKSASSEKSSGSESKSPKKA*

>Spurpurea_v1.0|SapurV1A.0001s2110|SapurV1A.0001s2110.1

METGGKVKKGAAGRKGGGPKKKPVSRSAKAGLQFPVGRIGRYLKKGRYSQRVGSGAPVYL

AAVLEYLAAEVLELAGNAARDNKKNRIIPRHVLLAVRNDEELGKLLAGVTIAHGGVLPNI

NPVLLPKKTEKAAKEPKSPSKATKSPKKA*

>Ptrichocarpa_v3.1|Potri.006G082300|Potri.006G082300.1

METGGKVKKGAAGRKGGGPKKKPVSRSAKAGLQFPVGRIGRYLKKGRYSQRVGSGAPVYL

AAVLEYLAAEVLELAGNAARDNKKNRIIPRHVLLAVRNDEELGKLLAGVTIAHGGVLPNI

NPVLLPKKTEKAAKEPKSPSKATKSPKKA*

>PdeltoidesWV94_v2.1|Podel.05G027800|Podel.05G027800.1

MEATTKATKGAGGRRGGDRKKSVSKSIKAGLQFPVGRISRFLKKGRYAKRLGSGAPIYMA

AVLEYLAAEVLELAGNAARDNKKTRINPRHVLLAVRNDEELGKLLQGVTIASGGVLPNIN

PVLLPKKTSASEKSSGSEPKSPKKA*

>PdeltoidesWV94_v2.1|Podel.06G088700|Podel.06G088700.1

METGGKVKKGAAGRKGGGPKKKPVSRSAKAGLQFPVGRIGRYLKKGRYSQRVGSGAPVYL

AAVLEYLAAEVLELAGNAARDNKKNRIIPRHVLLAVRNDEELGKLLAGVTIAHGGVLPNI

NPVLLPKKTEKAAKEPKSPSKATKSPKKA*

>PdeltoidesWV94_v2.1|Podel.13G018600|Podel.13G018600.1

MEATTKATKGAGGRRGGERKKSVSKSTKAGLQFPVGRIARFLKKGRYAQRVGSGAPIYMA

AVLEYLAAEVLELAGNAARDNKKNRINPRHVLLAIRNDEELGKLLQGVTIASGGVLPNIN

SVLLPKKSASSEKSSGSEPKSPKKA*

>Ptrichocarpa_Stettler14_v1.1|PtStettler14.06G071600|PtStettler14.06G071600.1

METGGKVKKGAAGRKGGGPKKKPVSRSAKAGLQFPVGRIGRYLKKGRYSQRVGSGAPVYL

AAVLEYLAAEVLELAGNAARDNKKNRIIPRHVLLAVRNDEELGKLLAGVTIAHGGVLPNI

NPVLLPKKTEKAAKEPKSPSKATKSPKKA*

>Ptrichocarpa_v4.1|Potri.005G026500|Potri.005G026500.1

MEATTKATKGAGGRRGGDRKKSVSKSIKAGLQFPVGRISRFLKKGRYAKRLGSGAPIYMA

AVLEYLAAEVLELAGNAARDNKKTRINPRHVLLAVRNDEELGKLLQGVTIASGGVLPNIN

PVLLPKKTSGSEKSSGSEPKSPKKA*

>Ptrichocarpa_v4.1|Potri.013G018200|Potri.013G018200.1

MEATTKATKGAGGRRGGERKKSVSKSTKAGLQFPVGRIARFLKKGRYAQRVGSGAPIYMA

AVLEYLAAEVLELAGNAARDNKKNRINPRHVLLAIRNDEELGKLLQGVTIASGGVLPNIN

PVLLPKKSASSEKSSGSEPKSPKKA*

>Mesculenta_v7.1|Manes.10G076925|Manes.10G076925.1

MPSSRKVKKGAGGRKGGGPKKKPVSRSVKAGLQLSRWENRRYLKKGRYSQRVGTGAPVYL

AAVLEYLAAEVLELAGNAARDNKKNRIIPRHVLLAVRNDEELGKLLAGVTIAHGGVLPNI

NPVLLPKKSDKAAKEPKSPSKATKSPKKA*

>Mesculenta_v8.1|Manes.11G133400|Manes.11G133400.1

METGGKVKKGAGGRKGGGPKKKPTSRSVRAGLQFPVGRIGRYLKKGRYSQRVGTGAPVYL

AAVLEYLAAEVLELAGNAARDNKKNRIIPRHVLLAVRNDEELGKLLAGVTIAHGGVLPNI

NPVLLPKKTEKATKEPKSPSKATKSPKKA*

>Mesculenta_v8.1|Manes.04G031600|Manes.04G031600.1

MESGGKVKKGAGGRKGGGPKKKPVSRSVKAGLQFPVGRIGRYLKKGRYSQRVGTGAPVYL

AAVLEYLAAEVLELAGNAARDNKKNRIIPRHVLLAVRNDEELGKLLAGVTIAHGGVLPNI

NPVLLPKKSEKAAKEPKSPSKATKSPKKA*

>Cpapaya_ASGPBv0.4|evm.TU.supercontig_190.23|evm.model.supercontig_190.23

MDSATKVKKGAGGRKGGGPKKKPVSRSVKAGLQFPVGRIGRYLKKGRYSQRVGTGAPVYM

AAVLEYLAAEVLELAGNAARDNKKNRINPRHVLLAVRNDEELGKLLAGVTIAHGGVLPNI

NPVLLPKKSEKAAATKESKSPSKATKSPKKA*

>Cpapaya_ASGPBv0.4|evm.TU.supercontig_48.82|evm.model.supercontig_48.82

MDSDKPDKTTKRGRGRSAGERKKPVSKSTKAGLQFPVGRVGRYLRKGRYAQRYGTGAPIF

LAAVLEYLAAEVLELAGNAARDNKKTRINPRHLLLAVRNDEELGKLLQNVTIASGGVLPN

INPVLLPKRASSSETDKTPSTSKTKSPKKADKK*

>Aoccidentale_v0.9|Anaoc.0005s1194|Anaoc.0005s1194.1

MDAVTKVKKGAGGRRGGGPKKKPVSRSVKAGLQFPVGRIGRYLKKGRYSQRVGTGAPVYL

AAVLEYLAAEVLELAGNAARDNKKNRIIPRHVLLAVRNDEELGKLLAGVTIAHGGVLPNI

NPVLLPKKTEKAASKEPKSPSKATKSPKKA*

>Aoccidentale_v0.9|Anaoc.0020s0197|Anaoc.0020s0197.1

MESTGKIKKGAGGRKGGGPKKKPVSRSVKAGLQFPVGRIGRYLKKGRYSQRVGTGAPVYL

AAVLEYLAAEVLELAGNAARDNKKNRIIPRHVLLAVRNDEELGKLLAGVTIAHGGVLPNI

NPVLLPKKSEKLATKEAKSPSKATKSPRKA*

>Aoccidentale_v0.9|Anaoc.0001s0420|Anaoc.0001s0420.1

MESGGKLKKGAGGRKGGGPKKKPVSRSVKAGLQFPVGRIGRYLKYGRYAQRVGAGAPVYL

AAVLEYLAAEVLELAGNAARDNKKNRIIPRHVLLAVRNDEELGKLLAGVTIAYGGVLPNI

NPVLLPKKSEKATTKEAKSPSKATKSPKKA*

>Aoccidentale_v0.9|Anaoc.0010s0971|Anaoc.0010s0971.1

MDTGGKVKKGAGGRKGGGPKKKPISRSVKAGLQFPVGRIGRYLKKGRYSQRVGTGAPVYL

AAVLEYLAAEVLELAGNAARDNKKNRIIPRHVLLAVRNDEELGKLLAGVTIAHGGVLPNI

NPVLLPKKTEKAAGKESKSPKKA*

>Aoccidentale_v0.9|Anaoc.0715s0008|Anaoc.0715s0008.1

METSTKPVKGAGGRRGGDRKKAVSKSVKAGLQFPVGRIARFLKKGRYAQRVGTGAPVYMA

AVLEYLAAEVLELAGNAARDNKKNRINPRHVQLAVRNDDELGKLLQGVTIASGGVLPNIN

PVLLPKKTKSSDAENASKSPKKA*

>Tcacao_v2.1|Thecc.09G326500|Thecc.09G326500.1

MDTGGKVKKGAGGRKGGGPKKKPVSRSVKAGLQFPVGRIGRYLKKGRYSQRVGTGAPVYM

AAVLEYLAAEVLELAGNAARDNKKNRIIPRHVLLAVRNDEELGKLLAGVTIAHGGVLPNI

NPVLLPKKTDKATKEPKSPSKATKSLKKA*

>Tcacao_v2.1|Thecc.05G044600|Thecc.05G044600.1

MDAGAKVKKGAGGRKGGGPKKKPVSRSVKAGLQFPVGRIGRYLKKGRYSQRVGTGAPVYL

AAVLEYLAAEVLELAGNAARDNKKNRIIPRHVLLAVRNDEELGKLLAGVTIAHGGVLPNI

NPVLLPKKNDKAPKEPKSPSKATKSPKKSPKKA*

>Tcacao_v2.1|Thecc.05G311700|Thecc.05G311700.1

MESTAKPAGGRRGERKKAVSKSVKAGLQFPVGRIARFLKKGRYAQRYGAGAPIYLAAVLE

YLAAEVLELAGNAARDNKKNRINPRHLLLAVRNDEELGKLLQGVTIASGGVLPNINPVLL

PKKTSASSESDKVSKPKSPKKA*

>Klaxiflora_v1.1|Kalax.0004s0140|Kalax.0004s0140.1

MEKKGAGGRKGGGPKKKAVSRSLKAGLQFPVGRIGRFLKKGRYAQRVGSGAPVYLAAVLE

YLAAEVLELAGNAARDNKKTRIIPRHLLLAIRNDEELGKLLSGVTIAHGGVLPNINPVLL

PKKTGTGAEKEPKSPAKKSPKKA*

>Klaxiflora_v1.1|Kalax.0831s0006|Kalax.0831s0006.1

MEKKGAGGRKAGGPKKKGVSRSLKAGLQFPVGRIGRYLKNGRYSQRVGSGAPVYLAAVLE

YLAAEVLELAGNAARDNKKTRIIPRHLLLAIRNDEELGKLLSGVTIAHGGVLPNINPVLL

PKKTGSAAEKELKSPAAKKSPKKG*

>Klaxiflora_v1.1|Kalax.0831s0007|Kalax.0831s0007.1

MEKKGAGGRKAGGTKKKGVSRSLKAGLQFPVGRIGRFLKKGRYAQRVGSGAPVYLAAVLE

YLAAEVLELAGNAARDNKKTRIIPRHVLLAIRNDEELGKLLSGVTIAQGGVLPNINPVLL

PKKTGDAAGKEPKSPKKAKA*

>Kfedtschenkoi_v1.1|Kaladp0058s0633|Kaladp0058s0633.1

MEKKGAGGRKGGGPKKKSVSRSLKAGLQFPVGRIGRFLKKGRYAQRVGSGAPVYLAAVLE

YLAAEVLELAGNAARDNKKTRIIPRHVLLAIRNDDELGKLLSGVTIAHGGVLPNINPVLL

PKKTGTAAEKEPKSPAAKSPKKA*

>Kfedtschenkoi_v1.1|Kaladp0005s0027|Kaladp0005s0027.1;Kaladp0005s0027.2

MEKKGAGGRKAGGPKKKGVSRSLKAGLQFPVGRIGRYLKNGRYAQRVGSGAPVYLAAVLE

YLAAEVLELAGNAARDNKKTRIIPRHLLLAIRNDEELGKLLSGVTIAHGGVLPNINPVLL

PKKTGSAAEKEPRSPAAKKSPKKG*

>Kfedtschenkoi_v1.1|Kaladp0005s0028|Kaladp0005s0028.1

MEKKGAGGRKAGGPKKKGVSRSLKAGLQFPVGRIGRFLKKGRYAQRVGSGAPVYLAAVLE

YLAAEVLELAGNAARDNKKTRIIPRHVLLAIRNDEELGKLLSGVTIAQGGVLPNINPVLL

PKKTGDAAGKEPKSPKKAKA*

>Cquinoa_v1.0|AUR62001828|AUR62001828-RA

MDSTTGGKAKKGAAGRKGGGPKKKSVTRSVKAGLQFPVGRIGRYLKKGRYAQRVGSGAPV

YLAAVLEYLAAEVLELAGNAARDNKKNRIIPRHVLLAVRNDDELGKLLSGVTIAHGGVLP

NINPVLLPKKTGDKAAKDVKPSPKGVKSPKKA*

>Cquinoa_v1.0|AUR62015657|AUR62015657-RA

MDTGGKAKKGAGGRKGGGPKKKPVTRSVRAGLQFPVGRIGRYLKKGRYAQRVGTGAPVYL

AAVLEYLAAEVLELAGNAARDNKKNRIIPRHVLLAVRNDDELGKLLAGVTIAHGGVLPNI

NPVLLPKKTAEKSPKEPKSPKATKSPKKA*

>Ahypochondriacus_v2.1|AH022509|AH022509-RA

MDTAGGKGKKGAAGRKGGGPKKKPVSRSVKAGLQFPVGRIGRYLKKGRYAQRVGTGAPVY

LAAVLEYLAAEVLELAGNAARDNKKNRIIPRHVLLAVRNDDELGKLLAGVTIAHGGVLPN

INPVLLPKKTADKAPKEPKSPSKATKSPKKA*

>Ahypochondriacus_v2.1|AH010493|AH010493-RA

MESTKTSTKGAGGRGGGERKKAVRKSVKAGLQFPVGRIARLLKKGRYAQRVGSGAAVYLA

AVLEYLAAEVLELAGNAARDNKKNRIIPRHVLLAIRNDDELGKLLHGVTIAHGGVLPSIN

PVLLPKKKESASGDTPAKQTAKKSPKKA*

>Ahypochondriacus_v2.1|AH008153|AH008153-RA

MDSTGGKSKKGAAGRKGGGPKKKPVSRSVKAGLQFPVGRIGRYLKKGRYAQRVGTGAPVY

LAAVLEYLAAEVLELAGNAARDNKKNRIIPRHVLLAVRNDDELGKLLAGVTIAHGGVLPN

INPVLLPKKTTEKSPKEPKSPSKGTKSPKKA*

>Bvulgaris_EL10_1.0|EL10Ac4g09933|EL10Ac4g09933.1

MESGKTTSKGAAGRRGGDRKKSVTRSVKAGLQFPVGRIGRFLKKGRYAQRVGSGAPVYLA

AVLEYLAAEVLELAGNAARDNKKSRIIPRHVLLAIRNDDELGKLLHGVTIAHGGVLPNIN

PILLPKRTESSKKENTPVSPSKATKSPKKA*

>Bvulgaris_EL10_1.0|EL10Ac4g07945|EL10Ac4g07945.1

MDTAGGKGKKGAAGRKGGGPKKKPVSRSVKAGLQFPVGRIGRYLKKGRYAQRVGTGAPVY

LAAVLEYLAAEVLELAGNAARDNKKNRIIPRHVLLAVRNDDELGKLLAGVTIAHGGVLPN

INPVLLPKKTAEKAAKEPKSPKAAKSPKKA*

>Bvulgaris_EL10_1.0|EL10Ac7g15973|EL10Ac7g15973.1

MDSTAGGKAKKGAGGRKGGGPKKKPVSRSVKAGLQFPVGRIGRYLKKGRYAQRVGTGAPV

YLAAVLEYLAAEVLELAGNAARDNKKNRIIPRHVLLAVRNDDELGKLLSGVTIAHGGVLP

NINPVLLPKKAGGDKATKEPKSPSKATKSPKKA*

>Soleracea_Spov3|Spov3_chr2.02336|Spov3_chr2.02336

MDSAAGGKGKKGAAGRKGGGPKKKSVTRSVKAGLQFPVGRIGRFLKKGRYAQRVGSGAPV

YLAAVLEYLAAEVLELAGNAARDNKKSRIIPRHVLLAVRNDDELGKLLAGVTIAHGGVLP

NINPILLPKKTAEKAPKEAKPSTKAAKSPKKA*

>Soleracea_W7

METTKKTTKGAGGRGGRGSGERKKSVTKSLKAGLQFPVGRISRFLKKGRYAQRLSSASPV

YLAAVLEYLAAEVLELAGNAARDNKKSRIIPRHLLLAIRNDDELGKLLHGVTIANGGVLP

NIHSVLLPKKKESQTPPSPSGKDKSKADKSPKKTPKKA*

>Soleracea_Spov3|Spov3_chr4.01125|Spov3_chr4.01125

MDSAGKGKKGAGGRKGGGPKKKPVTRSVRAGLQFPVGRIGRFLKKGRYAQRVGTGAPVYL

AAVLEYLAAEVLELAGNAARDNKKTRIIPRHVLLAVRNDDELGKLLAGVTIAHGGVLPNI

NPVLLPKKVAEKSPKEPKSPSKATKSPKKA*

>Dcarota_v2.0|DCAR_006000|DCAR_006000

MESPAAGKVKKGAAGRKAGGPKKKSVTRSVRAGLQFPVGRIGRYLKKGRYAQRVGTGAPV

YLAAVLEYLAAEVLELAGNAARDNKKSRIIPRHLLLAIRNDEELGKLLGGVTIAHGGVLP

NINPVLLPKKTTEKAAKEPTAKVAGKSPKKAAK*

>Dcarota_v2.0|DCAR_018893|DCAR_018893

METGGKAKKGFGGRKGGGPRKKAVTRSVRAGLQFPVGRIGRYLKKGRYAQRVGTGAPVYL

AAVLEYLAAEVLELAGNAARDNKKTRIIPRHVLLAVRNDEELGKLLAGVTFAHGGVLPNI

NPVLLPKKTAEKAPKEPKSPAKAGKSPKKA*

>Dcarota_v2.0|DCAR_014835|DCAR_014835

MEGGGKLKKGAAGRKGDGPRKKSVTRSVKAGLQFPVGRIGRYLKKGRYAKRVGTGAPVYL

AAVLEYLAAEVLELAGNAARDNKKSRIIPRHLLLAIRNDEELGKLLAGVTIAHGGVLPNI

NPVLLPKKSEKAAAAKEPTKSPSKAAKSPKKA*

>Dcarota_W7

MEGGGKVKKGAAGRKAGGPKKKPVSRSVKAGLTFPVGRIGRFLKKGRYAQRVGTGAPVYL

AAVLEYLAAEVLELAGNAARDNKKSRIIPRHLLLAIRNDEELGKLLAGVTIAHGGVLPNI

NPVLLPKKSEKASQESTKSPSKATKSPRKAAA*

>Slycopersicum_ITAG2.4|Solyc11g073250.1|Solyc11g073250.1.1

MESTGKVKKGAAGRRGGGPKKKPVSRSVKAGLQFPVGRIGRFLKKGRYAQRVGSGAPVYL

AAVLEYLAAEVLELAGNAARDNKKNRIIPRHLLLAVRNDEELGKLLAGVTIAHGGVLPNI

NPILLPKKTGGEKAPKSPSKATKSPKKA*

>Slycopersicum_ITAG2.4|Solyc11g073260.1|Solyc11g073260.1.1

MESTGKMKKGAAGRRGGGPKKKPVSRSVKAGLQFPVGRIGRFLKKGRYAQRVGSGAPVYL

AAVLEYLAAEVLELAGNAARDNKKNRIIPRHLLLAVRNDEELGKLLAGVTIAHGGVLPNI

NPILLPKKTGGEKAPKSPKKA*

>Stuberosum_v4.03|PGSC0003DMG400024748|PGSC0003DMT400063677

MDATKTTKGAGGRKGGPRKKSVTKSIKAGLQFPVGRIGRYLKKGRYAQRVGSGAPIYLAA

VLEYLAAEVLELAGNAARDNKKSRIIPRHVLLAVRNDEELGKLLAGVTIASGGVLPNINP

ILLPKKSAVADEKAPKAKAAKSPKKA*

>Stuberosum_v4.03|PGSC0003DMG400008930|PGSC0003DMT400023064

MEKKGAGGRKGGGPKKKSVTRSVKAGLQFPVGRIGRYLKKGRYSQRVGSGAPVYLAAVLE

YLAAEVLELAGNAARDNKKNRIIPRHVLLAVRNDEELGKLLAGVTIAHGGVLPNINPILL

PKKSDKVGKEPGKSPSKATKSPRKA*

>Stuberosum_v4.03|PGSC0003DMG400015565|PGSC0003DMT400040216;PGSC0003DMT400040217;PGSC0003DMT400040218

MESTGKVKKGAAGRRGGGPKKKPVSRSVKAGLQFPVGRIGRFLKKGRYAQRVGSGAPVYL

AAVLEYLAAEVLELAGNAARDNKKNRIIPRHLLLAVRNDEELGKLLAGVTIAHGGVLPNI

NPILLPKKTGGEKAPKSPSKATKSPKKA*

>Oeuropaea_v1.0|Oeu036178.1|Oeu036178.1

MDTGGKLKKGAGGRRGGGPKKKPVSRSVKAGLQFPVGRIGRYLKKGRYSQRVGTGAPVYM

AAVLEYLAAEVLELAGNAARDNKKNRIIPRHVLLAVRNDEELGKLLAGVTIAYGGVLPNI

NPVLLPKKSEKAGKEPTKSPTKATKSPKKA*

>Oeuropaea_W7

TKGVGGRKGGDRKKAIPKSVKAGLQFPVGRIARFLKK

GRYAQRVGIGAPIYMAAVLEYLAAEVLELAGNAARDNKKSRIIPRHLQLAIRNDDELGKL

MHGVTIASGGVLPNINPVLLPKKSTANESQDKATKSPKASKSPKKA*

>Oeuropaea_v1.0|Oeu041024.1|Oeu041024.1

MESGKIKKGAGGRKGGGPKKKPVTRSVRAGLQFPVGRIGRYLKKGRYAQRVGTGAPVYLA

AVLEYLAAEVLELAGNAARDNKKNRIIPRHVLLAVRNDEELGKLLQGVTIAHGGVLPNIN

PVLLPKKTGGDKATKEPKSPSKATKSPKKAAA*

>Carabica_v0.5|evm.TU.Scaffold_636.583|evm.model.Scaffold_636.583

MDATKPTKGAGGRKGGERKKAVTKSVKAGLQFPVGRIARFLKKGRYAQRTGIGAPIYMAA

VLEYLAAEVLELAGNAARDNKKNRINPRHVLLAVRNDEELGKLLQGVTIASGGVLPNINP

VLLPKKTAASEEKAATKQSKSPKKA*

>Carabica_v0.5|evm.TU.Scaffold_601.326|evm.model.Scaffold_601.326

MEAAGKVKKGAGGRKGGGPKKKPVSRSTKAGLQFPVGRIGRYLKKGRYSERVGTGAPVYM

AAVLEYLAAEVLELAGNAARDNKKNRIIPRHVLLAIRNDEELGKLLAGVTIAHGGVLPNI

NPVLLPKKTDKATKEPTKSPKATKSPSKAAKSPKKAAAA*

>Carabica_v0.5|evm.TU.Scaffold_571.332|evm.model.Scaffold_571.332

MESAGGKVKKGAGGRKGGGPKKKPVSRSVKAGLQFPVGRIGRYLKKGRYSERVGTGAPVY

MAAVLEYLAAEVLELAGNAARDNKKNRIIPRHVLLAVRNDEELGKLLAGVTIAHGGVLPN

INPVLLPKKNEKAAKEPVKSPAKATKSPKKA*

>Lsativa_V8|Lsat_1_v5_gn_9_87120|Lsat_1_v5_gn_9_87120.1

MESGKTTKGAGGRKGAGERKKSVTKSVKAGLQFPVGRISRFLKRGRYAKRTGSGAPIYLA

AVLEYLAAEVLELAGNAARDNKKTRINPRHVLLAVRNDEELGKLLAGVTIASGGVLPNIN

PVLLPKKSAAVDAEKTPKSPKKAAKSPKKA*

>Lsativa_V8|Lsat_1_v5_gn_9_22420|Lsat_1_v5_gn_9_22420.1

MEGTGKVKKGAAGRKGGPRKKSVTRSVKAGLQFPVGRIGRFLKNGRYAKRVGTGAPVYLA

AVLEYLAAEVLELAGNAARDNKKHRIIPRHLLLAVRNDEELGKLLAGVTIAHGGVLPNIN

PILLPKKTAAAAEPKSPSKAAKSPKKAAKA*

>Lsativa_V8|Lsat_1_v5_gn_6_32381|Lsat_1_v5_gn_6_32381.1

METSGKVKKGAAGRKAGGPKKKSVSKSVKAGLQFPVGRIGRHIKNGRYAKRVGSGAPIYL

AAVLEYLAAEVLELAGNAARDNKKKRIIPRHVLLAVRNDEELGKLLAGVTIAHGGVLPNI

NPVLLPKKSLETATKEPKSPAKAAKSPKKA*

>Lsativa_V8|Lsat_1_v5_gn_8_77801|Lsat_1_v5_gn_8_77801.1

METTGKAKKGAGGRKGGGPRKKSVTRSVKAGLQFPVGRIGRYLKVGRYAKRVGTGAPVYL

AAVLEYLAAEVLELAGNAARDNKKTRIIPRHVLLAIRNDDELGKLLGGVTIAHGGVLPNI

HAVLLPKKSATTEPKSPSKAAKSPKKAAKTPKKAE*

>Lsativa_V8|Lsat_1_v5_gn_8_128160|Lsat_1_v5_gn_8_128160.1

MESGKTTKGAGGRKGAGERKKLVTKSVKAGLQFPVGRISRFLKKGRYAQRTGSGAPIYLA

AILEYLTAEVLELAGNAARDNKKKRINPRHVLLAVRNDEELGKLLAGVTIANGGVLPNIN

PVLLPKKSAVDTEKTPKSPKSTKAFKSHKKA*

>Lsativa_V8|Lsat_1_v5_gn_2_110521|Lsat_1_v5_gn_2_110521.1

MDAGTKVKKGAAGRKAGGPKKKPVSRSVKAGLQFPVGRLGRFLKKGQYAKRVGSGAPVYL

AAVLEYLAAEVLELAGNAARDNKKTRIIPRHVLLAVRNDEELGKLLSGVTIAHGGVLPNI

NPVLLPKKALDKATKEPKSPSKATKSPKKA*

>Lsativa_V8|Lsat_1_v5_gn_5_168621|Lsat_1_v5_gn_5_168621.1

MEGAAKSKKGAGGRKAGGPRKKAVTRSVKAGLQFPVGRIGRFLKKGRYAQRVGSGAPVYL

AAVLEYLAAEVLELAGNAARDNKKTRIIPRHLLLAIRNDEELGKLLGGVTIAHGGVLPNI

NPILLPKKTAAKEPSTPSKAAKSPKKAKKAE*

>Lsativa_V8|Lsat_1_v5_gn_5_168940|Lsat_1_v5_gn_5_168940.1

MDGDGKVKKGAGGRKADGSRKKAVSRSVKAGLQFPVGRIGRFLKLGRYARRVGSGAPIYL

AAVLEYLAAELLELAGNAARDNKKTRIIPRHLLLAIRNDDELGKLLGGVTIAHGGVLPNI

NPVLLPKKTAAKEPKSPSKATKSPKKDKKAE*

>Lsativa_V8|Lsat_1_v5_gn_5_168661|Lsat_1_v5_gn_5_168661.1

MKQTPKDIKKLLILHLRSIIGYYRKKAVSRSVKAGLQFPVGRIGRFLKLGRYARRVGSGA

PIYLAAVLEYLAAELLELAGNAARDNKKTRIIPRHLLLAIRNDDELGKLLGGVTIAHGGV

LPNINPVLLPKKTAAKEPKSPSKATKSPKKDKKAE*

>Hannuus_r1.2|HanXRQChr16g0511901|HanXRQChr16g0511901

MEAGKTGKAAGGRKGAANRKKSVTKSVKAGLQFPVGRIARFLKKGRYAQRTGSGAPIYLA

AVLEYLAAEVLELAGNAARDNKKTRINPRHVQLAVRNDEELGKLLAGVTIASGGVLPNIN

PVLLPKKSSSAEVEKTPKSTKSPKKLRT*

>Hannuus_r1.2|HanXRQChr07g0196031|HanXRQChr07g0196031

MESKKGAGGRKAGGPKKKPVSRSVKAGLQFPVGRLGRYLKKGRYARRVGTGAPVYLAAVL

EYLAAEVLELAGNAARDNKKNRIIPRHVLLAVRNDEELGKLLAGVTIAHGGVLPNINPVL

LPKKAAAKEATKSPAKVTKSPKKA*

>Hannuus_r1.2|HanXRQChr05g0134281|HanXRQChr05g0134281

MEATGKAKKGAAGRKAGGPRKKAVTRSIRAGLQFPVGRIGRYLKNGRYAKRVGTGAPVYL

AAVLEYLAAEVLELAGNAARDNKKNRINPRHVLLAIRNDDELGKLLAGVTIAHGGVLPNI

NPVLLPKKTAAKEPKSPAKATKSPKKAAA*

>Hannuus_r1.2|HanXRQChr13g0396031|HanXRQChr13g0396031

MEATRKAKKGAAGHKAGGPRRKAITRSIRAGLQFPVGRIGGYLKNGRYAKRVGTGAPVYL

APVLEYLAVGVLELAGNAARDNKKNRINPRHILLAIRNDDELGKFLVGVTIAHGGVLPNI

NLVLLPKKTAAKESKSP*

>Hannuus_r1.2|HanXRQChr14g0459291|HanXRQChr14g0459291

MEAAGKSKKGAAGRKAGGPKKKPVTRSIKAGLQFPVGRIGRYLKVGRYAKRVGTGAPVYL

AAVLEYLAAEVLELAGNAARDNKKNRIIPRHVLLAVRNDDELGKLLSGVTIAHGGVLPNI

NPVLLPKKAHDKATKEPKSPSKSTKSPKKAT*

>Hannuus_r1.2|HanXRQChr14g0453991|HanXRQChr14g0453991

MEAGGKLKKGAAGRKAGGPKKKPVTRSVKAGLQFPVGRIGRYLKVGRYAKRVGTGAPVYL

AAVLEYLAAEVLELAGNAARDNKKNRINPRHVLLAVRNDDELGKLLAGVTIAHGGVLPNI

NPVLLPKKSHDKAAKEPKSPAKPTKSPKKA*

>Hannuus_r1.2|HanXRQChr06g0175791|HanXRQChr06g0175791

METTGKAKKGAAGRKAGGPRKKSVTRSVKAGLQFPVGRIGRYLKHGRYAKRVGTGAPVYL

AAVLEYLAAEVLELAGNAARDNKKNRITPRHVLLAIRNDDELGKLLAGVTIAHGGVLPNI

NPVLLPKKSAAAAKETKSPVKPGKSPKKAA*

>Hannuus_r1.2|HanXRQChr11g0339501|HanXRQChr11g0339501

MESGKATKAAGGRKGAGERKKSVTKSVKAGLQFPVGRIARFLKKGRYAQRTGSGAPIYLA

AVLEYLAAEVLELAGNAARDNKKTRINPRHLLLAVRNDEELGKLLAGVTIASGGVLPNIN

PVLLPKKSSSAAEAEKTPKSKKSPKKA*

>Hannuus_r1.2|HanXRQChr08g0227841|HanXRQChr08g0227841

MESTGKVKKGAAGRKAGGPRKKAVTRSVKAGLQFPVGRIGRFLKKGRYAQRVGSGAPVYL

AAVLEYLAAEVLELAGNAARDNKKNRIIPRHVLLAIRNDEELGKLLAGVTIAHGGVLPNI

NPILLPKKTAAKEPKSPSKATKSPKKAK*

>Slycopersicum_ITAG3.2|Solyc09g010400.3|Solyc09g010400.3.1

MEKKGAGGRKGGGPKKKAVTRSVKAGLQFPVGRIGRYLKKGRYSERVGSGAPVYLAAVLE

YLAAEVLELAGNAARDNKKNRIIPRHVLLAVRNDEELGKLLSGVTIAHGGVLPNINPILL

PKKSDKVGKEPAKSPSKATKSPRKA*

>Slycopersicum_ITAG3.2|Solyc11g073260.2|Solyc11g073260.2.1

MESTGKMKKGAAGRRGGGPKKKPVSRSVKAGLQFPVGRIGRFLKKGRYAQRVGSGAPVYL

AAVLEYLAAEVLELAGNAARDNKKNRIIPRHLLLAVRNDEELGKLLAGVTIAHGGVLPNI

NPILLPKKTGGEKAPKSPKKA*

>Slycopersicum_ITAG3.2|Solyc01g099410.3|Solyc01g099410.3.1

MDATKTTKGAGGRKGGPRKKSVTKSIKAGLQFPVGRIGRYLKKGRYAQRVGSGAPIYLAA

VLEYLAAEVLELAGNAARDNKKSRIIPRHVLLAVRNDEELGKLLAGVTIASGGVLPNINP

VLLPKKSAVAEEKSPKAKAGKSPKKA*

>Mguttatus_TOL_v5.0|MgTOL.D1953|MgTOL.D1953.1

MEAAGKPKKGAAGRKIGGTNKKSVTRSARAGLQFPVGRIGRFLKKGRYAQRIGSGAPVYL

AAVLEYLAAEVLELAGNAARDNKKSRIIPRHLLLAVRNDEELGKLLQGVTIAHGGVLPQI

NPVLLPKKTGGDKPIKEPKSPAAKSPKKAAA*

>>Mguttatus_TOL_v5.0|MgTOL.J0362|MgTOL.J0362.1

MEAGGGKAKKGAAGRKAGGPKKKSVSRSTKAGLQFPVGRIGRYLKKGRYSERVGSGAPVY

LAAVLEYLAAEVLELAGNAARDNKKNRITPRHFLLAVRNDEELGKLLAGVTIAYGGVLPN

INPILLPKKNEKVGENAAKSPSKATKSPKKA*

>Mguttatus_TOL_v3.1|MgTOL.L1433|MgTOL.L1433.1

METGGKMKKGAAGRKGTGPKKKPVSRSMKAGLQFPVGRIGRYLKKGRYSQRVGSGAPVYM

AAVLEYLAAEVLELAGNAARDNKKNRIIPRHLLLAVRNDEELGKLLAGVTIAHGGVIPNI

NPVLLPKKSEKAAAAEKSPKSPAKATKSPKKA*

>Mguttatus_v2.0|Migut.J00364|Migut.J00364.1

MEAGGKLKKGAAGRKGGGPKKKSVSRSVKAGLQFPVGRIGRYLKKGRYSQRVGSGAPIYM

AAVLEYLAAEVLELAGNAARDNKKSRIIPRHLLLAVRNDEELGKLLAGVTIAHGGVLPNI

NPILLPKKSEKEVEKAASKSPSKATKSPKKA*

>Hquercifolia_W7

MEAVKTTTKGAGGRRGGERKKSVSKSIKAGLQFPVGRIARFLKKGRYAQRTGTGAPIYLA

AVLEYLAAEVLELAGNAARDNKKNRINPRHVLLAVRNDEELGKLLHGVTIANGGVLPNIN

PVLLPKKSATETDKSSQAKKSPKKA*

>Hquercifolia_v1.1|Hyque.07G027800|Hyque.07G027800.1

MDSGKAKKGAAGRKGGGPKKKPVTRSVRAGLQFPVGRIGRYLKKGRYAQRVGTGAPVYLA

AVLEYLAAEVLELAGNAARDNKKNRIIPRHVLLAVRNDEELGKLLAGVTIAHGGVLPNIN

PVLLPKKSDKAAKEPKSPSKATKSPKKA*

>Hquercifolia_v1.1|Hyque.16G045900|Hyque.16G045900.1

MEGTGKVKKGAGGRKGGGPRKKSVTRSIRAGLQFPVGRIGRYLKKGRYAQRVGTGAPVYM

AAVLEYLAAEVLELAGNAARDNKKNRIIPRHVLLAVRNDEELGKLLSGVTIAHGGVLPNI

NPVLLPKKTDKATKEPKSPSKATKSPKKAA*

>Hquercifolia_v1.1|Hyque.08G145200|Hyque.08G145200.1

MDAGGKVKKGAAGRKGGGPKKKPVTRSVKAGLQFPVGRIGRFLKKGRYAQRVGSGAPVYL

AAVLEYLAAEVLELAGNAARDNKKNRIIPRHVLLAVRNDEELGKLLAGVTIAHGGVLPNI

NPVLLPKKSEKAAKEPKSPSKATKSPKKA*

>Csinensis_v1.1|orange1.1g032326m.g|orange1.1g032326m

MEVTKPTKGAGGRRGGGERKKISKSVKAGLQFPVGRIARFLKKGRYAQRMGSGAPIYMAA

VLEYLAAEVLELAGNAARDNKKNRINPRHVLLAVRNDDELGKLLQGVTIASGGVLPNINP

VLLPKKTTSTSETPKSPKSPKKA*

>Cclementina_v1.0|Ciclev10002795m.g|Ciclev10002795m

MEVTKPTKGAGGRRGGGGRKKISKSVKAGLQFPVGRIARFLKKGRYAQRMGSGAPIYMAA

VLEYLAAEVLELAGNAARDNKKNRINPRHVLLAVRNDDELGKLLQGVTIASGGVLPNINP

VLLPKKTTSTSETPKSPKSPKKA*

>Cclementina_v1.0|Ciclev10013023m.g|Ciclev10013023m

MESGGKVKRGAGGRKGGGPKKKPVSRSVKAGLQFPVGRIGRYLKKGRYSQRVGTGAPVYL

AAVLEYLAAEVLELAGNAARDNKKNRIIPRHVLLAVRNDEELGKLLAGVTIAHGGVLPNI

NPVLLPKKSEKAATKEPKSPAKATKSPKKA*

>Cclementina_v1.0|Ciclev10029498m.g|Ciclev10029498m

MDTPVKVKKGAGGRKGGGPKKQSVSRSVKAGLQFPVGRIGRFLKKGRYSQRVGSGAPVYM

AAVLEYLAAEVLELAGNAARDNKKKKIIPRHVQLAVRNDEELGKLLTGVTIASGGVLPNI

NPVLLPKKTKESVAKEPKSPSKATKSPKKA*

>Ptrifoliata_v1.3.1|Ptrif.0008s2146|Ptrif.0008s2146.1

MDTPVKVKKGAGGRKGGGPKKQSVSRSVKAGLQFPVGRIGRFLKKGRYSQRVGSGAPVYM

AAVLEYLAAEVLELAGNAARDNKKKKIIPRHVQLAVRNDEELGKLLTGVTIASGGVLPNI

NPVLLPKKTNERVAKEPKSPSKATKSPKKA*

>Ptrifoliata_v1.3.1|Ptrif.0006s0583|Ptrif.0006s0583.1

MESGGKVKRGAGGRKGGGPKKKPVSRSVKAGLQFPVGRIGRYLKKGRYSQRVGTGAPVYL

AAVLEYLAAEVLELAGNAARDNKKNRIIPRHVLLAVRNDEELGKLLAGVTIAHGGVLPNI

NPVLLPKKSEKAATKEPKSPAKATKSPKKA*

>Ptrifoliata_v1.3.1|Ptrif.0005s1506|Ptrif.0005s1506.2

MEGTKPTKGAGGRRGGGERKKISKSVKAGLQFPVGRIARFLKKGRYAQRMGSGAPIYMAA

VLEYLAAEVLELAGNAARDNKKNRINPRHVLLAVRNDDELGKLLQGVTIASGGVLPNINP

VLLPKKTTSTSETPKSPKSPKKA*

>Graimondii_v2.1|Gorai.004G004000|Gorai.004G004000.1

METTGKVKKGAGGRKGGGPKKKPVSRSVKAGLQFPVGRIGRYLKKGRYSQRVGTGAPVYL

AAVLEYLAAEVLELAGNAARDNKKNRIIPRHVLLAVRNDEELGKLLAGVTIAHGGVLPNI

NPVLLPKKTEKAAGKEPKSPSKATKSPKKA*

>Graimondii_v2.1|Gorai.004G239900|Gorai.004G239900.1

MESTAKPAGGRKGGVKKKAVSKSVKAGLQFPVGRIARFLKKGRYAQRYGGGAPVYLAAVL

EYLAAEVLELAGNAARDNKKNRINPRHVLLAVRNDEELGKLLQGVTIASGGVLPNINPVL

LPKKTAAAASDSEKAKSKSPKKA*

>Graimondii_v2.1|Gorai.004G004100|Gorai.004G004100.1

METSGKVKKGAGGRKGGGPKKKPVSRSVKAGLQFPVGRIGRYLKKGRYSQRVGTGAPVYL

AAVLEYLAAEVLELAGNAARDNKKNRIIPRHVLLAVRNDEELGKLLAGVTIAHGGVLPNI

NPILLPKKTEKAAGKEPKSPSKATKSPKKA*

>Graimondii_v2.1|Gorai.002G254000|Gorai.002G254000.1

MDAGSKVKKGAGGRKGGGPKKKPVSRSVKAGLQFPVGRIGRYLKKGRYSQRVGTGAPVYL

AAVLEYLAAEVLELAGNAARDNKKNRIIPRHVLLAVRNDEELGKLLAGVTIAHGGVLPNI

NPVLLPKKNEKAAAKEPKSPSKATKSPKKSPKKA*

>Graimondii_v2.1|Gorai.002G254100|Gorai.002G254100.1

MDTGSKVKKGAGGRKGGGPKKKPVSRSVKAGLQFPVGRIGRYLKKGRYSQRVGTGAPVYL

AAVLEYLAAEVLELAGNAARDNKKNRIIPRHVLLAVRNDEELGKLLAGVTIAHGGVLPNI

NPVLLPKKNEKVATKEPKSPSKATKKSPKKA*

>Ghirsutum_v1.1|Gohir.D08G010800|Gohir.D08G010800.1

METSGKVKKGAGGRKGGGPKKKPVSRSVKAGLQFPVGRIGRYLKKGRYSQRVGTGAPVYL

AAVLEYLAAEVLELAGNAARDNKKNRIIPRHVLLAVRNDEELGKLLAGVTIAHGGVLPNI

NPVLLPKKTEKAAGKEPKSPSKATKSPKKA*

>Ghirsutum_v1.1|Gohir.D08G010700|Gohir.D08G010700.1

METTGKVKKGAGGRKGGGPKKKPVSRSVKAGLQFPVGRIGRYLKKGRYSQRVGTGAPVYL

AAVLEYLAAEVLELAGNAARDNKKNRIIPRHVLLAVRNDEELGKLLAGVTIAHGGVLPNI

NPVLLPKKTEKAAGKEPKSPSKATKSPKKA*

>Ghirsutum_v1.1|Gohir.D08G225900|Gohir.D08G225900.1

MESTAKPAGGRKGGVKKKAVSKSVKAGLQFPVGRIARFLKKGRYAQRYGGGAPVYLAAVL

EYLAAEVLELAGNAARDNKKNRINPRHVLLAVRNDEELGKLLQGVTIASGGVLPNINPVL

LPKKTAAAASDSEKAKSKSPKKA*

>Ghirsutum_v1.1|Gohir.D01G208800|Gohir.D01G208800.1

MDAGSKVKKGAGGRKGGGPKKKPVSRSVKAGLQFPVGRIGRYLKKGRYSQRVGTGAPVYL

AAVLEYLAAEVLELAGNAARDNKKNRIIPRHVLLAVRNDEELGKLLAGVTIAHGGVLPNI

NPVLLPKKNEKAAAKEPKSPSKATKSPKKSPKKA*

>Ghirsutum_v1.1|Gohir.D01G208900|Gohir.D01G208900.1

MDTGSKVKKGAGGRKGGGPKKKPVSRSVKAGLQFPVGRIGRYLKKGRYSQRVGTGAPVYL

AAVLEYLAAEVLELAGNAARDNKKNRIIPRHVLLAVRNDEELGKLLAGVTIAHGGVLPNI

NPVLLPKKNEKVATKEPKSPSKATKKSPKKA*

>Ghirsutum_v1.1|Gohir.A08G209000|Gohir.A08G209000.1

MESSAKPAGGRKGGVKKKAVSKSVKAGLQFPVGRIARFLKKGRYAQRYGGGAPVYLAAVL

EYLAAEVLELAGNAARDNKKNRINPRHVLLAVRNDEELGKLLQGVTIASGGVLPNINPVL

LPKKTAAAAASDSEKAKSKSPKKA*

>Gmustelinum_v1.1|Gomus.D08G003600|Gomus.D08G003600.1

METTGKVKKGAGGRKGGGPKKKPVSRSVKAGLQFPVGRIGRYLKKGRYSQRVGTGAPVYL

AAVLEYLAAEVLELAGNAARDNKKNRIIPRHVLLAVRNDEELGKLLAGVTIAHGGVLPNI

NPVLLPKKTEKAAGKEPKSPSKATKSPKKA*

>Gmustelinum_v1.1|Gomus.D08G003700|Gomus.D08G003700.1

METSGKVKKGAGGRKGGGPKKKPVSRSVKAGLQFPVGRIGRYLKKGRYSQRVGTGAPVYL

AAVLEYLAAEVLELAGNAARDNKKNRIIPRHVLLAVRNDEELGKLLAGVTIAHGGVLPNI

NPVLLPKKTEKAAGKEPKSPSKATKSPKKA*

>Gmustelinum_v1.1|Gomus.D08G250500|Gomus.D08G250500.1

MESTAKPAGGRKGGVKKKAVSKSVKAGLQFPVGRIARFLKKGRYAQRYGGGAPVYLAAVL

EYLAAEVLELAGNAARDNKKNRINPRHVLLAVRNDEELGKLLQGVTIASGGVLPNINPVL

LPKKTAAAASDSEKAKSKSPKKA*

>Gmustelinum_v1.1|Gomus.A08G003700|Gomus.A08G003700.1

METSGKVKKGAGGRKGGGPKKKPVSRSVKAGLQFPVGRIGRYLKKGRYSQRVGTGAPVYL

AAVLEYLAAEVLELAGNAARDNKKNRIIPRHVLLAVRNDEELGKLLAGVTIAHGGVLPNI

NPVLLPKKTEKAAGKESKSPSKATKSPRKA*

>Gmustelinum_v1.1|Gomus.A01G237800|Gomus.A01G237800.1

MDTGSKVKKGAGGRKGGGPKKKPVSRSVKAGLQFPVGRIGRYLKKGRYSQRVGTGAPVYL

AAVLEYLAAEVLELAGNAARDNKKNRIIPRHVLLAVRNDEELGKLLAGVTIAHGGVLPNI

NPVLLPKKNEKVATKEPKSPSKATKKSPKKA*

>Gmustelinum_v1.1|Gomus.A01G237500|Gomus.A01G237500.1

MDTGSKVKKGAGGRKGGRPKKKPVSRSVKAGLQFPVGRIGRYLKKGRYSQRVGTGAPVYL

AAVLEYLAAEVLELAGNAARDNKKNRIIPRHVLLAVRNDEELGKLLAGVTIAHGGVLPNI

NPILLPKKNERAAAKEPKSPSKATKKSPKKA*

>Gmustelinum_v1.1|Gomus.A01G237600|Gomus.A01G237600.1

MDAGSKVKKGAGGRKGGGPKKKPVSRSVKAGLQFPVGRIGRYLKKGRYSQRVGTGAPVYL

AAVLEYLAAEVLELAGNAARDNKKNRIIPRHVLLAVRNDEELGKLLAGVTIAHGGVLPNI

NPVLLPKKNEKAAAKEPKSPSKATKSPKKSPKKA*

>Gtomentosum_v1.1|Gotom.A08G003900|Gotom.A08G003900.1

METSGKVKKGAGGRKGGGPKKKPVSRSVKAGLQFPVGRIGRYLKKGRYSQRVGTGAPVYL

AAVLEYLAAEVLELAGNAARDNKKNRIIPRHVLLAVRNDEELGKLLAGVTIAHGGVLPNI

NPVLLPKKTEKAAGKEPKSPSKATKSPKKA*

>Gtomentosum_v1.1|Gotom.A08G244400|Gotom.A08G244400.1

MESSAKPAGGRKGGVKKKAVSKSVKAGLQFPVGRIARFLKKGRYAQRYGGGAPVYLAAVL

EYLAAEVLELAGNAARDNKKNRINPRHVLLAVRNDEELGKLLQGVTIASGGVLPNINPVL

LPKKTAAAAASDSEKAKSKSPKKA*

>Gtomentosum_v1.1|Gotom.D08G258700|Gotom.D08G258700.1

MESTAKPAGGRKGGVKKKAVSKSVKAGLQFPVGRIARFLKKGRYAQRYGGGAPVYLAAVL

EYLAAEVLELAGNAARDNKKNRINPRHVLLAVRNDEELGKLLQGVTIASGGVLPNINPVL

LPKKTAAAASDSEKAKSKSPKKA*

>Gtomentosum_v1.1|Gotom.D08G004200|Gotom.D08G004200.1

METTGKVKKGAGGRKGGGPKKKPVSRSVKAGLQFPVGRIGRYLKKGRYSQRVGTGAPVYL

AAVLEYLAAEVLELAGNAARDNKKNRIIPRHVLLAVRNDEELGKLLAGVTIAHGGVLPNI

NPVLLPKKTEKAAGKEPKSPSKATKSPKKA*

>Gtomentosum_v1.1|Gotom.D01G267100|Gotom.D01G267100.1

MDTGSKVKKGAGGRKGGGPKKKPVSRSVKAGLQFPVGRIGRYLKKGRYSQRVGTGAPVYL

AAVLEYLAAEVLELAGNAARDNKKNRIIPRHVLLAVRNDEELGKLLAGVTIAHGGVLPNI

NPVLLPKKNEKVATKEPKSPSKATKKSPKKA*

>Gbarbadense_v1.1|Gobar.A01G232700|Gobar.A01G232700.1

MDAGSKVKKGAGGRKGGGPKKKPVSRSVKAGLQFPVGRIGRYLKKGRYSQRVGTGAPVYL

AAVLEYLAAEVLELAGNAARDNKKNRIIPRHVLLAVRNDEELGKLLAGVTIAHGGVLPNI

NPVLLPKKNEKAAAKEPKSPSKATKSPKKSPKKA*

>Gbarbadense_v1.1|Gobar.D01G247600|Gobar.D01G247600.1

MDTGSKVKKGAGGRKGGGPKKKPVSRSVKAGLQFPVGRIGRYLKKGRYSQRVGTGAPVYL

AAVLEYLAAEVLELAGNAARDNKKNRIIPRHVLLAVRNDEELGKLLAGVTIAHGGVLPNI

NPVLLPKKNEKVATKEPKSPSKATKKSPKKA*

>Gbarbadense_v1.1|Gobar.D08G003700|Gobar.D08G003700.1

METSGKVKKGAGGRKGGGPKKKPVSRSVKAGLQFPVGRIGRYLKKGRYSQRVGTGAPVYL

AAVLEYLAAEVLELAGNAARDNKKNRIIPRHVLLAVRNDEELGKLLAGVTIAHGGVLPNI

NPVLLPKKTEKAAGKEPKSPSKATKSPKKA*

>Gbarbadense_v1.1|Gobar.D08G003500|Gobar.D08G003500.1

METTGKVKKGAGGRKGGGPKKKPVSRSVKAGLQFPVGRIGRYLKKGRYSQRVGTGAPVYL

AAVLEYLAAEVLELAGNAARDNKKNRIIPRHVLLAVRNDEELGKLLAGVTIAHGGVLPNI

NPVLLPKKTEKAAGKEPKSPSKATKSPKKA*

>Gbarbadense_v1.1|Gobar.D08G247500|Gobar.D08G247500.1

MESTAKPAGGRKGGVKKKAVSKSVKAGLQFPVGRIARFLKKGRYAQRYGGGAPVYLAAVL

EYLAAEVLELAGNAARDNKKNRINPRHVLLAVRNDEELGKLLQGVTIASGGVLPNINPVL

LPKKTAAAASDSEKAKSKSPKKA*

>Ghirsutum_v2.1|Gohir.D01G208900|Gohir.D01G208900.1

MDTGSKVKKGAGGRKGGGPKKKPVSRSVKAGLQFPVGRIGRYLKKGRYSQRVGTGAPVYL

AAVLEYLAAEVLELAGNAARDNKKNRIIPRHVLLAVRNDEELGKLLAGVTIAHGGVLPNI

NPVLLPKKNEKVATKEPKSPSKATKKSPKKA*

>Ghirsutum_v2.1|Gohir.D01G208800|Gohir.D01G208800.1

MDAGSKVKKGAGGRKGGGPKKKPVSRSVKAGLQFPVGRIGRYLKKGRYSQRVGTGAPVYL

AAVLEYLAAEVLELAGNAARDNKKNRIIPRHVLLAVRNDEELGKLLAGVTIAHGGVLPNI

NPVLLPKKNEKAAAKEPKSPSKATKSPKKSPKKA*

>Ghirsutum_v2.1|Gohir.D08G225900|Gohir.D08G225900.1

MESTAKPAGGRKGGVKKKAVSKSVKAGLQFPVGRIARFLKKGRYAQRYGGGAPVYLAAVL

EYLAAEVLELAGNAARDNKKNRINPRHVLLAVRNDEELGKLLQGVTIASGGVLPNINPVL

LPKKTAAAASDSEKAKSKSPKKA*

>Ghirsutum_v2.1|Gohir.D08G010700|Gohir.D08G010700.1

METTGKVKKGAGGRKGGGPKKKPVSRSVKAGLQFPVGRIGRYLKKGRYSQRVGTGAPVYL

AAVLEYLAAEVLELAGNAARDNKKNRIIPRHVLLAVRNDEELGKLLAGVTIAHGGVLPNI

NPVLLPKKTEKAAGKEPKSPSKATKSPKKA*

>Ghirsutum_v2.1|Gohir.A08G209000|Gohir.A08G209000.1

MESSAKPAGGRKGGVKKKAVSKSVKAGLQFPVGRIARFLKKGRYAQRYGGGAPVYLAAVL

EYLAAEVLELAGNAARDNKKNRINPRHVLLAVRNDEELGKLLQGVTIASGGVLPNINPVL

LPKKTAAAAASDSEKAKSKSPKKA*

>Gdarwinii_v1.1|Godar.A01G250600|Godar.A01G250600.1

MDAGSKVKKGAGGRKGGGPKKKPVSRSVKAGLQFPVGRIGRYLKKGRYSQRVGTGAPVYL

AAVLEYLAAEVLELAGNAARDNKKNRIIPRHVLLAVRNDEELGKLLAGVTIAHGGVLPNI

NPVLLPKKNEKAAAKEPKSPSKATKSPKKSPKKA*

>Gdarwinii_v1.1|Godar.D08G004100|Godar.D08G004100.1

METTGKVKKGAGGRKGGGPKKKPVSRSVKAGLQFPVGRIGRYLKKGRYSQRVGTGAPVYL

AAVLEYLAAEVLELAGNAARDNKKNRIIPRHVLLAVRNDEELGKLLAGVTIAHGGVLPNI

NPVLLPKKTEKAAGKEPKSPSKATKSPKKA*

>Gdarwinii_v1.1|Godar.D08G004200|Godar.D08G004200.1

METSGKVKKGAGGRKGGGPKKKPVSRSVKAGLQFPVGRIGRYLKKGRYSQRVGTGAPVYL

AAVLEYLAAEVLELAGNAARDNKKNRIIPRHVLLAVRNDEELGKLLAGVTIAHGGVLPNI

NPVLLPKKTEKAAGKEPKSPSKATKSPKKA*

>Gdarwinii_v1.1|Godar.A08G244700|Godar.A08G244700.1

MESSAKPAGGRKGGVKKKAVSKSVKAGLQFPVGRIARFLKKGRYAQRYGGGAPVYLAAVL

EYLAAEVLELAGNAARDNKKNRINPRHVLLAVRNDEELGKLLQGVTIASGGVLPNINPVL

LPKKTAAAAASDSEKAKSKSPKKA*

>Ppersica_v2.1|Prupe.6G322000|Prupe.6G322000.1

MEAAKVTKGAGGRKGGERKKSVSKSVKAGLQFPVGRIARFLKKGRYAQRTGTGAPIYLAA

VLEYLAAEVLELAGNAARDNKKTRINPRHVLLAVRNDEELGKLLQGVTIASGGVLPNINP

VLLPKKTSNASSEAAEKAPKSPKSPKSPKKA*

>Ppersica_v2.1|Prupe.6G082300|Prupe.6G082300.1

METGGKVKKGAGGRKGGGPKKKPVTRSVKAGLQFPVGRIGRYLKKGRYAQRVGSGAPVYL

AAVLEYLAAEVLELAGNAARDNKKNRIIPRHLLLAVRNDEELGKLLAGVTIAHGGVLPNI

NPVLLPKKSEKAAAKEPKSPSKATKSPKKA*

>Vunguiculata_v1.1|Vigun05g256500|Vigun05g256500.1

MDAGGKIKKGAGGRKGGGPKKKPVARSVRAGLQFPVGRIGRYLKKGRYSQRVGTGAPVYL

AAVLEYLAAEVLELAGNAARDNKKNRIIPRHVLLAVRNDEELGKLLAGVTIAHGGVLPNI

NPVLLPKKTEKASKEPKSPSKATKSPKKA*

>Gmax_Wm82.a4.v1|Glyma.13G333900|Glyma.13G333900.1

MDTGGKIKKGAGGRKGGGPKKKPVSRSVKAGLQFPVGRIGRYLKKGRYAQRVGTGAPVYL

AAVLEYLAAEVLELAGNAARDNKKNRIIPRHVLLAVRNDEELGKLLAGVTIAHGGVLPNI

NPVLLPKKTERASKEPKSPSKATKSPKKS*

>Gmax_Wm82.a4.v1|Glyma.13G334300|Glyma.13G334300.1

MDAGGKIKKGAGGRKGGGPKKKPVSRSVKAGLQFPVGRIGRYLKKGRYAQRVGTGAPVYL

AAVLEYLAAEVLELAGNAARDNKKNRIIPRHVLLAVRNDEELGKLLAGVTIAHGGVLPNI

NPVLLPKKTERAAKEPKSPSKATKSPKKA*

>Gmax_W7

MDAGGKIKKGAGGRKGGGPKKKPVSRSVKAGLQFPVGRIGRYLKKGRYAQRVGTGAPVYL

AAVLEYLAAEVLELAGNAARDNKKNRIIPRHVLLAVRNDEELGKLLAGVTIAHGGVLPNI

NPVLLPKKTQVAKEPKSPSKATKSPKKA*

>Gsoja_v1.1|GlysoPI483463.15G037800|GlysoPI483463.15G037800.1

MDADGKIKKGAGGRKGGGPKKKPVSRSVKAGLQFPVGRIGRYLKKGRYAQRVGTGAPVYL

AAVLEYLAAEVLELAGNAARDNKKNRIIPRHVLLAVRNDEELGKLLAGVTIAHGGVLPNI

NPVLLPKKSERASKEPKSPSKATKSPKKA*

>Gsoja_v1.1|GlysoPI483463.15G037900|GlysoPI483463.15G037900.1

MDAGGKIKKGAGGRKGGGPKKKPVSRSVKAGLQFPVGRIGRYLKKGRYAQRVGTGAPVYL

AAVLEYLAAEVLELAGNAARDNKKNRIIPRHVLLAVRNDEELGKLLAGVTIAHGGVLPNI

NPVLLPKKTERASKEPKSPSKATKSPKKA*

>Gsoja_v1.1|GlysoPI483463.13G283200|GlysoPI483463.13G283200.1

MDTGGKIKKGAGGRKGGGPKKKPVSRSVKAGLQFPVGRIGRYLKKGRYAQRVGTGAPVYL

AAVLEYLAAEVLELAGNAARDNKKNRIIPRHVLLAVRNDEELGKLLAGVTIAHGGVLPNI

NPVLLPKKTERASKEPKSPSKATKSPKKS*

>Gmax_Lee_v1.1|GlymaLee.15G037900|GlymaLee.15G037900.1

MDADGKIKKGAGGRKGGGPKKKPVSRSVKAGLQFPVGRIGRYLKKGRYAQRVGTGAPVYL

AAVLEYLAAEVLELAGNAARDNKKNRIIPRHVLLAVRNDEELGKLLAGVTIAHGGVLPNI

NPVLLPKKSERASKEPKSPSKATKSPKKA*

>Vunguiculata_v1.2|Vigun05g256500|Vigun05g256500.1

MDAGGKIKKGAGGRKGGGPKKKPVARSVRAGLQFPVGRIGRYLKKGRYSQRVGTGAPVYL

AAVLEYLAAEVLELAGNAARDNKKNRIIPRHVLLAVRNDEELGKLLAGVTIAHGGVLPNI

NPVLLPKKTEKASKEPKSPSKATKSPKKA*

>Vunguiculata_Suvita2_v1.1|VuSuvita2.05G245800|VuSuvita2.05G245800.1

MDTGGKIKKGAGGRKGGGPKKKPVTRSVRAGLQFPVGRIGRYLKKGRYAQRVGTGAPVYL

AAVLEYLAAEVLELAGNAARDNKKNRIIPRHVLLAVRNDEELGKLLAGVTIAHGGVLPNI

NPVLLPKKTEKASKEPKSPSKATKSPKKA*

>Cillinoinensis_lakota_v1.1|CiLak.13G097500|CiLak.13G097500.1

MEKKGAGGRRGGGGPKKKPVSRSVKAGLQFPVGRIGRYLKKGRYSQRVGTGAPVYMAAVL

EYLAAEVLELAGNAARDNKKNRIIPRHVLLAVRNDEELGKLLAGVTIAHGGVLPNINPVL

LPKKSDKATKEPKSPSKATKSPKKA*

>Cillinoinensis_v1.1|Caril.08G161000|Caril.08G161000.1

MEAAKTTTRGAGGRRGGDRKKSVSKSIKAGLQFPVGRIARYLKKGRYAQRTGTGAPIYLA

AVLEYLAAEVLELAGNAARDNKKNRINPRHVLLAVRNDEELGKLLQGVTIANGGVLPNIN

PVLLPKKTATSESEKVSKSPKKA*

>Cillinoinensis_v1.1|Caril.07G020100|Caril.07G020100.1

MDAAKTTRGAGGRRGGDRKKSVSKSIKAGLQFPVGRIARYLKKGRYAQRTGSGAPIYLAA

VLEYLAAEVLELAGNAARDNKKNRINPRHVLLAVRNDEELGKLLQGVTIASGGVLPNINP

ILLPKKTTTESDKAPKSPKKTPKKA*

>Pacutifolius_v1.0|Phacu.CVR.005G174600.1|Phacu.CVR.005G174600.1

MDTTGKFKKGAGGRKGGGPKKKAVSRSVKAGLQFPVGRIGRFLKKGRYAQRVGAGAPVYL

AAVLEYLAAEVLELAGNAARDNKKNRIIPRHVLLAVRNDEELGKLLSGVTIAHGGVLPNI

NPVLLPKKSEKASKEPKSPSKATKSPKKA*

>Pacutifolius_v1.0|Phacu.CVR.005G175000.1|Phacu.CVR.005G175000.1

METGGKIKKGAGGRKGGGPKKKPVTRSVRAGLQFPVGRVGRYLKKGRYAQRVGTGAPVYL

AAVLEYLAAEVLELAGNAARDNKKNRIIPRHVLLAVRNDEELGKLLSGVTIAHGGVLPNI

NPVLLPKKTDKASKEPKSPSKATKSPKKA*

>Cillinoinensis_Pawnee_v1.1|CiPaw.08G162800|CiPaw.08G162800.1

MEAAKTTRGAGGRRGGDRKKSVSKSIKAGLQFPVGRIARYLKKGRYAQRTGTGAPIYLAA

VLEYLAAEVLELAGNAARDNKKNRINPRHVLLAVRNDEELGKLLQGVTIANGGVLPNINP

VLLPKKTATSESEKVSKSPKKA*

>Cdentata_v1.1|Caden.08G050200|Caden.08G050200.1

MDKKGAGGRKGSGSRKKSVPRSVKAGLQFPVGRIGRYLKKGRYAKRFGSGAPVYMAAVLE

YLAAEVLELAGNAARDNKKTRIIPRHVLLAVRNDEELGKLLAGVTIAHGGVLPNINPVLL

PKKTEKATKEPKSPSKATKSPKKA*

>Cdentata_v1.1|Caden.09G008800|Caden.09G008800.1

MDTGGKVKRGAGGRKGGGPKKKPVSRSVKAGLQFPVGRIGRYLKKGRYAQRVGTGAPVYL

AAVLEYLAAEVLELAGNAARDNKKNRIIPRHVLLAVRNDEELGKLLAGVTIAHGGVLPNI

NPVLLPKKTEKAATKEPKSPARATKSPKKA*

>Gmax_W7_2

MDAGGKIKKGAGGRKGGGPKKKPVSRSVKAGLQFPVGRIGRYLKKGRYAQRVGTGAPVYL

AAVLEYLAAEVLELAGNAARDNKKNRIIPRHVLLAVRNDEELGKLLAGVTIAHGGVLPNI

NPVLLPKKTQVAKEPKSPSKATKSPKKA*

>Gmax_Fiskeby_v1.1|GlymaFiskIII.13G315200|GlymaFiskIII.13G315200.1

MDTGGKIKKGAGGRKGGGPKKKPVSRSVKAGLQFPVGRIGRYLKKGRYAQRVGTGAPVYL

AAVLEYLAAEVLELAGNAARDNKKNRIIPRHVLLAVRNDEELGKLLAGVTIAHGGVLPNI

NPVLLPKKTERASKEPKSPSKATKSPKKS*

>Gmax_Fiskeby_v1.1|GlymaFiskIII.15G039100|GlymaFiskIII.15G039100.1

MDADGKIKKGAGGRKGGGPKKKPVSRSVKAGLQFPVGRIGRYLKKGRYAQRVGTGAPVYL

AAVLEYLAAEVLELAGNAARDNKKNRIIPRHVLLAVRNDEELGKLLAGVTIAHGGVLPNI

NPVLLPKKSERASKEPKSPSKATKSPKKA*

>Egrandis_W7

MEARGAGGRRGERKKSVSKSVKAGLQFPVGRVARFLKKGRYARRTGTGAPVYLAAVLEYL

AAEVLELAGNAARDNKKNRISPRHVLLAVRNDEELGKLLQGVTIASGGVLPNINPVLLPK

KASSDAPKASQPKSPAKA*

>Egrandis_v2.0|Eucgr.A01241|Eucgr.A01241.1

METGGKLKKGAGGRKGGGPKKKPVSRSVKAGLQFPVGRIGRYLKKGRYSQRVGTGAPVYL

AAVLEYLAAEVLELAGNAARDNKKNRIIPRHVLLAVRNDEELGKLLAGVTIAHGGVLPNI

NPVLLPKKTEKATKEPKSPSKATKSPKKA*

>Egrandis_v2.0|Eucgr.K03160|Eucgr.K03160.1

MESLGKPKKGAGGRKGGGPKKKPVSRSVKAGLQFPVGRIGRYLKKGRYSQRVGTGAPVYL

AAVLEYLAAEVLELAGNAARDNKKNRIIPRHVLLAVRNDEELGKLLAGVTIAHGGVLPNI

NPVLLPKKTEKATKEPKSPSKATKSPKKA*

>Vvinifera_v2.1|VIT_214s0060g02360|VIT_214s0060g02360.1

MENTKPTKGAGGRKGGERKKSVSKSVKAGLQFPVGRIARFLKTGRYAQRTGTGAPIYLAA

VLEYLAAEVLELAGNAARDNKKNRISPRHVLLAVRNDEELGKLLRGVTIANGGVLPNINP

VLLPKKSNSEPKSPKKA*

>Vvinifera_v2.1|VIT_208s0040g03300|VIT_208s0040g03300.1

MEAAGKVKKGAGGRKGGGPKKKPVSRSVKAGLQFPVGRIGRYLKKGRYSQRVGTGAPVYL

AAVLEYLAAEVLELAGNAARDNKKNRIIPRHVLLAVRNDEELGKLLAGVTIAHGGVLPNI

NPVLLPKKSDKAAKEPKSPSKATKSPRKT*

>Vvinifera_v2.1|VIT_200s0753g00020|VIT_200s0753g00020.1

MEKAKTELGGALSGTVKKGVGGRKGSGPKKKPLSYPVKASLQFPVGRIGRYLKKGHYSQR

VGTGASNYLAAVLEYQASEVLELAGNKHRIIPRHVLLVVRNDEELGKLLSGVTIAHGGFL

PNINPVLLPKKTDKAAKEPKSPSKATKSPRKA*

>Vvinifera_v2.1|VIT_206s0004g04270|VIT_206s0004g04270.1

MESTGKVKKGAGGRKGGGPKKKPVSRSVKAGLQFPVGRIGRYLKKGRYSQRVGTGAPVYL

AAVLEYLAAEVLELAGNAARDNKKNRIIPRHVLLAVRNDEELGKLLSGVTIAHGGVLPNI

NPVLLPKKTDKATKEPKSPSKATKSPKKA*

>Ljaponicus_Lj1.0v1|Lj3g0027292|Lj3g0027292.1

MDSTATGKVKKGAAGRKAGGPKKKSVSRSVKAGLQFPVGRIGRYLKKGRYAQRVGSGAPV

YLAAVLEYLAAEVLELAGNAARDNKKNRIIPRHVLLAVRNDEELGKLLAGVTIAHGGVLP

NINPVLLPKKTAEKAPKEPKSPAKKAAKSPKKAAA*

>Ljaponicus_Lj1.0v1|Lj3g0000114|Lj3g0000114.1

MDTTGKVKKGAGGRKGGGPKKKPVSKSVEVGLQFPVGRIGQTGAPVYLATVLEYLPVKVL

ELVGNAARDSKKNRIIPRHVLLAVRNDEELGKLLAGVTIAHGGVLPNINPVLLPKKTAEK

AAQEPKSLSKKAAKSPKKA*

>Ljaponicus_Lj1.0v1|Lj3g0001339|Lj3g0001339.1

MDTGGKSKKGAGGRKAGGPKKKPVSRSVKAGLQFPVGRIGRYLKKGRYAQRVGTGAPVYL

AAVLEYLAAEVLELAGNAARDNKKNRIIPRHVLLAVRNDEELGKLLAGVTIAHGGVLPNI

NPVLLPKKTAEKAPKESKSPPKKAGKSPKKA*

>Ljaponicus_Lj1.0v1|Lj3g0011571|Lj3g0011571.1

MDTTTGKAKKGAGGRKIGGPKKKPVSRSIKAGLQFPVGRIGRYLKKGRYAQRVGTGAPVY

LAAVLEYLAAEVLELAGNAARDNKKNRIIPRHVLLAVRNDEELGKLLAGVTIAHGGVLPN

INPVLLPKKTAEKVAKEPKSPAKKGVKSPKKA*

>Fvesca_v4.0.a2|FvH4_6g06430|FvH4_6g06430.t1

MESGKVTRGAGGRKGGDRKKSVSKSVKAGLQFPVGRIARFLKKGRYAQRTGSGAPIYLAA

VLEYLAAEVLELAGNAARDNKKTRINPRHVQLAVRNDEELGKLLAGVTIASGGVLPNINP

VLLPKKTGHSDASEKATKSPKSPKKAAA*

>Fvesca_v4.0.a2|FvH4_6g14320|FvH4_6g14320.t1

METGGKFKKGAGGRKAGGPKKKPVTRSTKAGLQFPVGRIGRYLKNGRYAQRVGSGAPVYL

AAVLEYLAAEVLELAGNAARDNKKNRIIPRHVLLAVRNDEELGKLLAGVTIAHGGVLPNI

NPVLLPKNSGKTAAKEPKSPSKATKSPKKAA*

>Fvesca_v4.0.a2|FvH4_3g35330|FvH4_3g35330.t1

METGGGKIKKGAAGRKGGGPRKKSVTRSIKAGLQFPVGRIGRYLKKGRYAQRVGSGAPVY

LAAVLEYLAAEVLELAGNAARDNKKTRIIPRHLLLAIRNDEELGKLLSGVTIAHGGVLPN

INPILLPKKAEKAGGGVTKEPKSPSKAGKSPKKAAA*

>Csativus_v1.0|Cucsa.065980|Cucsa.065980.1

MDSGGKAKKGFAGRRGGADSKKKPVSRSVKAGLQFPVGRIGRYLKNGRYSQRVGTGAPVY

LAAVLEYLAAEVLELAGNAARDNKKNRIIPRHVLLAVRNDEELGKLLAGVTIAHGGVLPN

INPVLLPKKSERVAAAKEPKSPSKGTKSPKKA*

>Csativus_v1.0|Cucsa.091130|Cucsa.091130.1

MEGTKGAGGRKGGDRTKVSKSVKAGLQFPVGRIGRYLKKGRYAQRTAAGAPIYLAAVLEY

LAAEVLELAGNAARDNKKNRINPRHVLLAVRNDEELGKLLQGVTIASGGVLPNINPVLLP

KKTASNSTPTAEKAQKSPKKA*

>Csativus_v1.0|Cucsa.213450|Cucsa.213450.1

METGGKLKKGAGGRKGGGPKKKPVSRSVKAGLQFPVGRIGRYLKNGRYARRVGTGAPVYL

AAVLEYLAAEVLELAGNAARDNKKNRIIPRHVLLAIRNDEELGKLLAGVTIAHGGVLPNI

NPVLLPKKSEKATTKEPKSPSKAPKATKSPKKA*

>Mtruncatula_Mt4.0v1|Medtr2g096570|Medtr2g096570.1

MDASTKTKKGAGGRKGGGPRKKSVTRSIRAGLQFPVGRIGRYLKKGRYAQRVGTGAPVYL

AAVLEYLAAEVLELAGNAARDNKKNRIIPRHVLLAVRNDEELGKLLAGVTIAHGGVLPNI

NPVLLPKKTERSNTVSKEPKSPKPKAGKSPKKA*

>Mtruncatula_Mt4.0v1|Medtr2g096610|Medtr2g096610.1

MDASTKTKKGAGGRKGGPRKKSVTRSTRAGLQFPVGRIGRYLKKGRYAQRVGTGAPVYLA

AVLEYLAAEVLELAGNAARDNKKNRIIPRHVLLAVRNDEELGKLLAGVTIAHGGVLPNIN

PILLPKKNEKAATTTKSPSKATKSPKKA*

>Mtruncatula_Mt4.0v1|Medtr8g086640|Medtr8g086640.1

MDASTKATKKGAGGRKGGGPRKKSVTRSIRAGLQFPVGRIGRYLKKGRYAQRVGTGAPVY

LAAVLEYLAAEVLELAGNAARDNKKNRIIPRHVLLAVRNDEELGKLLAGVTIAHGGVLPN

INPVLLPKKSEKPVKEPKSPSKAKKSPKKA*

>Mtruncatula_Mt4.0v1|Medtr4g063280|Medtr4g063280.1

MDKKGAGGRKGGEPRKKSVTRSIRAGLQFPVGRIGRYLKKGRYAQRVGTGAPVYLAAVLE

YLAAEVLELAGNAARDNKKNRIIPRHVLLAVRNDEELGKLLNGVTIAHGGVFPNINPILL

PKKTEKAVSKEPKSPKKAGKSPKKA*

>Mtruncatula_Mt4.0v1|Medtr4g071150|Medtr4g071150.1

MDASTKTTKKGAGGRKGGGPRKKSVTRSIRAGLQFPVGRIGRYLKKGRYAQRVGTGAPVY

LAAVLEYLAAEVLELAGNAARDNKKNRIIPRHLLLAVRNDEELGKLLAGVTIAHGGVLPN

INPILLPKKTERANTGGKEPKTTKAGKSPKKA*

>Mtruncatula_Mt4.0v1|Medtr4g064005|Medtr4g064005.1

MEASTKTTKKGAGGRKGGGPRKKSVTRSIRAGLQFPVGRIGRYLKKGRYAQRVGTGAPVY

LAAVLEYLAAEVLELAGNAARDNKKNRIIPRHVLLAVRNDEELGKLLNGVTIAHGGVLPN

INPILLPKKNEKTDTKEPKKAGKSPKKA*

>Mtruncatula_Mt4.0v1|Medtr4g063410|Medtr4g063410.1

MEASTKTTKKGAGGRKGGGPRKKSVTRSIRAGLQFPVGRIGRYLKKGRYAQRVGTGAPVY

LAAVLEYLAAEVLELAGNAARDNKKNRIIPRHVLLAVRNDEDLGKLLNGVTIAHGGVLPN

INPILLPKKIENSVSKEPKKAGKSPKKA*

>Mtruncatula_Mt4.0v1|Medtr4g064967|Medtr4g064967.1

MDASPKATKKGAGGRKGGGPRKKSVTRSIRAGLQFPVGRIGRYLKKGRYAQRVGTGAPVY

LAAVLEYLAAEVLELAGNAARDNKKNRIIPRHVLLAVRNDEELGKLLNGVTIAHGGVLPN

INPILLPKKNEKAVPKEPKKAGKSPKKA*

>Tpratense_v2|Tp57577_TGAC_v2_gene34291|Tp57577_TGAC_v2_mRNA35454

MDTSPKSKKGAGGRKGGGPRKKSVTRSTRAGLQFPVGRIGRYLKKGRYAQRVGTGAPVYL

AAVLEYLAAEVLELAGNAARDNKKNRIIPRHVLLAVRNDEELGKLLAGVTIAHGGVLPNI

NPILLPKKTDRSTTASKEPKSPKAKKSPKKA*

>Tpratense_v2|Tp57577_TGAC_v2_gene34327|Tp57577_TGAC_v2_mRNA35494

MDSTTNTKTKKGAAGRKGGGPRKKSVTRSTRAGLQFPVGRIGRYLKKGRYAQRVGTGAPV

YLAAVLEYLAAEVLELAGNAARDNKKNRIIPRHVLLAVRNDEELGKLLAGVTIAHGGVLP

NINPILLPKKTDRSTTASKEPKSPKAGKSPKKA*

>Tpratense_v2|Tp57577_TGAC_v2_gene34329|Tp57577_TGAC_v2_mRNA35496

MDASTKTKKGAGGRKGGGPRKKSVTRSTRAGLQFPVGRIGRYLKKGRYAQRVGTGAPVYL

AAVLEYLAAEVLELAGNAARDNKKNRIIPRHVLLAVRNDEELGKLLAGVTIAHGGVLPNI

NPILLPKKTDRSTTASKEPKSPKAKKSPKKA*

>Pvulgaris_v2.1|Phvul.007G118400|Phvul.007G118400.1

MEADGKIKKSVGGRKGGGQTKKPVTRSVRAGLQFPVGRVGRYLKKGRYAKCVGTGALVYL

VVVLEYLAVEVLELAGNAARDNKKNMIIPRHVLLAVRNDEELGKLLFGVTIAHGGVLPNI

NPVLLPKKTDKASKEPKSPSKATKSPKKA*

>Pvulgaris_v2.1|Phvul.L003543|Phvul.L003543.1

METTGKIKKGAGGRKGGGPKKKPVSRSVKAGLQFPVGRIGRYLKKGRYAQRVGSGAPIYL

AAVLEYLAAEVLELAGNAARDNKKNRIIPRHVLLAVRNDEELGKLLAGVTIAHGGVLPNI

NPVLLPKKTEKASKEPKSPSKATKSPRKS*

>Pvulgaris_v2.1|Phvul.L003343|Phvul.L003343.1

MDTTGKIKKGAGGRKGGGPKKKAVSRSVKAGLQFPVGRIGRFLKKGRYAQRVGSGAPVYL

AAVLEYLAAEVLELAGNAARDNKKNRIIPRHVLLAVRNDEELGKLLSGVTIAHGGVLPNI

NPVLLPKKSDKASKEPKSPSKATKSPRKA*

>Pvulgaris_v2.1|Phvul.L003843|Phvul.L003843.1

METGGKIKKGAGGRKGGGPKKKPVTRSVRAGLQFPVGRVGRYLKKGRYAKRVGTGAPVYL

AAVLEYLAAEVLELAGNAARDNKKNRIIPRHVLLAVRNDEELGKLLSGVTIAHGGVLPNI

NPILLPKKTDKAVKEPKSPSKATKSPKKA*

>Mdomestica_v1.1|MD03G1098100|MD03G1098100

METGGKLKKGAGGRKGGGPKKKAVTRSVRAGLQFPVGRIGRYLKKGRYAQRVGSGAPVYL

AAVLEYLAAEVLELAGNAARDNKKNRIIPRHLLLAVRNDEELGKLLAGVTIAHGGVLPNI

NPVLLPKKSEKAAGKEPKSPTKATKSPKKA*

>Mdomestica_W7

METTKVTKGAGGRRGGERKKSVSKSVKAGLQFPVGRIARFLKKGRYAQRTGTGAPIYLAA

VLEYLAAEVLELAGNAARDNKKTRINPRHVLLAVRNDEELGKLLAGVTIASGGVLPNINP

VLLPKKTGSQEGEKAPKSPKAAKSPKKA*

>Mdomestica_v1.1|MD12G1138300|MD12G1138300

METGGKFKKGAGGRRAGGPKKKPVTRSVRAGLQFPVGRIGRYLKHGRYAQRIGTGAPVYL

AAVLEYLAAEVLELAGNAARDNKKNRIIPRHVLLAVRNDEELGKLLKGVTIAHGGVLPNI

NPVLLPKKHERVAKEPKSPVKAATKSPKKA*

>Ahypogaea_v1.0|arahy.Tifrunner.gnm1.ann1.SJW8XV|arahy.Tifrunner.gnm1.ann1.SJW8XV.1

MDAASKVKKGAGGRKGGGPKKKPVSRSVKAGLQFPVGRIGRYLKKGRYSQRVGTGAPVYL

AAVLEYLAAEVLELAGNAARDNKKNRIIPRHVLLAVRNDEELGKLLAGVTIAHGGVLPNI

NPVLLPKKTAEKAPKETKSPSKATKSPKKA*

>Ahypogaea_v1.0|arahy.Tifrunner.gnm1.ann1.DF4V8K|arahy.Tifrunner.gnm1.ann1.DF4V8K.1

MDSPAGKTKKGAAGRRGGGPKRKPVSRSVKAGLQFPVGRIGRFLKKGRYSQRVGTGAPVY

LAAVLEYLAAEVLELAGNAARDNKKNRIIPRHVLLAVRNDEELGKLLAGVTIAHGGVLPN

INPVLLPKKSQTATKEPKSPSKGTKSPKKA*

>Ahypogaea_W7

MDSPAGKTKKGAAGRRGGGPKRKPVSRSVKAGLQFPVGRIGRYLKKGRYSQRVGTGAPVY

LAAVLEYLAAEVLELAGNAARDNKKNRIIPRHVLLAVRNDEELGKLLAGVTIAHGGVLPN

INPVLLPKKSQTATKEPKSPSKGTKSPKKA*

>Plunatus_V1|Pl05G0000253600.v1|Pl05G0000253600.1.v1

MEAASKIKKGAGGRKGGGPKKKPVSRSVKAGLQFPVGRIGRYLKKGRYAQRVGSGAPIYL

AAVLEYLAAEVLELAGNAARDNKKNRIIPRHVLLAVRNDEELGKLLAGVTIAHGGVLPNI

NPVLLPKKTEKASKEPKSPSKATKSPRKS*

>Plunatus_V1|Pl05G0000253500.v1|Pl05G0000253500.1.v1

MDTTGKIKKGAGGRKGGGPKKKAVSRSVKAGLQFPVGRIGRFLKKGRYSQRVGAGAPVYL

AAVLEYLAAEVLELAGNAARDNKKNRIIPRHVLLAVRNDEELGKLLSGVTIAHGGVLPNI

NPVLLPKKSEKASKELKSLSRATKSPRKA*

>Plunatus_V1|Pl05G0000253900.v1|Pl05G0000253900.1.v1

METGGKIKKGAGGRKGGGPKKKPVTRSVRAGLQFPVGRVGRYLKKGRYAKRVGTGAPVYL

AAVLEYLAAEVLELAGNAARDNKKNRIIPRHVLLAVRNDEELGKLLSGVTIAHGGVLPNI

NPVLLPKKTDKASKEPKSPSKATKSPKKA*

>Lalbus_v1|Lalb_Chr12g0205671|Lalb_Chr12g0205671

MDTGKSKKGAAGRKGGGPRKKAVSRSVRAGLQFPVGRIGRYLKKGRYSQRVGTGAPVYLA

AVLEYLAAEVLELAGNAARDNKKNRIIPRHVLLAVRNDEELGKLLAGVTIAHGGVIPNIN

PVLLPKKTGAAAGASTSKEPKSPTKAAKAPAKAGKSPAKGAKSPKKAA*

>Lalbus_v1|Lalb_Chr01g0003771|Lalb_Chr01g0003771

MDATSGKSKKGAGGRKGGGPRKKSVTRSIRAGLQFPVGRIGRYLKKGRYAQRVGTGAPVY

LAAVLEYLAAEVLELAGNAARDNKKNRIIPRHVLLAVRNDEELGKLLAGVTIAHGGVIPN

INPVLLPKKNEKTVAAKEPKSPSKATKSPRKAT*

>Lalbus_v1|Lalb_Chr07g0186851|Lalb_Chr07g0186851

MDAGAGKSKKGAAGRKGGGPRKKSVTRSIRAGLQFPVGRIGRYLKKGRYAQRVGTGAPVY

LAAVLEYLAAEVLELAGNAARDNKKNRIIPRHVLLAVRNDEELGKLLAGVTIAHGGVLPN

INPVLLPKKTGASTSSAKEPKSPSKAAKSPKKAA*

>Lalbus_v1|Lalb_Chr19g0137721|Lalb_Chr19g0137721

MDTVTGKSKKGAGGRKGGGPRKKSVTRSVKAGLQFPVGRIGRYLKKGRYAQRVGNGAPVY

LAAVLEYLAAEVLELAGNAARDNKKNRIIPRHLLLAVRNDEELGKLLAGVTIAHGGVLPN

INPVLLPKKNDKVASGKEPKSPSKATKSPKKA*

>Atrichopoda_W7

MDPASKTKKGAGGRKGGPKKKPTSKSVKAGLQFPVGRITRYLKKGRYSQRVGIGAPVYLA

AVLEYLAAEVLELAGNAARDNKKSRIIPRHVLLAVRNDDELGKLLAGVTIPHGGVLPNIN

PVLLPKRSHKPETQEPKTPKSPAKKAA*

>Atrichopoda_v1.0|evm_27.TU.AmTr_v1.0_scaffold00057.272|evm_27.model.AmTr_v1.0_scaffold00057.272

MESGKAAKSVGGRKGGVKKKSVSKSVKAGLQFPVGRIARFLKKGRYAQRVGTGAPVYLAA

VLEYLAAEVLELAGNAARDNKKNRIIPRHLLLAVRNDEELGKLLAGVTIASGGVLPNIHS

VLLPKKTEKSGEPKSPKKTTAKSPKKL*

>Acoerulea_W7

METPTKVVGKGGRTGESRKKAVSKSTKAGLQFPVGRVARYLKKGRYAQRVGIGAPIYLTA

VMEYLAAEVLELAGNAARDNKKSRINPRHVLLAVRNDEELAKLLAGVTIAYGGVLPNIHS

VLLPKKTENNKSQQKTTTSSQPTTSSSPTSKPADQA*

>Acoerulea_v3.1|Aqcoe2G179800|Aqcoe2G179800.1

MEGTGKSRKGAAGRKAGGPKKKSVSRSVKAGLQFPVGRLARYLKAGRYARRVGTGAPVYL

AAVLEYLAAEVLELAGNAARDNKKRTINPRHILLAVRNDEELGKLLSGVTIAYGGVLPNI

NPVLLPKKSEKLKEAKSPTKSPKKAAV*

>Acoerulea_v3.1|Aqcoe1G470800|Aqcoe1G470800.1

MEATGGKTKKGAAGRKAGGPKKKSVSRSVKAGLQFPVGRLGRYLKKGRYAQRVGSGAPVY

LAAVLEYLAAEVLELAGNAARDNKKNRIIPRHVLLAIRNDEELGKLLSGVTIAHGGVLPN

INPVLLPKKSEKVANKEPKSPSKTATKSPKKA*

>Ckanehirae_v3|CKAN_02719100|CKAN_02719100

MKFPIEREMEATETIKPAGGRKGSARKKAVSKSLKSGLQFPVGIILRFLKKGRYAQRLGF

GAPIYLAAVLEYLVVEVLELAGNAARDNKKIRIIPRHVLLAVRNDEELGKLLHGVTIAHG

GVLPNIHSVLLPKKTDKGNAEATKAPKSPKKD*

>Ckanehirae_v3|CKAN_02479900|CKAN_02479900

METAGKVKKGAAGRKGGGPKKKPVSRSVKAGLQFPVGRIGRYLKKGRYSQRVGTGAPVYL

AAVLEYLAAEVLELAGNAARDNKKNRIIPRHVLLAVRNDEELGRLLAGVTIAHGGVIPNI

NPVLLPKKTAEKAVKEPKSTTKATKSPKKAAAA*

>Ckanehirae_v3|CKAN_02766400|CKAN_02766400

MEATETIKLAGGRKGSTRKKAVSKSLKSGLQFPIGRISRFLKKGRYAQRFGFGSGAPIYL

AAVLEYLAAEVLELAVNAARDNKKIRIIPKPVPLAVRNDEELRKLLHGVTIAHGGVLPNI

HSVLLPKKTDKGNAEATKAPKSPKKD*

>Ckanehirae_v3|CKAN_00528400|CKAN_00528400

MEGGGKVKKGAAGRKGGGPKKKPVSRSVKAGLQFPVGRIGRYLKKGRYSQRVGTGAPVYL

AAVLEYLAAEVLELAGNAARDNKKNRIIPRHVLLAVRNDEELGKLLAGVTIAHGGVIPNI

NPVLLPKKTAEKAAKEPKSSSKAPKSPKKAAAA*

>Ckanehirae_v3|CKAN_00528500|CKAN_00528500

MEGGGKVKKGVAGRKGGGPKKKPVSRSVKAGLQFPVGRIGRYLKKGRYSQRVGTGAPVYL

AAVLEYLAAEVLELAGNAARDNKKNRIIPRHVLLAVRNDEELGKLLAGVTIAHGGVIPNI

NPVLLPKKTAEKAAKEPKSPAKAPKSPKKAA*

>Ncolorata_W7

MEAGGGGATGKVRKGAGGRRGGGGGLKKKPVSRSVKAGLQFPVGRIGRYLKKGRYSQRVG

TGAPVYLAAVLEYLAAEVLELAGNAARDNKKNRIIPRHFLLAVRNDEELGKLLSGVTIAH

GGVLPNINPILLPKKTQKETNEPKSPKSPKKS*

>Ncolorata_W7

MEGGKSVKASGGRKAGAKRSVVSKSVKAGLQFPVGRIARFLKKGRYAQRVGIGAPVYLAA

VLEYLAAEVLELAGNAARDNKKTRIIPRHVLLAVRNDEELGKLLAGVTIANGGVLPNIHS

VLLPKRTEKASQETKSPKSPKKA*

>Ncolorata_v1.2b|Nycol.E00338|Nycol.E00338.1

MEGSGTGKAKKGAGGRKGGGARKKSVSRSVKAGLQFPVGRIGRYLKKGRYSERVGSGAPV

YLAAVLEYLAAEVLELAGNAARDNKKNRIIPRHVLLAVRNDEELGKLLAGVTIAHGGVLP

NINPVLLPKKTEKATKEPKSPKATKSPKKA*

>Ncolorata_v1.2b|Nycol.F00084|Nycol.F00084.1

MEAAGGGTSGKLKKGAGGRRGGGAGPKKKPVSRSVKAGLQFPVGRIGRYLKKGRYAQRVG

TGAPVYLAAVLEYLAAEVLELAGNAARDNKKNRIIPRHVLLAVRNDEELGKLLSGVTIAH

GGVLPNINPVLLPKKKEKESMEPKSPKASKSPKKA*

>Aamericanus_v1.1|Acora.07G111300|Acora.07G111300.1

MEATGKAKKGAAGRRGGGPKKKPVSRSVKAGLQFPVGRIGRYLKKGRYAQRVGTGAPVYL

AAVLEYLAAEVLELAGNAARDNKKNRIIPRHVLLAIRNDEELGKLLAGVTIAHGGVLPNI

NPVLLPKKSASATTAAGKEPKSPSKAAKSPKKTPKKD*

>Aamericanus_v1.1|Acora.03G206000|Acora.03G206000.1

MEGTKTTKGAGGRKGGGARSKSVSKSVKAGLQFPVGRIARFLKKGRYAQRVGSGAPIYLA

AVLEYLAAEVLELAGNAARDNKKSRIIPRHLLLAIRNDEELGKLLSGVTIAHGGVLPKIH

QVLLPKKTGKSGEATPEGGATKSPKKSPKKNA*
