# Supplementary material for: Crosstalk between H2A variant-specific modifications impacts vital cell functions
Source: PLoS Genet. 2021 Jun 4;17(6):e1009601. doi: 10.1371/journal.pgen.1009601 (PMC8208582; doi:10.1371/journal.pgen.1009601)
Supplement: Attachment — Submitted filename: 113798_1_rebuttal_2052727_qs0m17.docx [file pgen.1009601.s010.docx]

Response to Reviewers:

We thank the three Reviewers for their insightful and constructive comments. We agree that certain aspects of the work do not answer specific mechanistic question relative to the function of KSPK phosphorylation and its regulation. However, we would kindly ask the Reviewers to consider that the goal of these experiments is primarily to get insight into potential mechanisms that explain how histone variants were selected during evolution. The preprint of this article was commented by Harmit Malik’s laboratory (<https://prelights.biologists.com/highlights/crosstalk-between-h2a-variant-specific-modifications-impacts-vital-cell-functions/>), which highlights further the broad significance of our work for researchers in evolution, chromatin, synthetic biology, plant biology and epigenetics.

Large sections of the manuscript have been re-written to make this clearer. Specifically, here we ask why segregation of functional motifs at the C-terminal tail contributed to separation of main classes of H2A variants. Our study focuses on the H2A.X-specific motif SQ and the H2A.W specific motif KSPK. We conclude that these two motifs do not usually coexist on a single H2A because PTMs on the motifs interfere with each-other’s function if they are gathered on the same C-terminal tail as in the unusual variant H2A.W.7 in Arabidopsis.

Reviewer #1 (Evidence, reproducibility and clarity (Required)): 

Schmuecker et al. investigate the function of the phosphorylation of two histone variants known to occur heterochromatin, i.e. H2AW6 and H2AW7. The endeavor starts by a detail mass spec analysis of posttranslational modification of these two variants revealing that H2AW6 is, among others, phosphorylated at a consensus CDK phosphorylation site. In contrast, H2AW7 has the very same site (KSPK) but appears to be not phosphorylated. Interestingly, H2AW7 has an SQ motif that is know to be phosphorylated the checkpoint kinases ATM and ATR and previous work has shown that H2AW7 is indeed needed during DNA damage response. Now, the authors explore the specificity of this phosphorylation pattern, i.e. raising the question why H2AW7 is not phosphorylated by CDKs. 

The key experiment is the demonstration that phospho-mimetic mutants of the KSPK (KDPK) in H2AW7 are hypersensitive to DNA damage as measured by plant growth (but see my comments below). Using yeast as a heterologous system, the authors then provide evidence that the BRCT-domain containing protein Mdb1 from yeast cannot bind to the KDPK variant of H2AW7. However, a Mdb1 homolog has not been identified in plants yet. 

My only major concern is that the authors always use growth as a proxy for DNA damage sensitivity. This is an often used and valuable assay. However, it is also a very complex assay that reveals little about the underlying cellular defects. Hence, I propose that the authors try adding some cellular parameters here. At one or more of the following parameter would make the damage more palpable and my help later to identify a Mdb1 homolog in plants. For example, do they see more cell death in planta? Is the growth reduction due to fewer cells? Do the authors find more DNA double strand breaks in their mutants or does the repair take longer? For instance are there more gammaH2A.X foci in KDPK-H2AW7 plants?

Response: We understand that Reviewer#1 would like to learn more details about the consequence of DNA damage. While this is interesting, we are focusing on measuring the resistance to DNA damage using a reliable simple functional assay. In addition to root growth assay (that we find less reproducible and less accurate to score), the true-leaf assay used in our study is classically used in many publications that analyse DNA damage response pathways.

For example : Han et al 2018 [*BMC Plant Biology*](https://bmcplantbiol.biomedcentral.com/) volume 18, Article number: 274 (2018);

Rosa et al., 2013 Plant Cell 25:1990-2001.

**Minor points** 

Figure 1: I found the green highlight too light.

Response: We are grateful for the feed-back and have enhanced the green tone.

Figure 4B: What does % true leaves mean? Is this area, cell size or number of true leaves? I assume that 100% is WT untreated? If so, please spell out.

Response: The “true leaf assay” is a most often used assay for testing plant mutants for DNA damage sensitivity. If DNA damage occurs and is not efficiently repaired, meristematic cells in shoot meristem are arrested and do not divide, hence plants do not produce the first pair of “true” leaves after cotyledons expand. In this assay cotyledons open and grow normally as they are already fully determined by the maturation of embryos in plant seeds.

In this assay the treated WT shows already a reduction of plants with true leaves as compared with untreated WT (100%). We added explanations in Materials and methods with a reference (Rosa et al., 2014).

Figure 5D: Please indicate the level of the serial dilutions used, e.g. 1, 1:10; 1:100, etc.

Response: This has been indicated in the Figure legend.

SFig4C: tg9 is an lab-derived allele name, I would simply call this cdka;1 +/- or if space is limited c+/- 
Response: Yes, this is correct. We changed allele names as suggested.

SFig4D: "CDKA;1 T161A" should read "CDKA;1 T161D". 
Response: We have corrected the name.


Reviewer #1 (Significance (Required)): 

The is an interesting paper that explores chromatin regulation during DNA damage in plants. The paper is signified by the strong biochemical exploration of histone variants and by that provides a new and complementary angle to many current DNA damage studies in plants. The findings are interesting even if a Mdb1 homolog cannot be found in plants so far and address an even broader question in molecular biology, i.e. why certain proteins are and others are not phosphorylated although they have similar motifs. In my opinion, this study opens new doors how to study DNA damage in plants. 

**Referees cross-commenting** 

While I agree that all comments of reviewer 2 and 3 are valid and would certainly strengthen the manuscript, I also think we should not get too excited here with asking for experiments. For instance, searching for an explanation why H2A.W7 escapes phosphorylation is tough. Finding such a mechanism could take years and likely relies on more than one regulatory step. In my opinion, it is clear that the story presented has open ends - as many studies. Still, it opens interesting and new questions and provides evidence for a new regulatory process, i.e. that CDK phosphorylation would interfere with the consequences of ATM/ATR phosphorylation and hence it is precluded. To me, this is a notable scientific advance and I would as this moment only ask for experiments that support the core message of the paper rather than opening a whole new battle field. 

Indeed, a direct and personal exchange would be very interesting and scientifically stimulating. 

I see the point of review 2 concerning the title and agree that the title can be improved, perhaps by adding the word "potential". I still think this is an interesting and novel scientific approach that the authors followed, i.e. why is a certain PTM not happening although it could occur. 

The link to mammalian H2A should also be adjusted as suggested.

Response: We clarified the link between H2A.W and macroH2A. See lines 330-340. 


Reviewer #2 (Evidence, reproducibility and clarity (Required)): 

Fred Berger and colleagues present a study that follows their successful and exciting line of research studying the role of plant H2A.W histone variants. In their previous work they have shown that H2A.W6 and H2A.W7 (W6 and W7, respectively) possess C-terminal KSPK motifs that contribute to heterochromatin compaction. In addition, W7 shares the SQ motif and a role in DNA damage signalling with the replication coupled (canonical) H2A. In the presented study they have investigated the role of phosphorylation of the KSPK motif (P-KSPK). 

Here I list the statements as I see them from the data and make a few comments. 

Figure 1-3: The KSPK motif of W6 but not W7 is phosphorylated (P-KSPK) in a cell cycle dependent manner, likely by CDKA. 
The in vitro and inhibitor evidence for CDKA is of intermediate strength and by itself does not allow the strong conclusion that 'H2A.W.6 phosphorylation is mediated by CDKA'. I suggest to move the genetic data into the main figure and to clarify if differences in cell cycle distribution might be an alternative explanation for the differences in P-KSPK.

Response: We thank Reviewer2 for the suggestions and we mentioned the potential side effect of treatments (line 198-203). We also moved the FigS4C as a main figure panel. We agree that on their own, each experiment does not allow to reach a tight conclusion. Yet, taking into consideration both in vivo and vitro assays, the lack of H2A.W.6 phosphorylation in mutant with low CDKA activity lead to the logical conclusion that cyclin kinases are responsible of the phosphorylation of this variant.

Part of figure 4: Mapping experiments suggest that the other amino acids in the C-terminus determine the P-KSPK phosphorylation. 
Can this explain the specific interaction of W6 but not W7 with CDKA in cells? In vitro CDKA does not discriminate but this can be expected for an in vitro experiment (Figure 2).

Response: We agree with Reviewer 2 and have specified this fact more clearly. At this point we do not find it necessary to perform a mutational scan across this region of H2A.W.6 because this would require a large number of transgenic lines and careful quantification of the levels of phosphorylation. However, we did comment on this idea in the Discussion line 347-349.

Figure 2: The KSPK motif phosphorylation is reduced upon induction of DNA damage (strand breaks induced by bleomycin).

Response: This is true but only if DNA damage treatment is prolonged (Figure 2B). As DDR results in cell cycle arrest, this observation led us to consider the role of the cell cycle associated regulation.

Figure 4: When mimicking the 'non-existent' or at least so far 'non-detected' KSPK phosphorylation on W7, the protein fails to rescue the hypersensitivity of the *hta.7* mutant to a different type of DNA toxin (zeocin, intercalating). 
The data as it stands does not support the conclusion and I honestly do not understand the rationale of the experiment. What about W6? 
Response: We apologize for not having clarified a specific point. At this point we question the importance of the absence of KSPK phosphorylation of H2A.W.7 in the context of the DDR. A *hta7* mutant background deprived of H2A.W.7 is more sensitive to DNA damage than WT. This loss of function mutant is rescued by H2A.W.7 and also by a non-phosphorylable KAPK form of H2A.W.7. However, a mutant form of the protein that mimics the phosphorylation (KDPK) does not rescue showing that the phosphorylation of the serine residue of the KSPK motif interferes with the DDR. Because DDR causes SQ phosphorylation of the H2A.W.7 KDPK form we conclude that the prevention of DDR by KSPK phosphorylation is mediated by an event downstream of SQ phosphorylation.

Figure 5: The addition of the W6 tail with the KSPK motif to SpH2A leads to phosphorylation and creates a hypersensitivity to another DNA damaging agent in yeast (MMS, alkylating). This is not the case when mutating the eliminating the entire motif. 
Is this because of the motifs function (eg in heterochromatin formation) or mediated by the phosphorylation? The use of phosphomimetics and unphosphorylatable mutants would help to clarify this?

Response: We thank Reviewer#2 for the suggestion and have carried out these tests. The loss of function *hta7* mutant was rescued by WT H2A.W.7 and also by a non-phosphorylatable KAPK form of H2A.W.7 (Fig. 4B). However, a mutant form of the protein that mimics the phosphorylation (KDPK) does not rescue the hypersentivity to DNA damage of the *hta7* mutant (Fig 4B). We concluded that the phosphorylation of the serine residue of the KSPK motif interferes with the DDR. Notably, mutations of the KSPK motif into either KAPK or KDPK did not affect SQ motif phosphorylation (Fig 4C).

The main issue however is that the KSPK phosphorylation on W6 does not seem to have a related phenotype in Arabidopsis.

Response: we did not use the same chemicals to assess DNA damage response in Arabidopsis and yeast. We have addressed this problem with treatment with bleomycin in yeast and obtained the same conclusion reached with more commonly used chemical (see new Figure panels 5D , S5E and S5F).

Figure 6: A genetic interaction was identified between the presence of the W6 tail and Mdp1 using different growth limiting conditions in yeast (HU). Mdb1 is known to bind to the C-terminal peptide of SpH2A. The data shows that this slightly reduced by the addition the W6 tail and much more if the W6 peptide is phosphorylated. 
Genetic screens are powerful in yeast but to generate strong data they need to be validated in the source species of the protein of interest, here Arabidopsis.

Response: We share the same concern. However, the ortholog of yeast Mdb1 has not been identified in Arabidopsis. This is why we used yeast to get insight in the mechanism explaining why H2A.W.7 should not be phosphorylated in Arabidopsis. Arabidopsis encodes at least four proteins, which could qualify as potential orthologs of Mdb1 based on the presence of BRCT domain and pattern of expression. Testing their function (which might be redundant) represents the equivalent of several years of work and is out of the scope of revisions.

**Minor points:** 

Please show / remind the reader about the relative dosage of W6 : W7 : total H2A pool.

Response: Both proteins are equally present in chromatin although at slightly distinct locations and the reader can refer to Yelagandula et al 2014 and Lorkovic et al., 2018 for details. We indicate this in the Introduction, line 66-70.

Please consider illustrating the relative abundance of the detected phosphorylations on 141 vs 155. Can you say what fraction of W6 is modified?

Response: We added information from spectral counting into Suppl. Figure 1 as referee #3 also requested this. Based on spectral counting we can only approximately say that 5% is phosphorylated on S129 and 18% is phosphorylated on S145. The new analysis of spectra led to conclude against phosphorylation at S141.

My expertise is mammalian cells and histone variants. The figures are difficult to read for someone not used to yeast and arabidopsis. Simplified names and a more consistent use of the nomenclature could help. 
'hta-6' or 'h2a.w.6' (legend and figure 1D) 
'gH2A.W.7' or 'H2A.W.7p' (both in Figure 2B, but also see my comment below)

Response: We are sorry about this but do follow strictly the histone nomenclature that has been agreed by the entire community. This nomenclature might be tedious to read but does prevent ambiguity and follows the logic of phylogenetic origin of variants -see Talbert et al 2012. Furthermore, in Arabidopsis genetics, mutants are indicated in lower case italics. Gene or mutant names are not always the same as protein names (for example *HTA7* is the name of the WT allele *hta7* is the name of a mutant allele and H2A.W.7 is the name of the protein). Here, we stick to rules for Arabidopsis nomenclature. Nevertheless, we did correct spotted inconsistences in labelling alleles and antibody names throughout the manuscript. See also comments below on antibody naming.

I have an issue with the naming of the antibodies: 
'a-H2A.W6p' is an interpretation of results but an incorrect name for an antibody that is in theory able to detect phosphorylated KSPK motifs on both W6 and W7. I suggest to use: anti-P-KSPK and clarify the specificity in legend and figure (P-S145 on W6 or W7). 
The use of gammaH2A is historical but becoming problematic now that we know of more phosphorylation sites on histones (of which several respond to DNA damage signals) and de facto have stopped using radioactivity to detect them. 
What about 'anti-P-SQ' if you clarify that this corresponds to the previously described P-S134 on W7 and that it was previously named as 'gammaH2A.W.7'. In yeast this corresponds to the 'S129p' shown in , right? This is again a different way of naming the site.

Response: We changed antibody name as suggested into P-KSPK. We indicated in the Figure legend to which Serine residue in W6 and W7 is this antibody specific.

We agree that use of gammaH2A.X is historical but it also refers to SQ phosphorylation in the C-terminal SQEF/Y motif of H2A.X and there is a tremendous number of papers referring to this phosphorylation as gammaH2A.X. Therefore, we believe that there is no need to change this name in this study but it could be worth to initiate a general discussion about this in the community. This name has also been agreed in Talbert et al., 2012.

Use of gammaH2A.W.7 is restricted to this histone variant in Arabidopsis and as we named it as such in our previous publication (Lorkovis et al., 2017). Change this name here would introduce confusion.

I suggest to illustrate the specificity of the antibodies in a cartoon in Figure 1.

Response: All variant specific antibodies are described in two previous publications (Yelagandula et 2014 and Lorkovic et al 2017), but for clarity we indicated now the peptides used to obtain variant specific antibodies in Figure 1C.

Would be good to clarify why H2A.W12 has not been included in the study. Does it lack the KSPK motif? Is it less abundant than W6 and W7 and can be neglected?

Response: H2A.W.12 is expressed only during the late phase of reproductive development and is far less abundant than the two other variants. There has been no detailed study of the function of this variant.

Not clear why the authors used a different DNA damaging agent in every main figure. Please explain.

Response: We consistently use either Bleomycin for treatment of seedlings or Zeocin for true leaf assay. These two agents produce DNA double strand brakes in similar ways, and we could show previously that levels of gammaH2A.X and gammaH2A.W.7 are similar when using these two agents (Lorkovic et al., Curr Biol. 2017). Zeocin was chosen for true leaf assays because of lower variance between batches.

Is there a possible explanation for the loss of S-129p a SpH2ACT-AA (Figure 5C)? Is the switch from threonine 121 to serine 122 phopshorylation robustly observed or a mistake in the drawing of the figure (Figure 5E)? Taken both observations together it seems that extensions screw up the interaction profile of the C-terminus of SpH2A (to varying extents).

Response: Antibodies recognizing S-129p presumably require intact C-terminus (COOH group). Due to the addition of W6 WT or mutated tail this is not the case anymore and the antibody does not bind to the epitope. This is also the case for SpH2A.W^At^ and SpH2A.W4A^At^ strains. Because of this we performed MS analysis on these strains to find out if SQ is phosphorylated (Figure 5E).

Does the SGA screen provide any evidence that CDKs mediate the phosphorylation of the chimera in yeast? Is the P-KSPK related to cell cycle in yeast?

Response: The KSPXK motif is the target for CDKs and other proline directed Serine/threonine kinases in all eukaryotes, including yeast.

Carefully reading the abstract I also have an issue with the statement suggesting relevance in mammalians by comparing to macroH2A: 'This motif is also found in macroH2A variants in animals and confers specific properties to the nucleosome.' Unfortunately, I had missed it before but truth is that the KSPK motif does not exist in macroH2A but only the related KSKKK. From the biochemical point of view the absence or presence of a proline after serine changes the specificity for entire classes of kinases. Phospho-MS data shows that this motif is rarely phosphorylated. The second half of the sentence on the motif's importance refers to the H2A.Ws and not macroH2A. All this can obviously be fixed by text modification.

Response: The abstract has been rewritten so the concern raised by reviewer#2 is fixed. We also checked the literature and find that in the linker region of macroH2A1 the similar motif KSPS with S137 is phosphorylated (see Bernstein et al 2008). It was also shown that the truncated macroH2A1 has heterochromatic properties (Seminal work from K Lueger’s lab and more recent work in Ladurner lab). So, we do think that the functional parallels between these two variants are justified.

Reviewer #2 (Significance (Required)): 

**Strength:** The identification of a new PTM occuring at a motif that is known to be relevant for H2A.W histone variant function in Arabidopsis. 

**Weakness:** I feel that the statements are not well connected and in some cases I do not understand the rationale for addressing some questions and neglecting others. Specifically, I do not understand why the authors have focused on DNA damage signalling in the first place since the P-KSPKK is not induced by DNA damage (Figure 2), it does not occur at all on W7 that has a SQ motif and there is no evidence in plants that the phosphorylation in W6 affects DNA damage or its repair. 

**Key questions that are not addressed are:** 
- What is the physiological role of the KSPK phosphorylation on W6 (that actually happens)? Does it affect its function in heterochromatin formation and cell fate descisions? Does it affect cell cycle progression and DNA repair? 
- Does anything trigger the same phosphorylation in W7 (maybe using a hta-6 background)? If yes, what is the function in this response. 
Response: So far, we could not detect H2A.W.7 phosphorylation in planta. We used *hta6* mutant background in Figure 2A, but we did not detect any phosphorylation of H2A.W.7 either without or with induction of DNA damage. If any other condition can induce H2A.W.7 phosphorylation at KSPK motif is not known.

**Referees cross-commenting** 

The finding that 'KSPKKA phosphorylation in H2A.W.7 incapacitates the function of the H2A.X-like SQ phosphorylation.' is an interesting observation. It would be relevant, if the phosphorylation would be detected in plants in the first place. In the absence of this evidence, I feel that detailed mechanistic studies lack their rationale. How do you evaluate the value of a PTM that 'might' occur? 
I think the manuscript would also gain value if the authors' would be able to explain how H2A.W7 avoids the KPSKKA phosphorylation occurring on H2A.W6. Hypothetica mechanisms: No interaction with CDKA in cells, rapid turnover by a phosphatase. 
I have to they that this interaction here is an interesting experience for me and I would love to really talk with my fellow co-reviewers. I agree with reviewer 1 in that we should not ask for an entire new project but rather help the authors to strengthen the core message. The issue I have is that I do not agree with the current core message as stated in the title 'Crosstalk between H2A variant-specific modifications impacts vital cell functions' as the crosstalk between SQ and KSPK phosphorylation does not occur in the species of origin. 

But this could be solved by adding the word 'Potential' at the beginning.

Response: As other referees also suggested changing the Title, we did this accordingly.

We clearly show that phosphomimic mutant of H2A.W.7 confers sensitivity to DNA damage although phosphorylation of both H2A.X and H2A.W.7 at SQ motifs is not affected (Figure 4). Thus, the crosstalk does occur in Arabidopsis as well. This result corroborates with our observations in synthetic approach with fission yeast where phosphorylation of the KSPK motif does not prevent SQ phosphorylation but renders this strain sensitive to DNA damage. Unfortunately, we are not able to demonstrate that negative charge at the KSPK motif interferes with recognition of phosphorylated SQ motif on H2A.W.7 because the reader of this phospho site is not known in Arabidopsis (or in plants in general). We clearly state lin 360-362.


Reviewer #3 (Evidence, reproducibility and clarity (Required)): 

**Summary:** 

The authors present a technically sound study investigating PTMs of two heterochromatic H2A variants in plants, H2A.W.6 and H2A.W.7, of which the latter is one of the H2A.X-like variant in plants known to be phosphorylated upon DNA damage (at a C-terminal SQ motif). H2A.W.7 has a further C-terminal extension ending in a KSPKKA motif also shared with H2A.W.6. The KSPKKA motif is found to be phosphorylated and a cell-cyle dependent kinase, CDKB;1, is identified that can phophorylate KSPKKA motif. The authors report the surprising finding that only H2A.W.6 but not H2A.W.7 is phosphorylated in plants/plant cells despite CDKB;1 can in vitro phosphorylation both variants. Via introduction of H2A.W.7 in yeast the authors demonstrate that if phosphorylated at the KSPKKA motif, H2A.W.7 can no longer serve its role in DNA damage response. This is particularly surprising since H2A.W.7 can acquire the DNA-damage dependent phosphorylation normally even in the presence of a phosphorylated adjacent KSPKKA motif. Performing a genetic screen the authors then identify yeast Mdb1 as a putative effector of phosphorylated H2A.W.7 after DNA damage. Mdb1 binding appears to be sensitive to adjacent KSPKKA phosphorylation, leading the authors to the final conclusion that KSPKKA phosphorylation of H2A.W.7 must be prevented to retain it's H2A.X-like function in DNA damage response. 

**Major comments:** 

While the data presented is overall robust and the mechanistic model is supported by the data, the study ends somewhat abruptly, leaving some key questions open: 

- while the authors provide a mechanistic connection between the two phosphorylation sites, the reason behind these evolutionary conserved arrangements of two phosphorylation sites, one overriding the DNA damage signaling capabilities of the other, remain completely unclear.

Response: We realise that this point is not highlighted clearly in the manuscript and have rewritten parts of the introduction and discussion to bring to light the fact that interference between motifs function exists points to the fact that the motifs should be isolated on distinct H2A, thus proposing a cause for evolution of distinct H2A variants.

- while interesting, the functional importance of cell cycle dependent KSPKKA phosphorylation has not been addressed as a I understand.

Response: this is in part true, the work does not address the positive function of KSPK phosphorylation on H2A.W.6 but it shows that this PTM has inhibitory effect and demonstrates how this effect is likely mediated (inhibition of the recognition of the SQ phosphorylation by Mdb1).

- KSPKKA phosphorylation in H2A.W.7 incapacitates the function of the H2A.X-like SQ phosphorylation. Why then does H2A.W.7 carry the KSPKKA in the first place. What is the functional relevance of the KSPKKA motif. Maybe I missed this but does deleting the KSPKKA motif from H2A.W.7 entirely cause any known phenotype in Arabidopsis?

Response: From our previous work we know that the C-terminus of H2A.W is in close contact with the linker DNA, thus protecting more DNA then for example H2A.Z (Osakabe et al. NAR 2018; Lei et al., Curr. Biol. 2020). Deletion of H2A.W.7 impairs the DNA damage response (Lorkovic et al 2017) and here we show this DNA damage sensitivity cannot be fixed by complementation with H2A.W.7 deficient for KSPK.

Deletion of entire H2A.W.7 does not result in any visible phenotype in Arabidopsis and does not cause major changes in transcription (please see Bourguet et al doi: https://doi.org/10.1101/2020.03.19.998609)

- the mechanism how H2A.W.7 is prevented from being KSPKKA phosphorylated is puzzling and not resolved in the present manuscript. While this motif shared with H2A.W.6 is readily phosphorylated in vitro and H2A.W.6 is phosphorylated in vivo. How about searching for a H2A.W.7-specific phosphatase. Could it be as simple as incubating the in-vitro-phosphorylated H2A.W.6 and H2A.W.7 histones with cellular extract and check if the phosphorylation is removed?

Response: This is of course of interest but this is not the point of the work and to do this properly would require an entirely distinct project.

- I was a really neat idea to take the problem into yeast. The synthetic screen identifies impairment if Mdb1 binding as the potential cause of the DNA damage sensitivity of plants and yeast with phosphorylated/phosphomimick H2A.W.7. Nevertheless, it seems only worthwhile doing the screen if the finding is subsequently validated in plants. As I understand from a comment in the manuscript, the Mdb1-ortholog is not known in Arabidopsis, however I could find two proteins with sequence similar enough to Mdb1 to be good candidates: NP_188785.2, CAD5323742.1. The former is already named BRCT domain-containing DNA repair protein and also appears in Uniprot (Q9LVF7_ARATH).

Response: We do validate the results in yeast by the genetic complementation experiments with mutant forms of H2A.W. 7. If Mdb ortholog were know in plants, we would not use the synthetic approach in yeast. Arabidopsis encodes for at least five proteins, which are potential orthologs of Mdb1 (MDC1) based on the presence of BRCT domain and pattern of expression. Testing their function (which might be redundant) represents the equivalent of several years of work and is out of the scope of revisions.

- Abundance of PTMs, could the authors please supply more information on spectral counting, and include the estimated abundance in SFig 1

Response: This has been included in Supp. Figure 1.

**Minor comments:** 

- Figure 2B bottom graph: H2AW.7.p should be gamma H2A.W.7

Response: Thank you for spotting this. We have corrected the mistake.

- in general I believe quantitation of single WB experiments is pointless, if the aim is to take the data to a more quantitative level, then triplicate measurements should be performed and summarized in the graph. 
Response: Thank you, We have summarized all experiments in a single graph.

-------------------- 
Simon Elsässer 


Reviewer #3 (Significance (Required)): 

- the manuscript provides the first comprehensive mass spec analysis of two plant histone variants. (The mass spec data could thus be presented in more detail!) 
- the manuscript advances our understanding of the function of these variants nad partially rationalizes why so many different variants are evolutionary conserved in plants. 
- because the mechanistic hypotheses haven't been entirely corroborated, the manuscript falls short of its potential in this aspect. 
- for the scientists outside of the plant field, the paper could add more detail on the evolutionary conservation of the H2A variants and motifs, and also explain better in the discussion how the new insights adds to and fit together with the existing knowledge (e.g. these are in heterochromatin, right? So is H2A.W.7 particularly important to mediated DNA damage response in heterochromatin, and why does it then 'need' the KSPKKA if it inhibits its important role in DNA damage response). 

-------------------- 
Simon Elsässer 


**Referees cross-commenting** 

I think the critiques put forth by the two other reviewers are valid and should be addressed as well. I think there is an agreement that the functional relevance of the yeast studies should be addressed in plants and clarified why the 'non-phoshporylation' is important. 
@Reviewer #1 I can agree with that. I'm not a plant person so it is hard for me to judge the feasibility and time needed for e.g. following up the Mdb1 in planta. 
